# Assessment of Population Relevance of Endocrine-sensitive Apical Endpoints in Fish Chronic Studies Using Individual-Based Models

**DOI:** 10.1021/acs.est.5c03364

**Published:** 2025-09-30

**Authors:** Alice Tagliati, Charles R. E. Hazlerigg, Edward R. Salinas, Laurent Lagadic, Thomas G. Preuss

**Affiliations:** † Enviresearch Ltd., Newcastle-Upon-Tyne NE1 4DP, U.K.; ‡ School of Natural and Environmental Sciences, Newcastle University, Newcastle-upon-Tyne NE1 7RU, U.K.; § 39092 Bayer AG R&D, Crop Science Division, Monheim am Rhein 40789, Germany

**Keywords:** fish, endocrine disruption, hazard assessment, effect models

## Abstract

Population models have long been thought of as a suitable approach for assessing the population relevance of chemical effects observed on individuals in laboratory studies, although they have rarely been applied in a regulatory context. We modeled potential population-level responses of individual-level adverse effects induced by endocrine-disrupting chemicals (EDCs). We imposed three effect durations (10-year, 3 months summer, or winter) for six common EDC endpoints (fecundity, fertilization rate, sex ratio: male and female skew, courtship and nesting behavior) at four magnitudes of effect (10, 20, 50 and 90% reduction) using individual-based population models for three fish species with differing life histories: stickleback (*Gasterosteus aculeatus*), brown trout (*Salmo trutta*) and zebrafish (*Danio rerio*). The suitability of different assessment criteria for determining the significance of population responses was evaluated. For all endpoints tested individually, effect magnitudes of 20% did not result in any population-level responses in each of the three species, except in the stickleback, where a 20% reduction in fecundity or fertilization rate led to population declines (in these cases, effect magnitudes of 10% did not result in population-level responses). Once standardized, our “exposure-agnostic molecule-independent” approach will enhance our understanding of population outcomes within the regulatory hazard-based assessment of EDCs.

## Graphical abstract

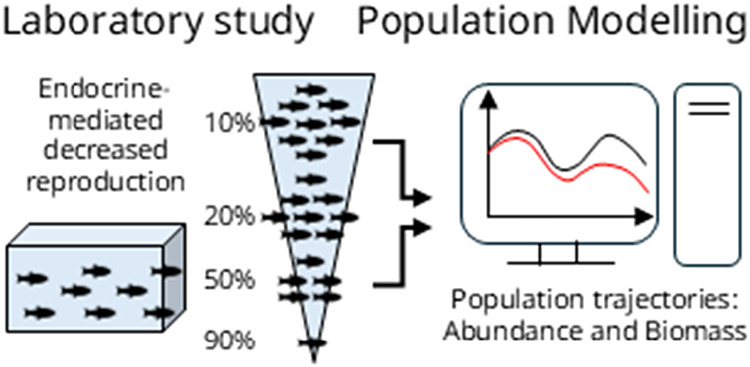

## 1 Introduction

Population models may be used to link the effects of chemicals observed in individuals to those of whole populations. Though different modeling approaches exist (e.g., differential equations, matrix models), each with their own strengths and weaknesses (see Forbes et al.^1^ for a detailed review of model types used in chemical risk assessment) we focus here on the use of individual-based models (IBMs). IBMs are spatially explicit population models that allow population dynamics to emerge from the life-history traits, behavior, and interactions between individuals.^2^ IBMs incorporate ecological processes and life-history strategies, including interactions between competing/cooperating individuals within single or interlinked populations. They also allow for the incorporation of physiological and behavioral endpoints following exposure to a chemical.^3^ As such, they have long been thought of as a suitable approach for assessing the population relevance of chemical effects observed in laboratory studies on individuals. Indeed, IBMs can be coupled to submodels in order to increase physiological and ecological realism, which makes them relevant for exploring which effects on individuals are likely to have the greatest effect on populations. The number of IBMs developed to assess the risk to populations from chemical exposure in various aquatic species is extensive, e.g., *Lemna* spp.,^4^ *Daphnia magna*,^5^ *Asellus aquaticus*,^6^ *Chironomus riparius*,^7^ *Gasterosteus aculeatus*;^8,9^ and*Danio rerio*,^10^ among others. However, a review of regulatory submissions for approval of pesticidal active substances in the EU between 2011 and 2021 found that only four have so far been applied in a regulatory context.^11^ This disconnect between the extensive academic progress yet slow regulatory uptake signifies fundamental challenges remain when applying these models to regulatory chemical risk assessment. Furthermore, other (more traditional) approaches are available to assess the population relevance of chemical exposure in a risk assessment (e.g., field studies and monitoring studies). However, these become far more technically and ethically challenging when attempting to perform them within the regulatory situation with endocrine-disrupting chemicals (EDCs) in Europe, where exposure is not accounted for and a hazard-based assessment is required.^12^ In such a situation, the use of population models has been identified as the most promising way to assess population responses.^13^ Hence, we focus throughout this paper on the use of population models specifically in the hazard-based population-level assessment of EDCs.

The World Health Organization (WHO) definition of an endocrine disruptor has been used as the basis for the implementation of regulatory assessment of endocrine-disrupting properties of natural and man-made chemicals that can affect human health or organisms in the environment. An endocrine disruptor is an exogenous substance or mixture that alters function(s) of the endocrine system and consequently causes adverse health effects in an intact organism, or its progeny, or (sub)­population.^14^ This definition has been transposed into regulatory endocrine disruption assessment programs in Japan, the US, and Europe.^15–18^ In Europe for example, if a pesticidal active substance (i) shows an adverse effect in nontarget organisms, (ii) has an endocrine mode of action (*i.e*., it alters the function(s) of the endocrine system), and (iii) the adverse effect is biologically, plausibly linked to the endocrine mode of action, this substance shall be considered as having endocrine-disrupting properties, *unless there is evidence demonstrating that the adverse effects identified are not relevant at the (sub)­population level for nontarget organisms*.^18^ This shows the essentiality of conducting a population-level assessment of endocrine-mediated adverse effects observed in individuals under laboratory test conditions. The European Chemical Agency (ECHA) and the European Food Safety Authority (EFSA) guidance in support of the implementation of the European regulation on the identification of endocrine-disrupting properties of pesticides provides orientations, but no recommendations, on how the population relevance of individual-level adverse effects could be addressed.^19^ Field and monitoring studies represent possible approaches, but they have considerable limitations (e.g., how to perform a field or monitoring study when the hazard-based assessment precludes chemical exposure to be included), which impedes practical implementation.^12,19^ The other, more promising approach relies on the use of population models, including IBMs. As such, population modeling of EDCs provides a suitable opportunity to not only address a regulatory issue but also to explore some fundamental aspects of model design, implementation, and evaluation common to all uses of population models for chemical assessment.

There have been previous efforts to model the population response following exposure to EDCs. For example, Hazlerigg et al.^10^ developed an IBM to evaluate the population-relevance of changes in sex ratio caused by androgenic (dihydrotestosterone) and estrogenic (4-tert-octylphenol) substances in zebrafish (*D. rerio*). These past efforts are not always constrained to IBMs either, with Miller et al.^20^ using a matrix model of the fathead minnow (*Pimephales promelas*) to assess population responses following exposure to fadrozole. However, irrespective of the precise modeling approach taken, these efforts have assumed a risk-based assessment, incorporating some form of chemical exposure in the models, while the current EU regulatory framework for EDC assessment necessitates a hazard-based assessment. In a recent comprehensive review of population modeling of EDCs, Hazlerigg et al.^13^ concluded that there were currently no examples that were completely consistent with the hazard-based assessment required in the current EU regulatory scheme, but identified essential points that must be addressed in order to perform such modeling. We used these points in developing our simplified modeling approach in this paper. Specifically, we did not consider a certain molecule and its effects but rather modeled different magnitudes of effect on different apical endpoints of interest in an EDC assessment. This means that all population modeling outcomes could be applied to any substance where the individual-level effects of the substance are known and a population-level hazard assessment is required.

Individual-level apical responses on survival, growth, development, and reproduction in single species are generally regarded as relevant for the maintenance of wild populations.^19,21^ Apical endpoints that describe these processes are therefore the starting point for population modeling. Apical endpoints directly measure outcomes of whole-organism exposure in *in vivo* tests, typically death, reproductive failure, or developmental impairment, whereas *in vitro* responses and suborganism-level responses, including biomarkers such as vitellogenin plasma concentration, are nonapical, mechanistic endpoints corresponding to intermediate key events at levels of biological organization below that of the apical endpoint that defines an adverse outcome in the adverse outcome pathway (AOP) concept.^22,23^ Fecundity, for example, is a relevant apical endpoint to describe an adverse outcome related to reproduction failure in several AOPs (*e.g*., AOPs 23, 25, and 30,^24^). Such individual-level adverse outcomes can be used to extrapolate effects to populations when used in conjunction with IBMs and to determine effect thresholds for adverse population-level outcomes.^12^ In this paper, we consider endpoints that are sensitive to perturbations of the endocrine system as described in the OECD Guidance Document 150 ^25^ and summarized by Marty et al.^26^ Specifically, which individual-level endpoints most strongly drive population declines and at what magnitude of effect will adverse population responses be observed.

Using these apical endpoints in population modeling requires a method to evaluate when a population response is relevant. Historically, a population effect has been considered relevant by using a threshold identified *a priori* for deviation of the mean of the treated population from the mean of the control. For example, Mintram et al.^8^ used a threshold of 15% deviation from the control mean population as showing a population response. However, this is arbitrary (different threshold values have been selected in the literature) and does not consider the importance of the dynamics and variability within a biological system. Therefore, the recent EFSA^27^ publication proposes two novel assessment criteria for determining whether a population response is observed or not. Specifically, these state that (i) the exposed population mean should not fall below the lower 95th percentile of the control and (ii) the lower 95th percentile of the exposed population should not be consistently below the lower 95th percentile of the control population. These are based on the concept of the Normal Operating Range (NOR), which is generally defined as the range of values enclosing the 95th percentile of the population of reference values.^28^ With regard to chemical risk assessment, EFSA^29^ stated that the NOR corresponds to the acceptable limits or range of values of a measurement endpoint that is normally observed during a predefined period in the reference ecosystem. In the current context, this range corresponds to the 95th percentile of the population’s abundance/biomass. As the NOR emerges from the model simulations using a certain variability in parameters and stochasticity in processes and this variability and stochasticity differ between models and species, different values for the NOR will be observed between models. This is the first time that a chemical guidance document has attempted to express how a response can be assessed in this way by using population models. However, the two EFSA^27^ assessment criteria have not been thoroughly tested in practice. As such, we explore the use of these criteria in the current work and assess whether they are fit-for-purpose.

Another general topic of interest regarding the use of population models in a hazard assessment is related to the effect duration that should be imposed (given that an exposure profile cannot be used). The duration of the effect imposed at the individual level could have a considerable bearing on the population response. For example, a 10-day intermittent effect resulted in a lesser population response than a 1-year continuous effect in the zebrafish.^10^ To capture this, Hazlerigg et al.^13^ recommended a 1-year duration of effect, accounting for aspects of chemical persistence, agronomic practices, and vertebrate seasonal breeding cycles. The influence of the duration of effect may also be a consequence of the apical endpoint affected and the timing of the life stage affected. For example, effects on reproductive endpoints are likely to have a higher population relevance if imposed during a species’ reproductive window. Hence, we further explore here the differences in population response with the timing and duration of imposed effects.

In this article, we present an “exposure-agnostic molecule-independent” approach to evaluate the relevance of individual-level adverse effects for fish populations using IBMs. As such, the approaches outlined here and the outcome of the simulations are the first step to creating a so-called “look-up table” for any molecule for which a population-level assessment for endocrine-mediated adverse effects is desired. During the course of this work, it became clear that there were critical decisions to be made on how to perform and interpret such modeling before such a look-up table could be compiled. As such, this paper therefore concludes with some generalizations about the population relevance of different apical endpoints at the individual level but also, more fundamentally, identifies and addresses some of the outstanding issues with regulatory use of population models. Specifically, how the timing and duration of effects imposed in hazard assessments may affect the population-level outcome, and second, whether the EFSA^27^ population-level assessment criteria are fit-for-purpose.

## 2 Materials and Methods

### 2.1 Selected Apical Endpoints

Apical endpoints used in this study were selected based upon the combination of (i) their sensitivity to modulation by endocrine activity, (ii) their importance in population dynamics and sustainability,^26^ and (iii) the ability to include the endpoints in the population models available. The apical endpoints related to fish reproduction that were used in all IBMs described below are fecundity, fertility, and sex ratio (male and female skewed) (SI, Table S1). In addition, behavior (courtship and nesting) was included in the three-spined stickleback IBM. All six endpoints are considered as sensitive to, but not diagnostic of, hormonal-mediated activity.^18,19^

### 2.2 Species and Model Selection

Three population models were selected from the literature for the three-spined stickleback (*G. aculeatus*) by Mintram et al.,^8^ brown trout (*Salmo trutta*) by Railsback et al.,^30^ and zebrafish (*D. rerio*) by Hazlerigg et al.^10^ A review of 579 European freshwater fishes identified the three-spined stickleback and stream-resident brown trout as two of 27 species considered highly susceptible to pesticide exposure due to their tendency to inhabit edge-of-field water bodies.^31^ In a follow-up study, the authors developed matrix models for 21 freshwater fishes considered most susceptible to pesticide exposure to determine their population resilience and sensitivity of different life-history traits.^32^ The stickleback was identified as one of the most vulnerable of all species tested in terms of its fertility, its relatively low fecundity, and very low fry and larval survival (average annual survival for females in the first year (*S*_0_) = 0.01). Indeed, the stickleback had the second lowest fry and larval survival rates out of the 21 species tested, with the only species with a lower early life-stage survival rate (the burbot *Lota lota*) compensating for that lower fry and larval survival rate with a mean absolute fecundity ∼47 times greater than that of the stickleback. Meanwhile, brown trout populations showed moderate vulnerability to individual-level effects, primarily a consequence of low juvenile survival. However, they also had the second lowest mean absolute fecundity (264 eggs/female/year) of all the species tested, making them particularly vulnerable to effects on reproductive endpoints. While there may be other species that could be considered more vulnerable (e.g., the European minnow *Phoxinus phoxinus*), there are no IBMs currently available to perform the current study with these other species. As such, the three-spined stickleback and the brown trout are considered appropriate focal species for use in this study covering different life-history strategies. The stickleback and trout were also selected as they cover fish populations in quite different water bodies (i.e., a small pond for stickleback and a larger stream for the trout). The zebrafish, a tropical fish, was out of the scope of the review by Ibrahim et al.^31,32^ Nevertheless, in the study of Brown et al.,^33^ which compiled a population survivorship index for various fish species utilizing data from existing scientific literature, zebrafish was predicted to be more susceptible than fathead minnow to population decline following exposure to environmental stressors because of its broadcast spawning behavior and lack of parental care. As such, the zebrafish was selected as it has a different life history, providing a good comparison to the temperate species.

Full details of each model are available in the relevant original publications, but each is briefly described here:

Stickleback model^8^—The model runs on a daily time step with the spatial extent of a 20 m^2^ pond. Resident stickleback progress through their lifecycle from eggs to larvae, juveniles, and then adults. Each time step, some fish die, while others grow, develop, and reproduce. The reproductive behavior of the fish (nest building and mate finding) is detailed and based upon dominance hierarchies and is an important feature of the model. Density dependence is included in fish growth, with environmental conditions implicit in the parametrization of the submodels. No immigration or emigration is included. The model was calibrated using the growth function to match the densities of wild populations and result in reproductively mature fish present during the spawning period (May–July). Validation included the satisfactory comparison of model outputs of population abundance compared against census data from two separate ponds in England.

Zebrafish model^10^—The model runs on a daily time step with the spatial extent of a 20 m^2^ pond. Zebrafish progress through their lifecycle from eggs, to larvae, juveniles, and then adults. Each time step, some fish die, while others grow, develop, and reproduce. Dominant adults mate, leading to successful reproduction. Density dependence is included in both growth and survival submodels, while environmental conditions of the ponds are implicit in the parametrization of these submodels. The model was calibrated using the predation rate to wild population data, while validation included the satisfactory comparison of model outputs to the size distribution of zebrafish found in the field.

Trout inSTREAM model^30^—The model runs on a quarterly time step (dawn, day, dusk, night) with a spatial extent of a stream reach. Trout progress through their lifecycle from redds containing eggs to juveniles and adults. Each time step, some fish die, while others grow, develop, and reproduce. The detailed movement decisions of the trout consider the trade-off between risk of death (predation and starvation) against growth and future contribution to the population. The decisions of one fish lead to interactions and affect decision-making in other fish (e.g., selection of habitat). Different processes were used to calibrate the model, including the rate of terrestrial predation and food availability from drift, while multiple different validation exercises have been undertaken, including pattern-oriented modeling approaches on submodels and whole population abundances against field data.

Each model was selected as the code was publicly available, they were developed from fundamental biological principles, included essential ecological processes, and were validated against an independent data set. As such, they were generally consistent with the EFSA^34^ checklist on Good Modeling Practice. In each case, the environmental scenarios in each of the published models were retained in this project, with the addition of a toxicity module (see next section) to investigate the desired endocrine-mediated adverse effects.

### 2.3 Implementation

The stickleback, brown trout, and zebrafish models were implemented in NetLogo v 6.1.1. A toxicity submodel was added to each population model to impose the desired effects to be investigated. Full details of the code amendments are provided in the Model Logs in the *Supporting Information* (SI). Hypothetical magnitudes of the effect were used in this project. Specifically, changes in each of the selected endpoints by 10, 20, 50, and 90% were implemented individually (SI, Tables S2 and S3). These values were selected as a balance between modeling effort and coverage of the magnitude of effect that has been observed in laboratory studies. For the sex ratio, these effects were imposed in both directions (masculinization and feminization). For example, a 50% reduction in females would result in a 25:75 female:male ratio from the 50:50 female:male model default ratio used in the control simulations. Further simulations were also performed to implement simultaneously a 10% effect on fecundity, fertility, and sex ratio (male and female skew). All effects were imposed directly on an individual endpoint, taking no account of the AOP that would lead to the effect on the apical endpoint, but considering that the effect was proven or suspected to be endocrine-mediated. This is a simplifying assumption, made in order to provide a general methodology that could be applicable to all known or presumed EDCs and suspected EDCs according to the Delegated Regulation (EU) 2023/707,^35^ but also because the AOP is not always obvious.^36^ After a spin-up period of 3 years to allow the modeled populations to stabilize, the effects were imposed for three different durations: (1) for a continuous period of 10 years associated with the duration of active substance approval commonly used within EU Regulation 1107/2009,^37^ (though considered unrealistic and worst-case for agrochemicals as it does not account for agronomic practices such as Integrated Pest Management, nor declines after application expected due to restrictions on substances considered persistent), and for seasonal exposure periods of 3 months either in the summer (May-Jul) (2) or in the winter (Oct-Dec) (3) every year for 10 years. The seasonal windows were selected to coincide with the spawning seasons of stickleback and trout. The stickleback and trout were modeled for all three durations, while the zebrafish was only modeled for duration 1 (as the zebrafish model includes continuous, not seasonal, reproduction, and the initial simulations indicated that it was less sensitive than the other two species modeled). We did not use any assessment factors or include any potential for individuals to repair the damage from the hypothetical endocrine-disrupting effect in the models, as these are not consistent with the current EU Regulatory Framework. The number of simulations performed for each scenario with each model differed, with 20 runs used for the brown trout and 70 runs used for the stickleback and zebrafish models. These numbers were selected as they represented a suitable number of simulations necessary to achieve a consistent mean population abundance. When using a NOR-based assessment criterion, it is essential that an appropriate number of model replicates are used. In particular, too few replicates could lead to highly variable population abundances (wider NOR) and inconsistent outcomes for the population effects. Further discussion is provided in the SI.

### 2.4 Assessment and Simulation Experiment

The outcome of the simulations was analyzed in terms of changes in abundance and biomass. To evaluate the population response, the mean and lower 95th percentile (confidence limit) were calculated from the model runs. Means and confidence limits were compared to those of the control simulation (no effects on any endpoints imposed) using the two assessment criteria from EFSA.^27^ As mentioned previously, this states that (i) the exposed population mean should not fall below the lower 95th percentile of the control and (ii) the lower 95th percentile of the exposed population should not be consistently below the lower 95th percentile of the control population. For the first criterion, should the mean of the exposed population fall below the lower 95th percentile of the control, then a population effect has been observed for this criterion. When analyzing the model results against the second assessment criterion, further guidance was sought from EFSA^27^ about what constitutes a population-relevant effect. In the text of that document, it states that *“···(ii) the lower 95% confidence limit of population abundance for treatment simulations is not consistently lower than that of control simulations (e.g., lower on a limited number of isolated data points only).”* Therefore, should the lower 95th percentile of the exposed population fall below the lower 95th percentile of the control population on more than a limited number of isolated data points, then a population-level effect has been observed for this criterion. A similar approach developed for this study using the upper confidence limit was also used, where the upper 95th percentile of the exposed population should not be consistently above the upper 95th percentile of the control population. Validation of the ecological model (where no ED-mediated effects are imposed) has been performed by the original model developers and was briefly mentioned earlier. Further validation of the model outputs presented in this paper once ED-mediated effects are imposed are not presented. There are still considerable practical and ethical challenges to identify/generate field data suitable for validating models where exposure cannot be considered *(see* ^13^).

## 3 Results

### 3.1 Assessment Criteria

When analyzing the model results against the second assessment criterion, the use of the strict interpretation of the assessment criterion meant that 100% of all the scenarios tested failed this second criterion (as the lower 95th percentile of the affected simulation fell below the lower 95th percentile of the control simulation on more than a limited number of isolated time points). That is, in all cases, a population response was observed. Furthermore, additional control simulations (that should therefore be consistent between themselves and should not result in a population effect) would also fail this strict interpretation of the criterion because it is expected to have approximately 50% of daily time points with a lower 95th percentile confidence limit below that of the first control. This is clearly demonstrated in Figure 1, which shows the mean and lower 95th percentile confidence limits that resulted from two sets of control simulations for zebrafish. The numerical results for the two EFSA^27^ criteria from the pairwise comparison of three stickleback control simulations (i.e., with no effects imposed during the 10-years period) (SI Table S4), showed that all comparisons did not pass the second criterion for a high number of days (between 1222 and 4500 for abundance, and 1303 and 4459 for biomass, on a total of 4745 days simulated). Therefore, the second criterion from EFSA^27^ was not used to evaluate the population response in this study, and all results are assessed against criterion 1 only.

**Figure 1:**
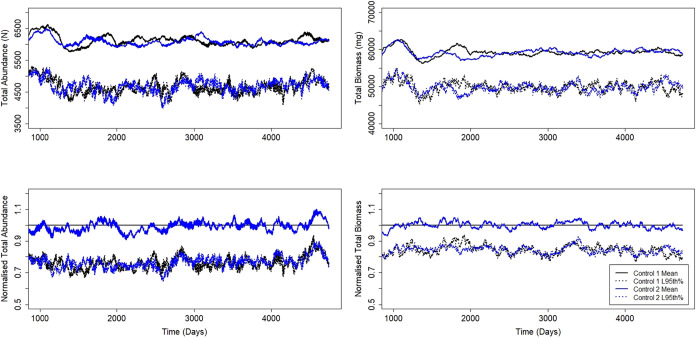
Mean and lower 95th percentile confidence limits for total abundance and biomass for two sets of control simulations (70 simulations in each) with the zebrafish model (top, absolute numbers; bottom, normalized against the first control). Note the lower 95th percentile lines from one set of simulations are constantly crossing those from the other, indicating two sets of control simulations would indicate a population response if strictly adhering to the EFSA^27^ second assessment criterion (the lower 95% confidence limit of population abundance for treatment simulations is not consistently lower than that of control simulations, e.g., lower on a limited number of isolated data points only).

### 3.2 Population Relevance of Different Endpoints

No population-level effects were observed in any simulation where the magnitude of the individual-level effect was 10% imposed for a single apical endpoint (Tables 1 and S5). A 20% effect magnitude on all endpoints is still compatible with the maintenance of zebrafish and trout populations. For the stickleback, 20% reduction of fecundity and fertilization success has an impact on the modeled population, while such an effect level on sex ratio and courtship and mating behavior does not impact the population. With magnitudes of effect greater than this, the population response differed depending upon which individual-level endpoint was affected. In general, for all species modeled, the fish populations were most sensitive to effects on fecundity and fertilization rate (e.g., Figure 2). The modeled populations were moderately sensitive to effects on courtship behavior in the stickleback and sex ratio, male and female skew in all species, while effects on the stickleback nesting behavior resulted in no population-level effect at any of the magnitudes tested. For the endpoints to which the modeled population was most sensitive, the year-round continuous implementation of individual-level effects led to observable population-level effects when a magnitude of effect of 20% on the apical endpoints (fecundity and fertilization rate) was implemented. This rose to 50% for sex ratio and courtship behavior, while even a 90% disruption of nesting behavior did not result in any population-level response. This picture changed once multiple endpoints were all affected in a single simulation. In the combination scenario, where a 10% effect magnitude was imposed on fecundity, fertilization rate, and sex ratio, a population response was observed in the stickleback and trout (SI Table S6).

**Figure 2:**
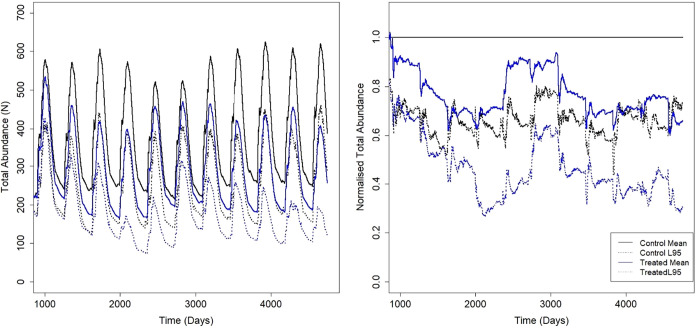
An illustrative example of the model results. Trout total abundance (from 20 simulations) across a 10-year treated period with a 50% reduction in fecundity (left, absolute numbers; right, normalized against the control). An initial model “spin-up” period of 1095 days (3 years) used the control parametrization, then the 50% reduction in fecundity was imposed on all fish in the model for the remaining duration of the simulations. The average total abundance of the treated population fell below the average of the control simulation at a number of points (e.g., day 3900), thereby identifying an adverse effect on the population.

**Table 1:**
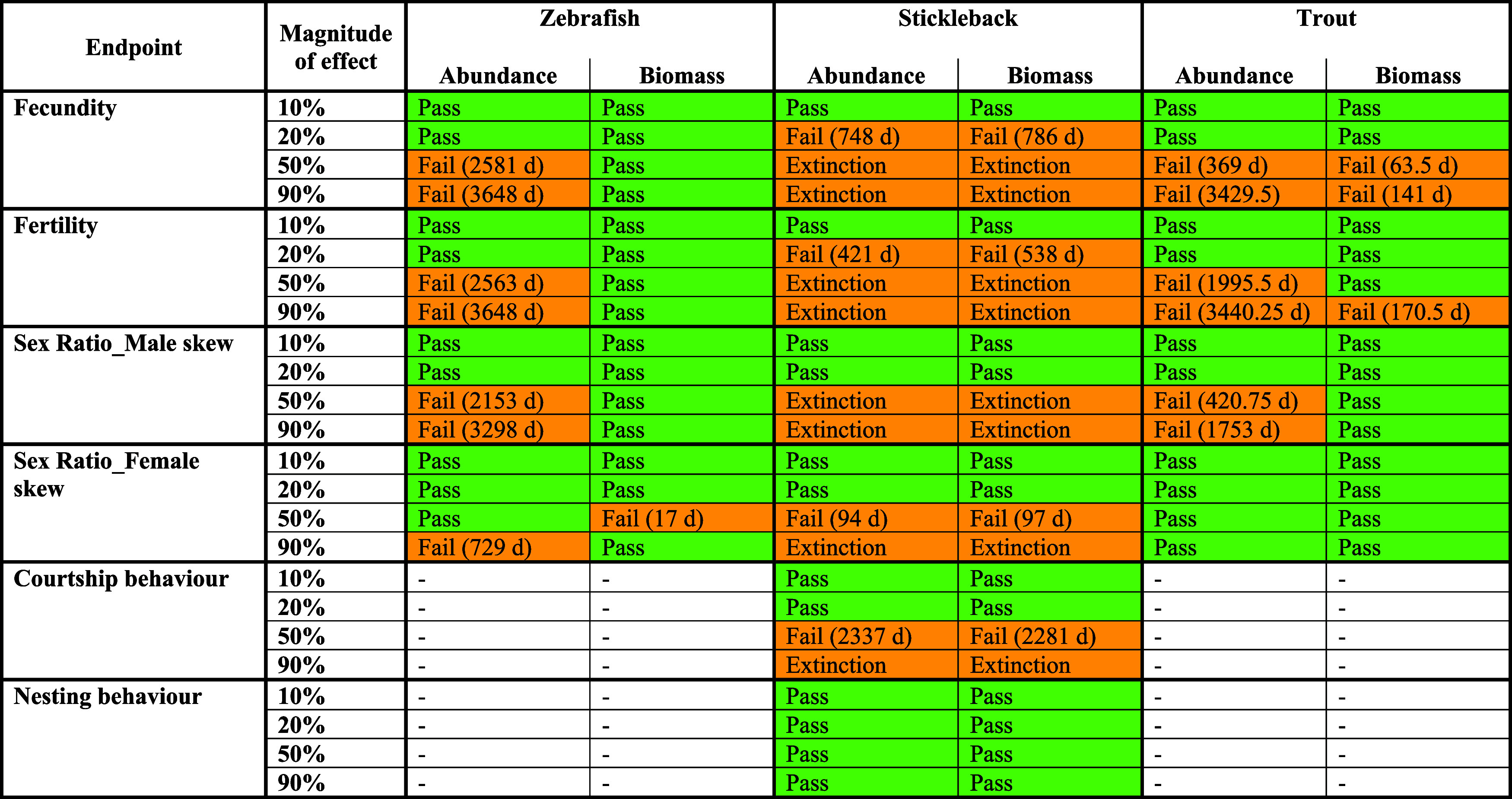
Population Results for Zebrafish, Stickleback, and Trout from Continuous 10-year Simulations of Individual-level Effects Indicating the Passing (Green) and Failing (Orange) Scenarios (Number of Days (D) Failing the Criterion) Considering EFSA^27^ Criterion 1 (The Exposed Population Mean Should Not Fall below the Lower 95th Percentile of the Control)

The population response differed between species, with the modeled zebrafish population (for which a 50% effect on an endpoint was necessary to incur any population response) more resistant to effects on all endpoints tested than the stickleback and trout populations. The stickleback was most sensitive to the imposed effects, showing population responses with a 20% reduction in fecundity and fertilization rate (Figure 3). Furthermore, at high effect magnitudes, the trout population persisted at lower abundances and biomass while the stickleback became extinct.

**Figure 3:**
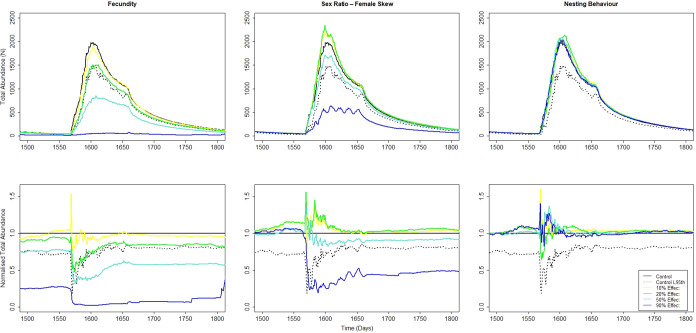
Stickleback total abundance across a single year of the 10-year treated period following endocrine-mediated adverse effects imposed on fecundity, sex ratio (female skew), and nesting behavior during the summer spawning season (top, absolute numbers; bottom, normalized against the control). The assessment criterion for a population-level effect relates to the mean of the treated population compared with the lower 95th percentile confidence limit of the control. For fecundity, sex ratio (female skew), and nesting behavior, this population effect is observed at 20%, 50%, and >90%, respectively. i.e., the 20% effect line in the fecundity graph at some point falls below the lower 95th percentile line from the control.

The simulations performed with shorter, yearly seasonal durations of imposed individual-level effects resulted in changes in population-level response to each endpoint and their magnitude (SI Table S5). If effects on reproductive endpoints were only imposed outside the spawning season for either the stickleback or the trout, then no population response was observed (e.g., effects imposed in Oct-Dec, but stickleback spawning is in May–July, and vice versa for trout). However, if effects on reproductive endpoints were only imposed inside the spawning season for either the stickleback or the trout, then population responses similar to those for the 10-year continuous exposure were observed (e.g., effects imposed in May–July and stickleback spawning in May–July).

## 4 Discussion

### 4.1 Difficulties in Applying the EFSA^27^ Criteria

The results indicate a number of critical issues with the second criterion from EFSA.^27^ Repeated control simulations fail the second criterion when they should not (see SI Table S4 and Figure 1), preventing the use of this criterion to differentiate actual changes in populations under different effect scenarios. Furthermore, variability in the NOR of a population may differ in response to exposure depending on biological and ecological interactions in the model and implementation of toxicity. Visual analysis of the outputs from the models presented here indicated that the population variability remained unchanged in the exposed simulations (due in part to how the effects were imposed in the model), meaning that any decrease in the population mean would lead to a decrease in the lower 95th percentile. In such a case, whether the decrease in the population mean resulted in a failure of criterion 1 would depend upon the magnitude of the decrease (i.e., small changes would still pass criterion 1, while large changes would result in a failure of criterion 1). However, any decrease in the mean, regardless of magnitude, would result in the automatic failure of criterion 2 for these models. Other cases from the wider literature indicate that this relationship of NOR variability following exposure may be even more fickle. For example, *Daphnia* populations exposed to Dispersogen A and *p353*-nonylphenol led to an increase in NOR variability,^38^ while *Daphnia* populations exposed to 3,4-dichloroaniline led to a decrease in NOR variability^39^ following chemical exposure. As such, a small change in the mean population following exposure to a chemical could lead to either a positive or a negative result in the second criterion. This criterion will likely fail when exposure leads to an increase in variability, even if the mean remains unchanged. On the other hand, the criterion may even tolerate a consistent reduction in the mean value if this coincides with a reduced variability in the exposed population. This dynamic is still poorly understood, and as such, further work is required in order to establish a second criterion that is scientifically robust and consistent with the level of protection offered by criterion 1 before being used to assess population model outcomes. It is essential that such an operational second criterion is devised, as the potential issue with solely using criterion 1 is that a population effect will be less likely if the NOR in the model is wide. For example, while the NOR in the models emerges from their parametrization and represents a realistic range for the population, the lower 95th percentile defining the NOR in the zebrafish model is roughly 15%. This means that detecting differences in the population less than this will not be picked up by criterion 1 alone.

Relatedly, throughout the modeling and analysis presented in this paper, it was assumed that if a single time step failed the assessment criterion, then a population response was observed. However, the EFSA^40^ opinion on Specific Protection Goals stated that “*seasons to rotations are relevant when the temporal scale of effects has its focus on long-term population dynamics, including risk of local extinction*”, in which case a single day where the criterion is “failed” should not be considered a population response. But what duration of population response should be deemed a population effect? For instance, the failure of the criterion in the 50% sex ratio—female skew in the 10-year simulation for stickleback was 94 and 97 days, for abundance and biomass, respectively (Table 1). This would meet the “seasons to rotations” and hence conclude a population-level effect. However, this is not the case in all scenarios. For example, the equivalent endpoint when modeled for stickleback in the 3-month (summer) simulation caused a failure of the criterion for only 5 days (abundance) (SI Table S5). As such, in order to use this modeling approach in a regulatory context, the temporal scale of the protection goal needs to be further defined.

### 4.2 Sensitivity to Different Endpoints and the Timing and Duration of Imposed Effects

Here we demonstrate how models can be used to assess the population relevance of changes in different individual-level endpoints observed in laboratory studies. In particular, we show that the (commonly used) simplifying assumption, that an effect and the magnitude of effect observed at the individual level in laboratory studies is the same as that at the population level, to be false *(see* ^41^ *)*. The AOP concept is currently used to describe the mechanistic progression from Molecular Initiating Event following exposure to an endocrine active substance to an adverse effect on an organism or a population.^22^ However, translation of effects observed in individuals to impacts on populations is made “*by extension*”.^22^ The modeling here focused on one part of this pathway, specifically the extrapolation from an adverse effect on individuals to an adverse effect on fish populations. Given the modeled scenarios and parametrizations, a 20% effect magnitude generally remains compatible with the maintenance of the modeled zebrafish and trout populations, while the modeled stickleback population is more sensitive to an effect on reproductive endpoints (fecundity and fertility) as a 20% effect magnitude on these endpoints impaired population abundance and biomass. Notably, no population effects were observed when any individual-level endpoint was altered by only 10% in isolation. However, once multiple endpoints were altered by 10% concurrently, then a population effect was observed. Due to its similarity to the accepted Effective Concentration estimate of 10% (EC_10_) derived from concentration–response relations, it may be tempting to apply a 10% magnitude for an adverse effect on individuals as a threshold for population-relevant effects (regardless of the apical endpoint evaluated). If protection of the population is the goal, then these model results indicate that a laboratory study showing an individual-level effect at 10% should only be considered population relevant if observed in multiple (in this case 3) endpoints concurrently, and a higher threshold at the individual level should be considered when evaluating toxicity studies for population relevance. Additionally, while a decreased population trajectory is intuitively expected when apical endpoints are negatively affected, the model revealed an increase in population biomass in the zebrafish for a number of endpoints (e.g., 50% reduction in fertilization rate). Meanwhile, on occasion, an increase in population abundance was observed in the trout (e.g., 50% sex ratio—female skew). This shows that the “population declining trajectory” assumption included by default in AOPs that include a population-level adverse outcome (e.g., AOPs 23, 25, and 30, see^24^) does not systematically hold true. While some field studies have shown population declines in some fish species (e.g., a multiyear lake study by Kidd et al.^42^ observed declines in fathead minnow following exposure to ethinylestradiol), other field observations have shown an absence of correlation between long-term exposure to estrogenic compounds, known to alter sex ratio and reproduction, and the density and self-sustainability of fish populations in nature *(see* ^43,44^ *)*. Nor is this relationship limited to sublethal effects of chemicals, with the phenomena known as the “hydra effect” covering situations where an increase in mortality actually leads to an increase in population size as a result of overcompensation.^45^ Simplistically, one might assume that an increase in population abundance or biomass is no cause for concern; however, knock-on effects in a community food web cannot be excluded but may be challenging to disentangle. Populations have a number of processes (e.g., density dependence) that may compensate for adverse effects on individuals and help regulate their total abundance^46^ and as such, modeling approaches such as that employed here should be a mainstay in chemical assessment to truly understand the impacts of chemical exposure on wildlife populations.

Our results show that populations were more sensitive to effects imposed at the individual level that directly altered recruitment, i.e., fecundity and fertilization rate. While they were less sensitive to effects imposed at the individual level that more indirectly altered recruitment, i.e., courtship behavior, sex ratio—male and female skew. For example, in the stickleback, a 20% reduction in fecundity meant that 20% fewer eggs were produced at each spawning, while a 20% disruption of courtship behavior meant that 20% of the female fish did not spawn each day, but they could try again the following day. As such, the effect on the number of eggs spawned across the spawning period was −20% for fecundity, but <−20% for disrupted courtship behavior (as the interspawn interval was multiple days). Furthermore, this clearly indicates that an apical endpoint and not a biomarker endpoint is essential to truly realize a population-level response assessment.

Recently there has been a growing interest in behavioral endpoints as EDCs have been shown to alter fish reproductive behaviors.^47^ Recent workshop outputs and reviews on behavior, more generally, have concluded that these behaviors are population relevant.^48,49^ However, our results indicate that this relationship is not so clear-cut as disruption of different behaviors at different magnitudes of disruption have different population relevance. Interestingly, while disrupted nesting behavior did not result in a stickleback population response, disrupted courtship did. This may be due to the ecological significance of these behaviors: in the former, a mature female simply mates with an alternate male who has successfully built their nest, while in the latter, she has already committed to an unsuccessful male, and her contribution to recruitment for the population is lost at that time point. Alternatively, as behavioral disruption was modeled randomly (so not associated with specific individuals), after 10 days at 90% nesting disruption, statistically speaking, all males would still be expected to have built a nest, so the impact on mating was reduced. This could be modeled differently depending on how the chemical-induced changes in nesting behavior (i.e., once a male is exposed and can no longer build a nest, he can never build a nest on any subsequent days either). This highlights the importance of the underlying assumptions made with any population model. Another example is the assumption of a 50:50 sex ratio in the control simulations for all three species, as this is not always the case in reality (i.e., the OECD^50^ Fish Sexual Development Test guideline validity criteria permit a control sex ratio of a maximum of 70:30 in either direction). Imposing a 50% further skew on the sex ratio (due to a chemical) when the control is 70:30 may well have greater or lesser population level implications than if starting with a population of 50:50. Regardless, our results do show that endpoints other than behavior may be more relevant for population-level assessment, making behavioral endpoints as currently evaluated in fish chronic tests relevant for endocrine disruption assessment of limited value to investigate the plausibility of the biological link between endocrine activity and population-relevant adverse effects.

The assessment of EDCs in the EU is hazard-based^18^ and as such, chemical exposure is not considered in any regulatory assessment. A number of critical decisions regarding how to implement effects in population models in a hazard-based EDC assessment context have been identified and include consideration of the duration of effects. Here, a worst-case (for agrochemicals) long-term (10-year) continuous duration of effects imposed on individuals had much the same population response as shorter effect durations (3 months per year) repeated for 10 years, as long as they were timed to coincide with the sensitive window. Hazlerigg et al.^13^ recommended a 1-year duration under the ECHA-EFSA^19^ guidance. While this 1-year recommendation implies a number of assumptions that may not be applicable to all chemicals, our results here indicate that population responses from a 3-month simulation (repeated each year, representative of annual use of pesticides on crops) could be expected to cover those for a longer duration up to 10 years. It is unclear whether the population response from a single year of imposed effects would be consistent with effects imposed for 3 months every year for 10 years, and requires further investigation.

### 4.3 Species Choice for Modeling Assessment

Species, model and scenario choice is an essential element of the modeling cycle whereby selection of vulnerable species is imperative to ensuring adequate environmental protection from chemical exposure.^34^ While stickleback and trout were selected for their potential vulnerability, the zebrafish (although less representative of aquatic ecosystems in European agricultural landscapes) was selected as an interesting comparator due to its different life history. The lower sensitivity of the population response to effects detected here for zebrafish suggests that its ecology and life-history traits make this species potentially less vulnerable than that of the stickleback and trout. As such, it provides further support that the stickleback and trout are more suitable focal species for population-level assessment and thereby appropriate for use in modeling approaches applied to the environmental safety assessment of EDCs. Individual-level responses identified in studies using common laboratory fish species (e.g., zebrafish, fathead minnow, medaka) can still be used in population modeling approaches in conjunction with suitable focal species.

Once focal species are identified, suitable models and scenarios are required. All three models in this study were taken “off-the-shelf”, i.e., the model and their ecological scenarios were taken from the original publications rather than specifically developed for use in an EDC assessment context. Since EFSA,^34^ there have been more recent efforts to prescribe how to define suitable environmental scenarios **(*see* ^51,52^ ). However, it is not yet clear how the environmental scenarios should be set for EDC assessments, as there are currently no agreed scenarios for such hazard-based assessments. When scenario development was under consideration for bees, they were defined as “*a representative combination of crop, soil, climate, and agronomic parameters to be used in modelling, representative means in this context that the selected scenarios should represent physical sites known to exist*”.^53^ The ecological setups in the three models used here are in keeping with this idea of representing physical sites. The trout model used a GIS input file with weather and hydrological time series for a specific creek. The stickleback model was based on ponds in England against which it was validated and similarly the zebrafish on ponds in Bangladesh. As such, in this project, we used the default environmental scenario developed by the original authors of each model, as each was developed against known field sites where the species were present and therefore reflect a real-life ecological system. However, these scenarios could have been setup differently, as the species are found in other areas under different environmental conditions. If these scenarios led to fish that were more, or less, “stressed”, then the responses of the fish populations subject to further effects from an endocrine active chemical may differ from those shown here for these species under this set of environmental conditions. This also extends to biological features of each model, with the zebrafish model, for example, assuming year-round reproduction due to the finding of gravid female fish in the wild by Spence et al.^54^ This is likely to have led to the higher resistance of the zebrafish modeled population to the effects imposed in this study. As such, should this assumption be investigated further and if found to be unsupported (e.g., while the potential for reproduction may be year−round, the reality may be that a lack of energy resources limits that reproduction), the population responses of the zebrafish may be more sensitive to smaller effect magnitudes. Equally, the stickleback model used in this project was extended to include an energy-budget component.^9^ If the energy flows in this more recent model altered the reproductive output of individual fish, once again, the population outcomes may change. This highlights the difficulty of establishing appropriate models and ecological scenarios, and we encourage the development of a library of models and scenarios for use in environmental safety assessment of chemicals, including EDCs, that are accepted by the scientific and regulatory community. In the meantime, we believe there is still a value in using a validated off-the-shelf model and scenario, combined with imposed effect magnitudes and evaluated against the first assessment criteria from EFSA^27^ when accompanied by a robust analysis of uncertainty. Once standardized, the “exposure-agnostic molecule-independent” approach presented here will enhance our understanding of population outcomes within the hazard assessment of EDCs.

## Supplementary Material





## Notes

Code and model log for stickleback, trout, and zebrafish models (Models.zip) (ZIP)
Model Parameterisation, Method Development, Additional results (SI_Final_Rev.doc) (PDF)
