# Supplementary material for: Assessment of Population Relevance of Endocrine-sensitive Apical Endpoints in Fish Chronic Studies Using Individual-Based Models
Source: Environ Sci Technol. 2025 Sep 30;59(40):21431–41. doi: 10.1021/acs.est.5c03364 (PMC12529950; doi:10.1021/acs.est.5c03364)
Supplement: Supplementary file 2 [file es5c03364_si_002.pdf]

Supporting Information to

Assessment of population relevance of endocrine-sensitive apical endpoints in fish chronic studies using individual-based models

Alice Tagliati[1], Charles R.E. Hazlerigg[1,2*], Edward R. Salinas[3], Laurent Lagadic[3], Thomas G. Preuss[3]

[1]Enviresearch Ltd., Newcastle-Upon-Tyne, NE1 4DP, UK

[2]School of Natural and Environmental Sciences, Newcastle University, Newcastle-upon-Tyne, NE1 7RU, UK

[3]Bayer AG R&D, Crop Science Division, Monheim am Rhein, 40789, Germany

Corresponding Author

* Charles.Hazlerigg@enviresearch.com

Total pages: 51

Contents

Table S1: Simulated endpoints for each species / model 3

Table S2: Effects parameters and magnitude of changes during simulations for each endpoint and model. 3

Table S3: Additional features in the GUI to implement the ED-mediated effects and their magnitude for each endpoint and model. 4

Table S4: Population results for pairwise comparisons of stickleback 10-year simulations with no effects imposed 6

Table S5: Population results for stickleback and trout from seasonal periods (3-months annually) 7

Table S6: Population results for combined effects in stickleback, trout and zebrafish from 10-year simulation 8

Model Logs 9

- Zebrafish 9
- Stickleback 16
- Trout 24

Comparison of replicate number 29

Figure S1: Mean (and 1.96 x Standard Error of the Mean) for total abundance in the control simulation for each population model with differing numbers of runs 30

Graphs for the 10-year of Continuous Effects 34

- Zebrafish 34
- Stickleback 37
- Trout 40

Graphs for the 10-years of Seasonal Effects 42

- Stickleback Summer 42
- Stickleback Winter 45
- Trout Summer 48
- Trout Winter 50

Graphs for the 10% combination of effects (fecundity, fertilisation rate, sex skew) 52

- Zebrafish 52
- Stickleback 53
- Trout 54

Table S1: Simulated endpoints for each species / model

Model	Species	Apical endpoints					
		Fecundity	Fertilization success	Sex ratio		Behaviour	
				male skewed	female skewed	Courtship	Nesting
inSTREAM	Brown trout (*Salmo trutta*)	●	●	●	●		
Zebrafish IBM	Zebrafish (*Danio rerio*)	●	●	●	●		
Stickleback IBM	Three-spined stickleback (*Gasterosteus aculeatus*)	●	●	●	●	●	●

Table S2: Effects parameters and magnitude of changes during simulations for each endpoint and model.

Endpoint	Parameter			Parameter value used in the control simulations	Parameter value used in the ED simulations (representing 10, 20, 50 and 90% magnitude of effect, respectively)
	Zebrafish Model	Stickleback Model	Trout Model		
**Sex Ratio – Male Skew**	*SexRatioMF* (slider in GUI) *	*SexRatio* (slider in GUI)	*Trout-SexRatio-Female* (new parameter)	0.5	0.45, 0.4, 0.25, 0.05
**Sex Ratio – Female Skew**	*SexRatioMF* (slider in GUI)	*SexRatio* (slider in GUI)	*Trout-SexRatio-Female* (new parameter)	0.5	0.55, 0.6, 0.75, 0.95
**Fecundity**	*Fec_Effect* (new parameter)	*Fec_Effect* (new parameter)	*Trout-Fecundity-Effect* (new parameter)	1	0.9, 0.5, 0.2, 0.1
**Fertilisation Rate**	*FertRate* (slider in GUI)	*fert* (parameter from original model)	*Trout-spawn-egg-viability* (parameter from original model)	0.88 (zebrafish)<br>0.935 (stickleback)<br>0.8 (trout)	79.2, 70.4, 44, 8.8<br>0.8415, 0.748, 0.4675, 0.0935<br>0.72, 0.64, 0.4, 0.08

**Courtship Behaviour**	-	*courtship-disruption* (parameter in original model)	-	0	0.1, 0.2, 0.5, 0.9
**Nesting Behaviour**	-	*nesting-disruption* (parameter in original model)	-	1	0.9, 0.8, 0.5, 0.1

ED Endocrine disruption * Modelling was performed in Netlogo, a slider has been added/adapted in the user interface that can be changed depending upon which simulation is desired

Table S3: Additional features in the GUI to implement the ED-mediated effects and their magnitude for each endpoint and model.

**Endpoint**	**Parameter**			**Parameter value used in the control simulations**	**Parameter value used in the ED simulations** (representing 10, 20, 50 and 90% magnitude of effect, respectively)
	**Zebrafish Model**	**Stickleback Model**	**Trout Model**		
**Sex Ratio – Male Skew**	*SR_ProportionMales* (new chooser in GUI) *	*Proportion_males* (new chooser in GUI)	*Toxicant-SexRatio-Effect* (new chooser in GUI)	1 (zebrafish and stickleback)<br>0.5 (trout)	0.9, 0.8, 0.5, 0.1<br><br>0.45, 0.4, 0.25, 0.05
**Sex Ratio – Female Skew**	*SR_ProportionMales* (new chooser in GUI)	*Proportion_males* (new chooser in GUI)	*Toxicant-SexRatio-Effect* (new chooser in GUI)	1 (zebrafish and stickleback)<br>0.5 (trout)	1.1, 1.2, 1.5, 1.9<br><br>0.55, 0.6, 0.75, 0.95
**Fecundity**	*Fecundity_Reduction* (new chooser in GUI)	*EggNumberReduction* (new chooser in GUI)	*Toxicant-Fecundity-Effect* (new chooser in GUI)	0 (zebrafish and stickleback)<br>1 (trout)	0.1 0.2 0.5 0.9<br><br>0.9 0.8 0.5 0.1
**Fertilisation Rate**	*Fertility_reduction* (new chooser in GUI)	*Fertilisation_effect* (new chooser in GUI)	*Toxicant-FertilisationRate-Effect* (new chooser in GUI)	0 (zebrafish and stickleback)<br>0.8 (trout)	0.1 0.2 0.5 0.9<br><br>0.72, 0.64, 0.4, 0.08
**Courtship Behaviour**	-	*Courtship_effect* (new chooser in GUI)	-	1	0.9, 0.8, 0.5, 0.1
**Nesting Behaviour**	-	*Nesting_effect* (new chooser in GUI)	-	0	0.1, 0.2, 0.5, 0.9

ED Endocrine disruption * Modelling was performed in Netlogo, a chooser has been added to the user interface that can be changed depending upon which simulation is desired

Table S4: Population results for pairwise comparisons of stickleback 10-year simulations with no effects imposed

Three sets of control simulations (Control 1, Control 2, Control 3) each with 70 runs were compared to each other, indicating the passing (green) and failing (orange) (number of days (d) failing the criterion) considering EFSA (2023) criterion 1 and criterion 2.

Comparison scenario	Criterion 1		Criterion 2	
	Abundance	Biomass	Abundance	Biomass
**Control 1 vs Control 2**	Pass	Pass	Fail (4500 d)	Fail (4459 d)
**Control 2 vs Control 1**	Pass	Pass	Fail (1222 d)	Fail (1303 d)
**Control 1 vs Control 3**	Pass	Pass	Fail (3544 d)	Fail (3115 d)
**Control 3 vs Control 1**	Pass	Pass	Fail (2130 d)	Fail (2647 d)
**Control 2 vs Control 3**	Pass	Pass	Fail (3544 d)	Fail (3115 d)
**Control 3 vs Control 2**	Pass	Pass	Fail (2130 d)	Fail (2647 d)

EFSA (2023) Criterion 1: the exposed population mean should not fall below the lower 95th percentile of the control and Criterion 2: the lower 95th percentile of the exposed population should not be consistently below the lower 95th percentile of the control population.

Table S5: Population results for stickleback and trout from seasonal periods (3-months annually)

Individual-level effects in either the winter (October – December, in breeding season for trout and outside breeding season for stickleback) or summer (May – July, vice versa), indicating the passing (green) and failing (orange) (number of days (d) failing the criterion) considering EFSA (2023) criterion 1.

Endpoint	Magnitude of effect	Stickleback				Trout			
		May-July		Oct-Dec		May-July		Oct-Dec	
		Abundance	Biomass	Abundance	Biomass	Abundance	Biomass	Abundance	Biomass
Fecundity	10%	Pass	Pass	Pass	Pass	Pass	Pass	Pass	Pass
	20%	Fail (866 d)	Fail (962 d)	Pass	Pass	Pass	Pass	Pass	Pass
	50%	Extinction	Extinction	Pass	Pass	Pass	Pass	Fail (911.75 d)	Pass
	90%	Extinction	Extinction	Pass	Pass	Pass	Pass	Fail (3434.5 d)	Fail (751 d)
Fertility	10%	Pass	Pass	Pass	Pass	Pass	Pass	Pass	Pass
	20%	Fail (275 d)	Fail (182 d)	Pass	Pass	Pass	Pass	Pass	Pass
	50%	Extinction	Extinction	Pass	Pass	Pass	Pass	Fail (778.75 d)	Pass
	90%	Extinction	Extinction	Pass	Pass	Pass	Pass	Fail (3432 d)	Fail (128.25)
Sex Ratio_Male skew	10%	Pass	Pass	Pass	Pass	Pass	Pass	Pass	Pass
	20%	Pass	Pass	Pass	Pass	Pass	Pass	Pass	Pass
	50%	Extinction	Extinction	Pass	Pass	Pass	Pass	Pass	Fail (2 d)
	90%	Extinction	Extinction	Pass	Pass	Pass	Pass	Pass	Fail (2 d)
Sex Ratio_Female skew	10%	Pass	Pass	Pass	Pass	Pass	Pass	Pass	Pass
	20%	Pass	Pass	Pass	Pass	Pass	Pass	Pass	Pass
	50%	Fail (5 d)	Pass	Pass	Pass	Pass	Pass	Pass	Pass
	90%	Extinction	Extinction	Pass	Pass	Pass	Pass	Pass	Pass
Courtship behaviour	10%	Pass	Pass	Pass	Pass	-	-	-	-
	20%	Pass	Pass	Pass	Pass	-	-	-	-
	50%	Extinction	Extinction	Pass	Pass	-	-	-	-
	90%	Extinction	Extinction	Pass	Pass	-	-	-	-
Nesting behaviour	10%	Pass	Pass	Pass	Pass	-	-	-	-
	20%	Pass	Pass	Pass	Pass	-	-	-	-
	50%	Pass	Pass	Pass	Pass	-	-	-	-
	90%	Pass	Pass	Pass	Pass	-	-	-	-

Table S6: Population results for combined effects in stickleback, trout and zebrafish from 10-year simulation

10% magnitude of effect imposed simultaneously on fecundity, fertilisation rate and sex ratio indicating the passing (green) and failing (orange) (number of days (d) failing the criterion) considering EFSA (2023) criterion 1.

Model	Endpoint	Abundance	Biomass
**Zebrafish**	**Female skew**	Pass	Pass
	**Male skew**	Pass	Pass
**Stickleback**	**Female skew**	Fail (281 d)	Fail (181 d)
	**Male skew**	Fail (1787 d)	Fail (1922 d)
**Trout**	**Female skew**	Pass	Pass
	**Male skew**	Fail (60.5 d)	Pass

Model Logs

Models and logs of their changes for each of the three fish models used in the study are presented. These relate to the simulations performed with ED-mediated effects imposed for a continual period of 10 years only.

Zebrafish

The original zebrafish model was the same as presented in Hazlerigg et al. (2014). The only exception is that the model has been updated to be compatible with a newer Netlogo version (v.6 compared with v.3 for the original model). This update included removal of coding for the graphical outputs (these are done automatically in user interface in v.6) and use of a few local variables in case of *?1* and *?2* when using lists in the sub-models. Any changes to the code during this update are highlighted in the v.6 code with the tag *";CH"* followed by either *"removal", "addition",* or *"change"* and the date "*XX/XX/XXXX*". The model used in the project is entitled: RA1561_Haz_2014_Mod_Fin_Rev_Trans_fin.nlogo

This model was then used as the basis for this project, with further updates made to address the specific aim of this project. These further updates are all highlighted in the v.6 code with the tag "*;AT*" followed by either *"Addition",* or *"Change"* and the date "*XX/XX/XXXX*". The details are presented below, but in general this involved the creation of choosers (in the user interface), new variables and sub-models to implement the hypothetical ED-mediated effects.

<table>
<tr><th>Section</th><th>Amendment</th></tr>
<tr><td>User Interface</td><td>Three new choosers added to left-hand side "Fecundity_Reduction", "SR_ProportionMales", and "Fertility_reduction" for the user to select the magnitude of ED effect (0, 10, 20, 50 and 90%) in each endpoint (egg production, sex ratio (male or female skew, depending on the selected value) and fertility reduction).

<table>
<tr><th>ED Effect</th><th>Chooser ID</th><th>Value</th><th>Magnitude of effect (%)</th></tr>
<tr><td>Fecundity</td><td>Fecundity_Reduction</td><td rowspan="2">0
0.1, 0.2, 0.5, 0.9</td><td rowspan="2">0 (Control)
10, 20, 50, 90</td></tr>
<tr><td>Fertilisation rate</td><td>Fertility_reduction</td></tr>
<tr><td>Sex Ratio</td><td>SR_ProportionMales</td><td>0.9, 0.8, 0.5, 0.1
1
1.1, 1.2, 1.5, 1.9</td><td>10, 20, 50, 90 - Male skew
0 (Control)
10, 20, 50, 90 - Female skew</td></tr>
</table>

Old sliders / features from the original model that are not relevant for this project have been moved to the bottom of the interface.</td></tr>
</table>

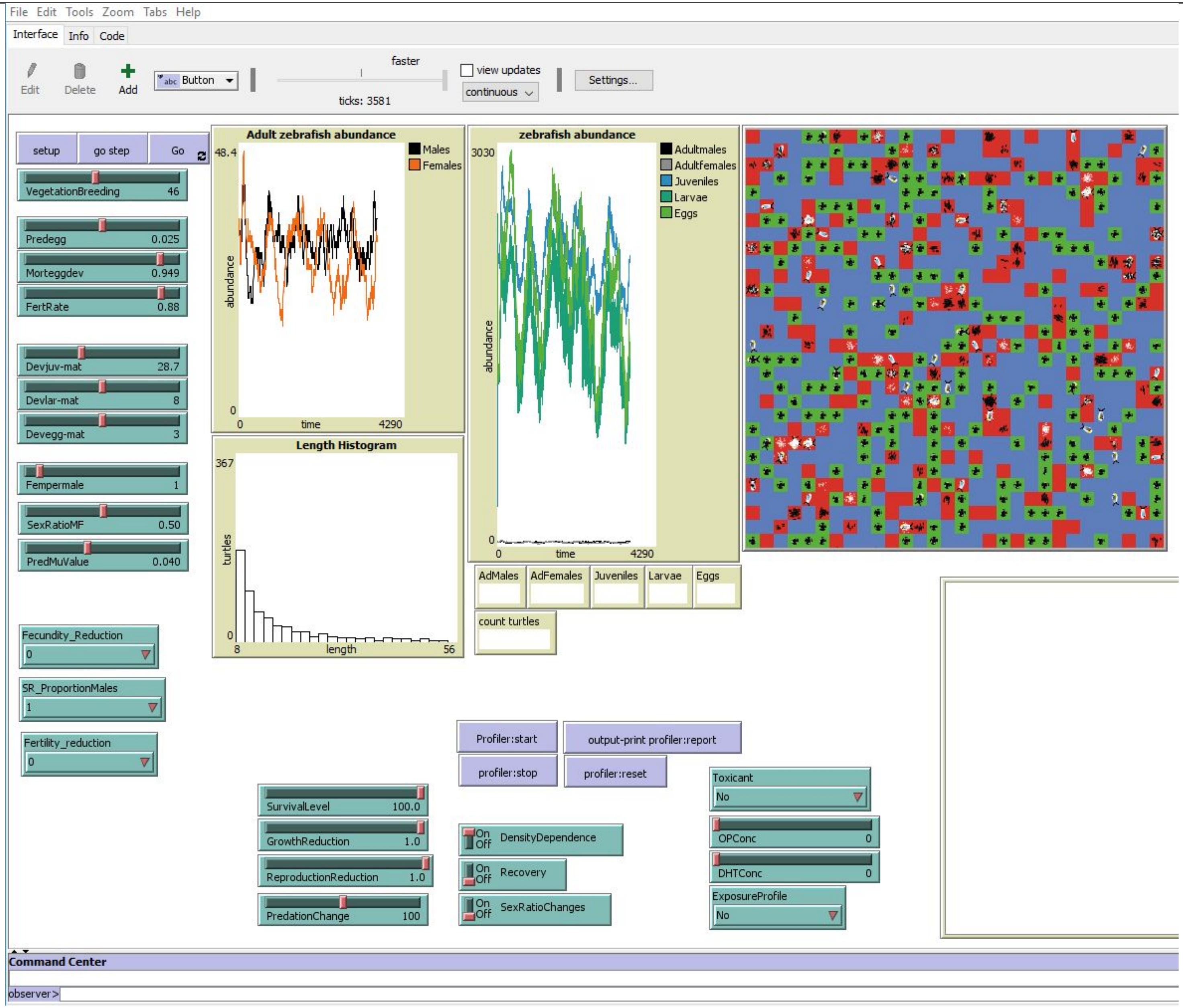
File Edit Tools Zoom Tabs Help
Interface Info Code
Edit Delete Add
Button
faster
view updates
continuous
Settings...
ticks: 3581
setup
go step
Go
VegetationBreeding 46
Predegg 0.025
Morteggdev 0.949
FertRate 0.88
Devjuv-mat 28.7
Devlar-mat 8
Devegg-mat 3
Fempermale 1
SexRatioMF 0.50
PredMuValue 0.040
Fecundity_Reduction
0
SR_ProportionMales
1
Fertility_reduction
0
Adult zebrafish abundance
Males
Females
48.4
abundance
0
0
time
4290
zebrafish abundance
Adultmales
Adultfemales
Juveniles
Larvae
Eggs
3030
abundance
0
0
time
4290
Length Histogram
367
turtles
0
8
length
56
AdMales
AdFemales
Juveniles
Larvae
Eggs
count turtles
Profiler:start
output-print profiler:report
profiler:stop
profiler:reset
SurvivalLevel 100.0
GrowthReduction 1.0
ReproductionReduction 1.0
PredationChange 100
On Off DensityDependence
On Off Recovery
On Off SexRatioChanges
Toxicant
No
OPConc 0
DHTConc 0
ExposureProfile
No
Command Center
observer>

New variable	Added new global variables used to implement effects on Fecundity (*Fec_Effect*) and Fertility (*Fert_constant*). No similar variable is needed for sex ratio as this can use what was already available in the model (*SRConstant*).

```
;create global variables including equation and world parameters
globals [ Rc
          Rd
          Rh
          Ri
          SurAdultacon
          SurAdultbcon
          PredMu
          Predb

          SRconstant

  ;AT Addition 11/11/2022 New variables are required to implement reduction in fecundity and fertility with exposure to an ED
          Fec_Effect
          Fert_constant
```

The values of the new variables are set in the setup sub-model (the starting values of 1 for *Fec_Effect* and 0.88 for *Fert_constant* are used, which will change in the modelling simulation):

```
to setup

  ;clear everything from the last simulation
  clear-all

  ;set values for global variables
  set Rc 0.884448688
  set Rd -2.70443978
  set Rh -0.000133
  set Ri 0.0041722
  set SurAdultacon 0.0000532662
  set SurAdultbcon 0.002649327
  set PredMu PredMuValue ;0.060
  set Predb -0.382

  set SRconstant SexRatioMF

  ;AT Addition 11/11/22 Default value for new variable for imposing effect of ED exposure on fecundity and fertility
  ;AT Addition 11/11/22 Cont.  (default values of 1 for fecundity and growth and 0.88 for fertility will change in simulations)
  set Fec_Effect 1
  set Fert_constant FertRate
```

"*SexRatioMF*" (available in the original model to set the sex ratio of zebrafish young) and "*FertRate*" default values are set in the BehaviorSpace settings:

	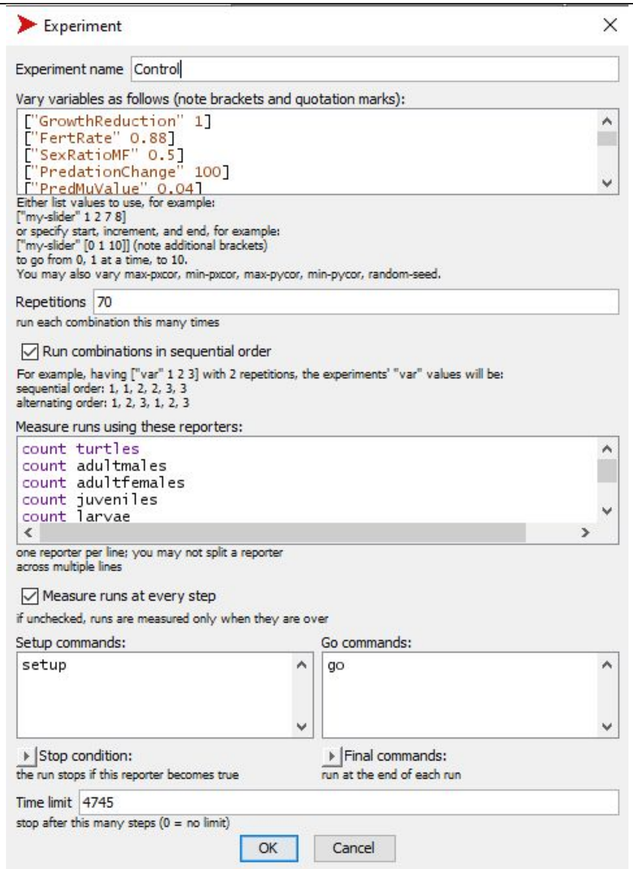
To toxicant effect sub-model	The toxicant effect sub-model is replaced with one relevant for this project. Specifically, after a 3 years spin-up period the user-selected magnitude of effect on sex ratio (either Male or Female skew), fecundity and fertility are implemented. The effects will be applied every day from then on until simulation completion.


```
;toxicant effects
;AT Change 11/11/22 Removed the toxicant effect from the original model and replaced with that specific for this project.
;AT Change 11/11/22 Cont. Specifically, after 3 years spin-up, imposing effects on sex ratio (either male or Female skew),
;AT Change 11/11/22 Cont. fecundity and fertility based on the values in the choosers in the user interface
to toxicant-effect

if ticks = 1095
  [ set SRConstant ( SRConstant * SR_ProportionMales )                          ;Sex Ratio Male skewed (0.1, 0.2, 0.5, 0.9) and Female skewed (1.1, 1.2, 1.5, 1.9); Control = 1
    set Fec_Effect ( 1 - Fecundity_Reduction )                                  ;Fecundity effect
    set Fert_constant ( Fert_constant - ( Fert_constant * Fertility_Reduction))  ;Fertility effect
  ]
```

<table>
<tr>
<td>To develop sub-model</td>
<td>Development sub-model is updated to remove effects of toxicant from the original model and impose those relevant to this project. Specifically, altering sex ratio for developing fish when exposed to the hypothetical ED:

```
to develop

  ;all fish increase age by 1 day each time-step
  ask turtles [ set age ( age + 1 ) ]

  ;AT Change 11/11/22 Removed original toxicity effects and replaced with those for this project on sex ratio.
  ;AT Change 11/11/22 Cont. Specifically, based on the slider and toxic effect sub-model, the SRConstant will be set different to the default of 0.5
  ;AT Change 11/11/22 Cont. resulting in less (Male skew) or more (Female skew) females.

  ;if larvae > development age (dpf) then become juveniles
  ;set sex to male or female from SRconstant
  ask larvae [ if age >= Devlar-mat
             [ set breed juveniles
               set size 0.5
               if sex = "undifferentiated"
                 [ ifelse random-float 1 < SRconstant
                     [ set sex "female" ]
                     [ set sex "male" ]
  ;               [ ifelse Toxicant = "4-tert-octylphenol"
  ;                 [ ifelse random-float 1 < (1 - SRconstant)
  ;                   [ set sex "male" ]
  ;                   [ set sex "female" ]
  ;                 ]
  ;                 [ ifelse Toxicant = "Dihydrotestosterone"
  ;                   [ ifelse random-float 1 < (1 - SRconstant)
  ;                     [ set sex "female" ]
  ;                     [ set sex "male" ]
  ;                   ]
  ;                   [ ifelse random-float 1 < SRconstant
  ;                     [ set sex "male" ]
  ;                     [ set sex "female" ]
  ;                   ]
  ;                 ]
                 ]
               ]
             ]

  ;if juveniles > development size (L) then become adults
```
</td>
</tr>
<tr>
<td>To reproduce sub-model</td>
<td>Altered reproduction sub-model to include a factor imposing the fecundity and fertility effects of the hypothetical ED – reducing egg production and fertilisation rate when ED effects are included:</td>
</tr>
</table>

	```<br>to reproduce<br><br>;AT Change 11/11/22 Fecundity and Fertility are reduced based on the ED effects calculated in the toxicity sub-model<br>;AT Change 11/11/22 Cont. Specifically, ED exposure leads to a Fert_constant < 0.88 and a Fec-Effect <1<br><br>;Adultfemales on breeding grounds with adultmales here produce eggs,<br>;egg number dependent upon adultfemale size and fertilisation rate<br>;Reset interspawning interval based upon age<br>ask adultfemales [if [habitat-type] of patch-here = "breeding-ground" and any? adultmales-here<br>; [hatch-eggs (((Reproa * (fishlength ^ Reprob)) * FertRate) * ReproductionReduction)<br>[hatch-eggs (((Reproa * (fishlength ^ Reprob)) * Fert_constant) * Fec_Effect)<br>[setxy [xcor] of self + random-normal 0 0.15 [ycor] of self + random-normal 0 0.15<br>set fishlength 0.7<br>set fishweight 0.2<br>set age 0<br>set size 0.3<br>set color white<br>set sex "undifferentiated"]<br>]<br>]<br>```

Stickleback

The original stickleback model was received from Katie Mintram – Supplementary information NetLogo model – from Mintram et al. (2018). The amended model used in this project is renamed *Mintram_2018_RA1561.nlogo* and includes the original model with the changes outlined in this document.
All changes in the model code have been identified with "*;AT*" or "*;CH*" and the date "*XX/XX/XXXX*". The stickleback model code changes for this project are the following:

<table>
<tr><td>Section</td><td>Amendment</td></tr>
<tr><td>User Interface</td><td>
Five new choosers added to left-hand side "EggNumberReduction", "Fertilisation_effect", "Proportion_males", "Courtship_effect" and "Nesting_effect" for the user to select the magnitude of ED effect (0, 10, 20, 50 and 90%) in each endpoint (i.e. egg production, fertilisation rate reduction, sex ratio (male or female skewed, depending on the value selected), courtship and nesting disruption).
<table>
<tr><th>ED Effect</th><th>Chooser ID</th><th>Value</th><th>Magnitude of effect (%)</th></tr>
<tr><td>Fecundity</td><td>EggNumberReduction</td><td rowspan="3">0
0.1, 0.2, 0.5, 0.9</td><td rowspan="4">0 (Control)
10, 20, 50, 90</td></tr>
<tr><td>Fertilisation rate</td><td>Fertilisation_effect</td></tr>
<tr><td>Nesting behaviour</td><td>Nesting_effect</td></tr>
<tr><td>Courtship behaviour</td><td>Courtship_effect</td><td>1
0.9, 0.8, 0.5, 0.1</td></tr>
<tr><td>Sex Ratio</td><td>Proportion_males</td><td>0.9, 0.8, 0.5, 0.1
1
1.1, 1.2, 1.5, 1.9</td><td>10, 20, 50, 90 (Male skew, i.e. SexRatio: 0.45, 0.4, 0.25, 0.05)
0 (Control, i.e. SexRatio: 0.5)
10, 20, 50, 90 (Female skew, i.e. SexRatio: 0.55, 0.6, 0.75, 0.95)</td></tr>
</table>
The "Proportion_Males" slider from the original model is renamed "SexRatio" and sets the default sex ratio of 0.5 at the start of each simulation before the ED-mediated effects are imposed.

Old sliders / features from the original model that are not relevant for this project have been moved to the right side of the interface.
</td></tr>
</table>

<table>
<tr><td></td><td>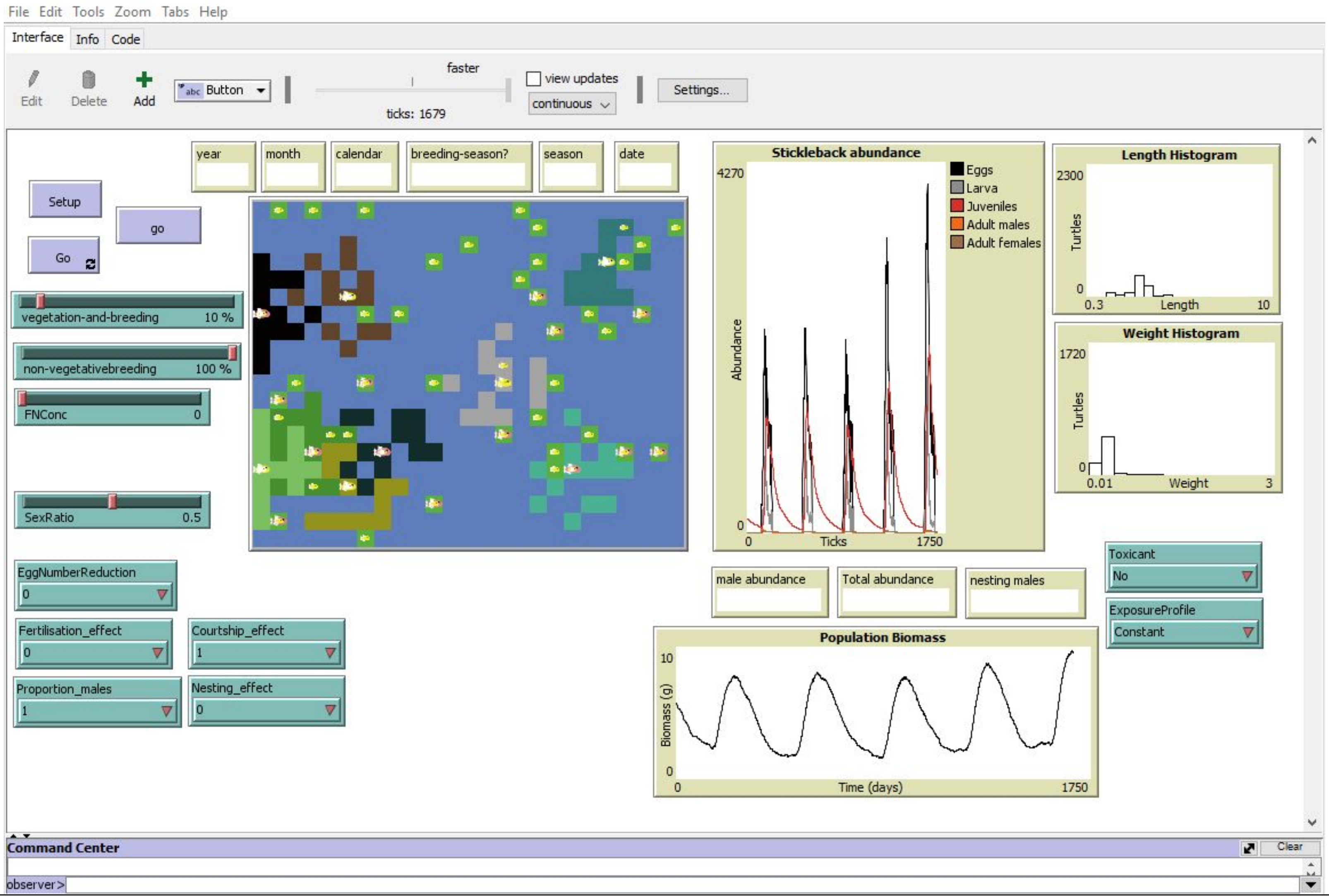
</td></tr>
<tr><td>New variables</td><td>Added new global variable used to implement effects on Fecundity (Fec_Effect). A variable for fertility is already available in the model, named “fert”.

The original toxicant effect parameters from Mintram et al. 2018 were also switched off using a “;”</td></tr>
</table>

```
; ## Toxicant effect parameter
; ToxicantConcentration                    ; microgram/ l
; FN-courta                                ; logit dose-response equation parameters for leading behaviour
; FN-courtb
; FN-nesta                                 ; logit dose-response equation parameters for nest building
; FN-nestb


;AT 17/11/2022: Add globals for implementing the effects of ED exposure
Fec_Effect         ;Fecundity effects
```

The values of the new variables are set in the setup sub-model (the default value of 1 for Fecundity is used, which will change in the modelling simulation):

```
; set FN-courta -0.42
; set FN-courtb 0.40
; set FN-nesta -1.01
; set FN-nestb 0.36

 ;AT 17/11/2022: Addition to set the default values of 1 to Fec-effect variable
 set Fec_Effect 1
```

Also, sex ratio is already available as slider in the model interface, but its name is changed from "*Proportion_males*" to "*SexRatio*" and so the code in the "to setup-turtle" submodel was amended accordingly:

```
to setup-turtles
  create-juveniles 160
  [
    set shape "fish"
    set color yellow
    set size 0.5
    set age 142 + random 90       ; initial age between 142 and 232 dph - based on a max hatch date of 8 days, a breeding season between May and July
                                  ; final spawnings 1 day after the breeding season (males establishing nests on the final of season) and the first spawnings 1 day after the beginning of the season.
    ; setting fishlength accoridng to eq 3: age in the equation is in years so needs to be divided by 360. The sin function in netlogo returns the angle rather than the raidion (angle of 2pi = 360)
    set fishlength (Linf * ( 1 - exp ( - ( ( K * ( ( age / 360 ) - t0 ) + (( c * K ) / ( 2 * pi )) * sin ( 360 * ( age / 360 ) - ts ) - (( c * K ) / ( 2 * pi )) * sin ( 360 * t0 - ts )))))  ; eq 3
    set fishweight (weight_a * ( fishlength ^ weight_b ) ) ; eq. 4
    set biomass_density (sum [fishweight] of turtles with [ breed != egg-batches and breed != larvae ] / pond_area)

    ;AT 17/11/2022: Code amended to apply the change of the slider name from "Proportion_males" to "SexRatio".
    ;  ifelse random-float 1 < Proportion_males ; sex ratio is set to 0.5 (slider)
    ifelse random-float 1 < SexRatio   ; sex ratio is set to 0.5 (slider)
      [set sex "male"]
      [set sex "female"]

    ifelse any? patches with [ habitat-type = "vegetation"]        ; juveniles will move to vegetative habitat if any are available
      [ move-to one-of patches with [ (habitat-type = "vegetation")]]
      [ setxy random-xcor random-ycor ]
  ]

end
```

"*SexRatio*" default value of 0.5 is set either from the chooser in the user interface or in BehaviorSpace setting:

	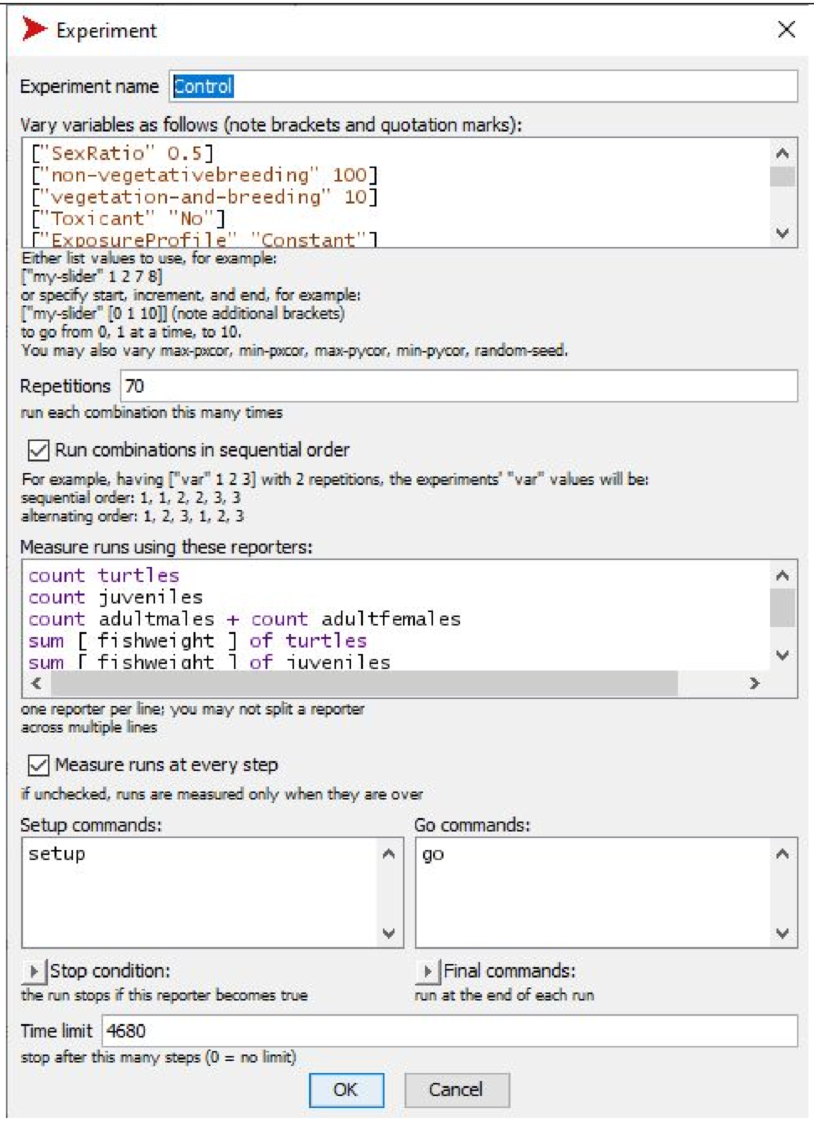
To toxicant effect sub-model	The toxicant effect sub-model is replaced with one relevant for this project. Specifically, after a 3 years spin-up period (i.e. 1080 ticks) the user-selected magnitude of effect on sex ratio (”*Proportion_males*”), fecundity (”*EggNumberReduction*”), fertility (”*Fertilisation_effect*”), nesting behaviour (”*Nesting_effect*”) and courtship behaviour (”*Courtship_effect*”), are implemented.<br>The effects will be applied every day from then on until simulation completion.

```
to toxicant-effect

  ;AT 17/11/2022: removed the toxicant effect from the original model and replaced with that specific for this project.
  ;AT 17/11/2022 Cont. Specifically, after 3 years spin up, effects are imposed on sex ratio, fecundity, fertility,
  ;AT 17/11/2022 Cont. nesting and courtship behaviours, based on the values in the choosers in the user interface.
  ;AT 17/11/2022 Cont. Note for courtship disruption 1 is no disruption and 0 is maximum disruption
 if ticks = 1080
  [ set SexRatio ( SexRatio * Proportion_males )             ;Sex Ratio Female skewed
    set Fec_Effect ( 1 - EggNumberReduction )                ;Fecundity effect
    set fert (fert * (1 - Fertilisation_effect))             ;Fertilisation rate effect
  ]

if ticks >= 1080 and ticks <= 4680
    [ask adultmales
      [set nesting-disruption Nesting_effect             ;Nesting behaviour effect
       set courtship-disruption Courtship_effect         ;Courtship behaviour effect
      ]
    ]


;  if ticks > 3600 and ticks < 7200 ; allows 10 years to stabilise
;  [
;    set ToxicantConcentration FNConc  ; toxicant conc set according to slider
;    ask adultmales
;    [
;      ifelse toxicant = "Yes" and ToxicantConcentration > 0
;      [
;        ifelse ExposureProfile = "Constant"
;        [
;          set courtship-disruption ( 1 / (1 + exp ( - (FN-courta + ( ( ln ToxicantConcentration ) * FN-courtb ) ) ) ) )
;          set nesting-disruption ( 1 / (1 + exp ( - (FN-nesta + ( ( ln ToxicantConcentration ) * FN-nestb ) ) ) ) )
;        ]
;        [
;          ifelse date >= 160 and date <= 170
;          [
;            set courtship-disruption ( 1 / (1 + exp ( - (FN-courta + ( ( ln ToxicantConcentration ) * FN-courtb ) ) ) ) )
;            set nesting-disruption ( 1 / (1 + exp ( - (FN-nesta + ( ( ln ToxicantConcentration ) * FN-nestb ) ) ) ) )
;          ]
;          [
;            set courtship-disruption 0
;            set nesting-disruption 0
;          ]
;        ]
;      ]
;      [
;        set courtship-disruption 0
;        set nesting-disruption 0
;      ]
;    ]
;  ]

end
```

To develop sub-model	Development sub-model is updated to impose courtship and nesting effects as well as sex ratio effects for developing fish:

```
to develop
 ; juveniles develop into adult males or females at the onset of the breeding season
   ;CH 27/11/2023: "courtship-disruption" and "nesting-disruption" addition to ensure future generations impose correct disruption to courtship and nesting
  if breeding-season? = "Yes"
    [
      ask juveniles with [ (sex = "male") and (age >= 235 )]  ; age ensures juveniles spawned in this breeding season do not become adults
      [
        set breed adultmales
        set shape "fish"
        set color blue
        set size 1
        setxy random-xcor random-ycor
        set nested? false
        set begin-nesting random 30 + 120
        set end-nesting 210 ; all nesting ceases on July 30th
        set courtship-disruption 1 ;CH 27/11/2023 Addition to ensure future generations impose correct disruption to courtship
        set nesting-disruption 0 ;CH 27/11/2023 Addition to ensure future generations impose correct disruption to nesting
      ]

      ask juveniles with [sex = "female" and (age >= 235 )]
      [
        set breed adultfemales
        set shape "fish"
        set color pink
        set size 1
        setxy random-xcor random-ycor
        set ready-to-spawn random 30 + 120
        set end-spawning 210
      ]
    ]
 ; eggs take 6 - 8 days to hatch into larvae
  ask egg-batches
    [
      if egg-age > 5 + random 3
      [
        hatch-larvae EggNumber
         [
          set shape "fish"
          set size 0.5
          set sex "n/a"
          set age 0
          set fishlength length-at-hatch
         ]
        die
      ]
    ]
```

	```<br>; larvae take 4 days to develop into independently feeding juvenile<br>;AT 17/11/2022: Code for SexRatio changed to implement ED effect<br>;AT 17/11/2022 Cont. Specifically, based on the slider and toxic effect sub-model, SexRatio will be set more or less than the default of 0.5 resulting in less or more females, respectively.<br>ask larvae<br>[<br>if age >= 4<br>[<br>set breed juveniles<br>set shape "fish"<br>set color yellow<br>set size 0.5<br>ifelse random-float 1 < SexRatio ; sex ratio is set to 0.5 (slider)<br>[set sex "female"]<br>[set sex "male"]<br>; [set sex "male"]<br>; [set sex "female"]<br>]<br>]<br>end<br>```
To reproduce sub-model	The variables "*nesting-disruption*" and "*courtship-disruption*" were already implemented in the reproduce sub-model of the original model. However, the "*courtship success*" equation has been altered to consider that effects to be investigated in this project are relative not absolute.<br>```<br>ifelse any? primary-nest-sites<br>[<br>move-to one-of primary-nest-sites<br>set nested? true<br>; eq. 6, courtship success as a function of terr size<br>; courtship-disruption is only greater than 0 if toxicant = "Yes"<br><br>;CH 27/11/2023 changed the "- (courtship-disruption)" to "* courtship-disruption" as effects to be investigated in this project are relative not absolute<br>;set courtshipsuccess (csuccessa * ln (territory-size) + csuccessb) - (courtship-disruption)<br>set courtshipsuccess (csuccessa * ln (territory-size) + csuccessb) * (courtship-disruption)<br>;print courtshipsuccess CH 27/11/2023 used to check implementation of courtship-disruption<br><br>ifelse any? secondary-nest-sites<br>[<br>move-to one-of secondary-nest-sites<br>set nested? true<br>;set courtshipsuccess (csuccessa * ln (territory-size) + csuccessb) - (courtship-disruption)<br><br>;CH 27/11/2023 changed the "- (courtship-disruption)" to "* courtship-disruption" as effects to be investigated in this project are relative not absolute<br>set courtshipsuccess (csuccessa * ln (territory-size) + csuccessb) * (courtship-disruption)<br>;print courtshipsuccess CH 27/11/2023 used to check implementation of courtship-disruption<br>```
To spawn sub-model	The spawn sub-model is altered to add the Fecundity effects. Effects on fertilisation rate are implemented by using the already available "fert" variable.

```
;AT 17/11/2022: "Fec_effect" variable is added to the original "EggNumber" equation to implement ED related fecundity effects.
;AT 17/11/2022 Cont. Effects altering fertility rate are implemented by using the "fert" variable already in the equation.
 ask adultfemales
   [ if (day_of_ISI >= interspawninterval ) and count adultmales-here with [ day_of_breedingcycle >= 1
     and day_of_breedingcycle <= 4 ] = 1 and (females-spawnedhere < 5) and (random-float 1 < fem_courting_prob )
     [
       set day_of_ISI 0
       hatch-egg-batches 1
       [
         set shape "dot"
         set size 0.5
         set age 0
         set sex "n/a"
         set color white
         ; set EggNumber ( feca * ( fishlength ^ fecb) ) * fert ; ( eq.7)
         set EggNumber ((( feca * ( fishlength ^ fecb) ) * fert) * Fec_Effect)
         set fishweight "n/a"
         set fishlength "n/a"
        ; adjusting the ts parameter from the seasonal growth algorithm so that all fish stop/start growing seasonally at the same time
         set ts_adjusted ts + ((165 - date) * 0.5)
       ]
       ask patch-here
         [ set females-spawnedhere (females-spawnedhere + 1) ]
     ]
   ]
```

Trout

This trout model is named inSTREAM and is now available for download on the developers website: [InSTREAM 7 and InSALMO 7 | Cal Poly Humboldt](). The version of inSTREAM used in this project was an earlier version that was provided directly by Steve Railsback. The amended model used in this project is renamed *inSTREAM7_2020_08_07_RA1561.nlogo* and includes the original model with the changes outlined in this document. The details are presented below, but in general this involved the creation of choosers (user interface), new variables and sub-models to implement hypothetical ED-mediated effects. All changes in the model code have been identified with "*;AT*" or "*;CH*" and the date "*XX/XX/XXXX*".

<table>
<tr><td>Section</td><td>Amendment</td></tr>
<tr><td>User Interface</td><td>Three new choosers added to left-hand side "Toxicant-SexRatio-Effect", "Toxicant-Fecundity-Effect" and "Toxicant-FertilisationRate-Effect" for the user to select the magnitude of ED effect (0, 10, 20, 50 and 90%) in each endpoint (sex ratio (male or female skew, depending on the selected value), egg production and fertility).
<table>
<tr><th>ED Effect</th><th>Chooser ID</th><th>Value</th><th>Magnitude of effect (%)</th></tr>
<tr><td>Sex Ratio</td><td>Toxicant-SexRatio-Effect</td><td>0.45, 0.4, 0.25, 0.05 = Male Skew
0.5 = Control
0.55, 0.6, 0.75, 0.95 = Female skew</td><td rowspan="3">0 (Control)
10, 20, 50, 90</td></tr>
<tr><td>Fecundity</td><td>Toxicant-Fecundity-Effect</td><td>1 = Control
0.9, 0.8, 0.5, 0.1</td></tr>
<tr><td>Fertilisation Rate</td><td>Toxicant-FertilisationRate-Effect</td><td>0.8 = Control
0.72, 0.64, 0.4, 0.08</td></tr>
</table>
</td></tr>
</table>

	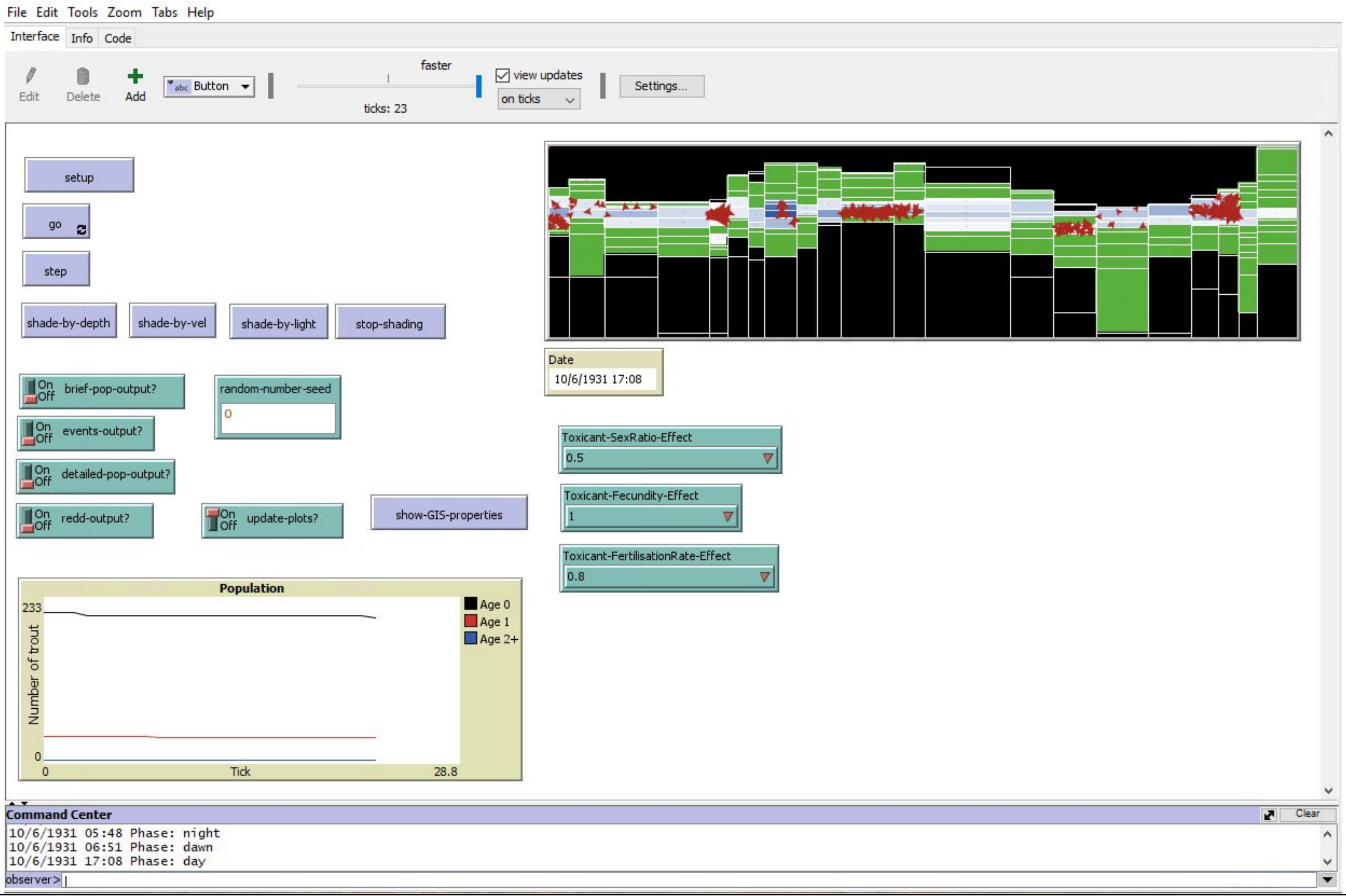
New .txt file with inputs	A new version of the input file was prepared for this project and called at the start of the model code:<br>`;__includes [ "parameters.nls" "test-procedures.nls" ]`<br>`__includes [ "parameters-LJC-LowerMainstem_AT.nls" ]`
New variable	Added new global variables used to implement effects on Sex Ratio (*Trout-SexRatio-Female*) and Fecundity (*Trout-Fecundity-Effect*). No similar variable is needed for fertilisation rate as this can use what was already available in the model (*trout-spawn-egg-viability*).

<table>
<tr><td></td><td>

```
;AT 06/12/2022 New variables for implementing the effects of ED exposure

  Trout-SexRatio-Female        ; AT addition 06/12/2022 original version of inSTREAM used - one-of ["male", "female"] - so need a new variable to determine sex which can then be changed if the toxicant is present
  Trout-Fecundity-Effect       ; AT addition 06/12/2022 trout variable to implement toxicant effects on fecundity (i.e. number of eggs produced) of fish
```

The values of the new variables are set in the additional text input file “parameters-LJC-LowerMainstem_AT”; in addition the value of the variable “trout-spawn-egg-viability” is defined as a specific value (0.8) instead of being a list of the single value 0.8.

```
; AT 06/12/2022 New variables default value
set Trout-SexRatio-Female        (0.5 )    ; CH Addition 04/08/2022 The proportion of new trout that are female, which is altered when exposed to toxicant
set Trout-Fecundity-Effect       (1)       ; AT Addition 06/12/2022 Trout variable which is reduced when exposed to toxicant resulting in a less number of egg produced


;AT amendment 06/12/2022. Variable "trout-spawn-egg-viability" is defined as the specific value 0.8 instead of being a list of  the single value 0.8.
set trout-spawn-egg-viability     (0.8)
;  set trout-spawn-egg-viability     (list    0.8    )
```

</td></tr>
<tr><td>To update-toxicant sub-model</td><td>

A toxicant effect sub-model is added to the “to go” procedure.

```
to go
  ; The main model schedule

  ; Determine if it is time to stop.
  ; This is first so that BehaviorSpace reports correct time when
  ; reporting results only at end of simulation instead of every tick.
  if time:is-after? sim-time sim-end-time
  [
    show (word "Simulation finished in " timer " seconds.")
    stop
  ]

  tick
  if ticks = 1 [ reset-timer ]

  ; Advance the date and time and update time-series habitat variables.
  update-time-and-habitat

  ; Update trout variables
  update-trout

  update-logistics


;AT addition 06/12/2022 - Implements ED effects
  update-toxicant
```

And a new “to update-toxicant” is created. Specifically, after a 3-year spin-up period the user-selected magnitude of effect on the endpoints is implemented. The effects will be applied every day from then on until simulation completion.
(3 years warm up period: 1 leap year + 2 normal years = 1096 days * 4 timesteps = 4384 ticks)

</td></tr>
</table>

```
to update-toxicant
  ;AT addition 06/12/2022
  ;Update the effects of the chemical exposure on trout every timestep after 3 years of warm up period (4384 ticks)
  ;If no effects are implemented, the trout parameters are set to default (un-exposed) values

  ask turtles [if ticks = 4384
              [ set Trout-SexRatio-Female Toxicant-SexRatio-Effect
                set trout-spawn-egg-viability Toxicant-FertilisationRate-Effect
                set Trout-Fecundity-Effect Toxicant-Fecundity-Effect
  ]]
```

The magnitude of effects that are implemented in the model are set in BehaviourSpace at the start of the simulation:

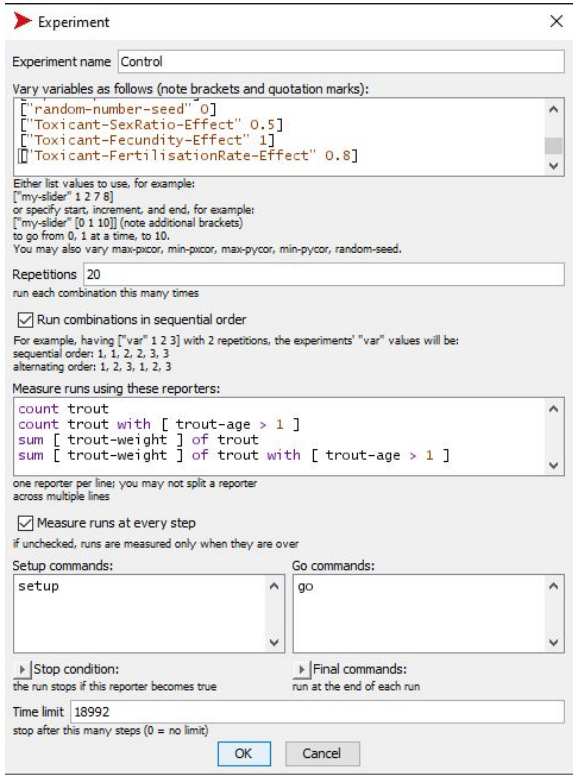

To initialize-trout-with sub-model	*Initialize-trout-with* sub-model was updated to use the new parameter (*Trout-SexRatio-Female*) for determining sex of new fish.<br><pre>to initialize-trout-with [a-species an-age a-length a-superind-rep]<br>; Trout reporter to initialize a newly created trout. Used for both model intialization<br>; and emergence of new trout from redds<br><br>; Set state variables<br>set trout-species a-species<br>set trout-spp-index position a-species species-list<br>set trout-age an-age<br>;AT amendement 06/12/2022 - changed previous code to now include the variable "Trout-SexRatio-Female" when determining sex<br>;AT cont. in order to allow potential chemical effects on sex ratio to be implemented in the exposure simulations<br>ifelse random-float 1 < Trout-SexRatio-Female<br>[set trout-sex "female"]<br>[set trout-sex "male"]<br><br>set trout-length (a-length)<br>set trout-condition 1.0<br>set trout-weight trout-condition * ((item trout-spp-index trout-weight-A) * (trout-length ^ (item trout-spp-index trout-weight-B)))<br>set trout-superind-rep a-superind-rep<br>set trout-spawned-this-season? false<br>set why-not-spawn "male" ; This will be changed when females try to spawn<br>set trout-tracked? (random-float 1.0 < frac-trout-to-output) ; This reports TRUE or FALSE</pre>
To num-viable-eggs sub-model	The *num-viable-eggs* sub-model is altered to include fertility and fecundity effects of the hypothetical ED. To implement fertility effects, the original variable "*trout-spawn-egg-viability*" is used. To implement fecundity effects, an additional factor ("*Trout-Fecundity-Effect*") was included:<br><pre>to-report num-viable-eggs<br>; A trout procedure to calculate the number of viable eggs a female lays<br><br>;AT amendment 06/12/2022 fecundity effect added to the equation which set the number of viable eggs in the redd. Control value of 1 is modified during simulation.<br><br>report round (((item trout-spp-index trout-spawn-fecund-mult) *<br>(trout-length ^ (item trout-spp-index trout-spawn-fecund-exp)) *<br>(trout-spawn-egg-viability) * Trout-Fecundity-Effect))<br><br>; report round ((item trout-spp-index trout-spawn-fecund-mult) *<br>; (trout-length ^ (item trout-spp-index trout-spawn-fecund-exp)) *<br>; (item trout-spp-index trout-spawn-egg-viability))<br><br>end</pre>

Comparison of replicate number

The number of simulations performed for each scenario with each model were initially based on those from previous publications, based on the assumption that this was a sufficient number of runs to get a consistent population output. Hence, the stickleback model initially used 15 runs (after Mintram et al. 2018), trout model used 15 runs (after Forbes et al. 2019) and zebrafish model used 70 runs (after Hazlerigg et al. 2014).

However, in these initial simulations, a number of unusual features were observed in the results when compared against the EFSA (2023) assessment criteria. For example, a population "effect-response" (where a greater effect on the population would be expected when a greater magnitude of effect was imposed on individuals in the model) analogous to a dose-response in classical toxicity testing, was not always observed. Specifically, using the first assessment criterion (based on population means), a population effect was often recorded for only a limited number of timepoints over the 10-year simulation period, and occasionally these were at lower magnitudes of individual-level effect but not at higher ones. Furthermore, when a population effect was only observed at a limited number of timepoints, repeat simulations sometimes gave different population level outcomes.

Therefore, control simulations with increasing run numbers were performed with each model to explore how the population mean stabilised with increasing run numbers. The results for each model are shown in the following figure (Figure S1). This showed that by around 40 simulations, the population abundance of the zebrafish model had stabilised, meaning that a consistent result was recorded for the zebrafish model outputs when performing 70 runs. Meanwhile, the trout model showed that whilst variability in population outcome decreased with increasing number of runs (with abundance seemingly more sensitive than biomass, data not shown), the population mean had not yet stabilised when only 15 runs were performed. The model outputs showed that increasing the number of runs to 20 would stabilise the population mean. Similarly, for the stickleback, 15 simulations were insufficient to stabilise the population mean, but approx. 40 would result in a consistent model output. Each model differs in the stochastic processes within the model and this manifests in the variability in the outcomes.

To ensure a consistent model outcome (i.e. population response or not), a suitable number of model runs specific to each model is required. Following the investigations into run number and behaviour of the population mean in each model, the number of runs were increased to provide the final modelling and analysis presented in the main paper (i.e. increasing stickleback simulations from 15 to 70, increasing trout simulations from 15 to 20). This increase in the number of runs did address these inconsistencies across effect magnitudes. We would recommend that all models used in environmental risk assessment perform an analysis such as that performed here (qualitative following visual inspection of the outputs, or a more quantitative alternative) for these three models to identify a suitable level of replication.

Figure S1: Mean (and 1.96 x Standard Error of the Mean) for total abundance in the control simulation for each population model with differing numbers of runs

For control simulations of each model at the end of the warm-up period (3 years) with different numbers of model runs.

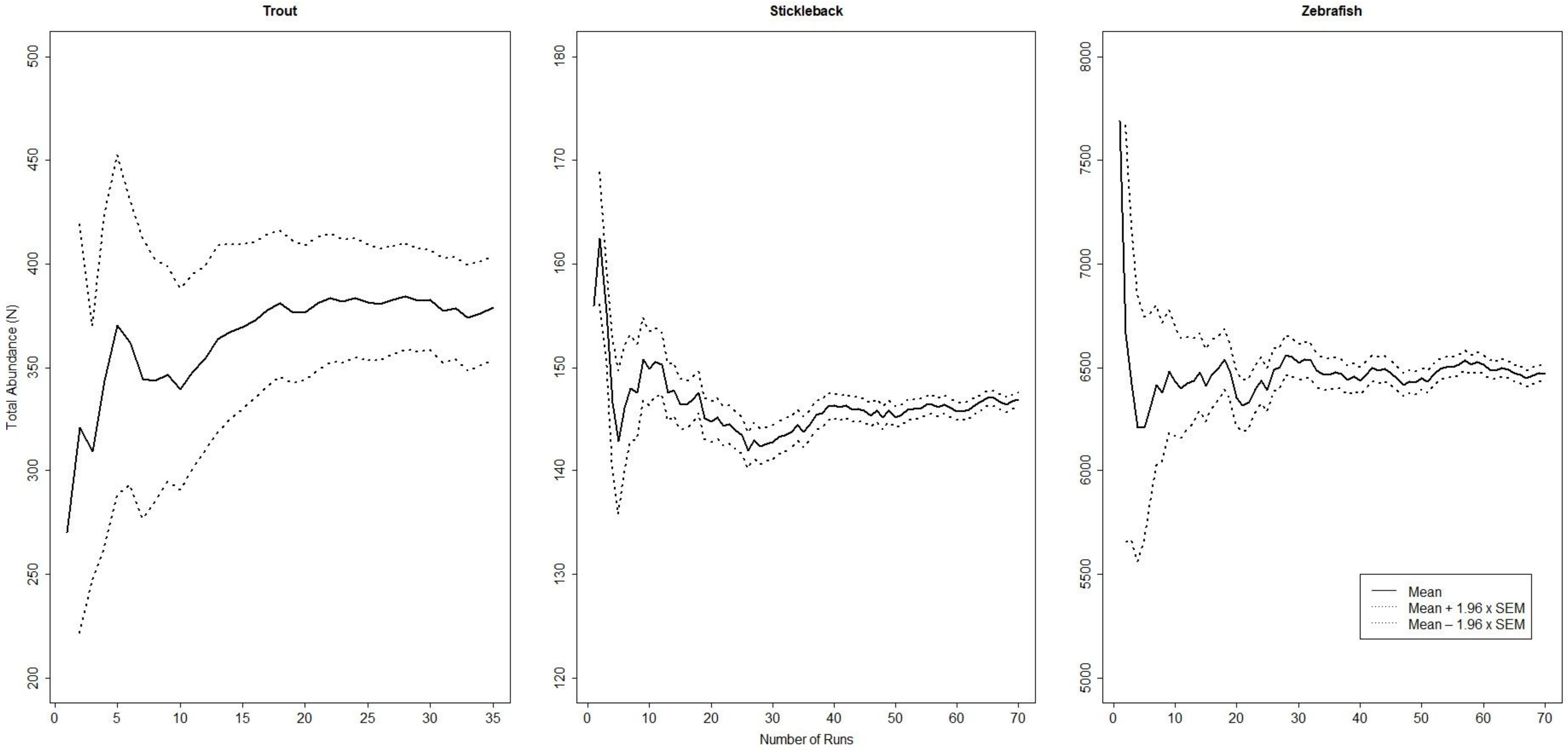

Table S7: Population results for biomass for the stickleback 10-year simulations, nesting behaviour endpoint, 15 and 70 replicates, indicating the passing and failing (number of days (d) failing the criterion) considering EFSA (2023) criterion 1 (i.e. the exposed population mean should not fall below the lower 95th percentile of the control).

Endpoint	Magnitude of effect	15 runs Biomass	70 runs Biomass

**Nesting behaviour**	**10%**	Fail (11 d)	Pass
	**20%**	Pass	Pass
	**50%**	Pass	Pass
	**90%**	Pass	Pass

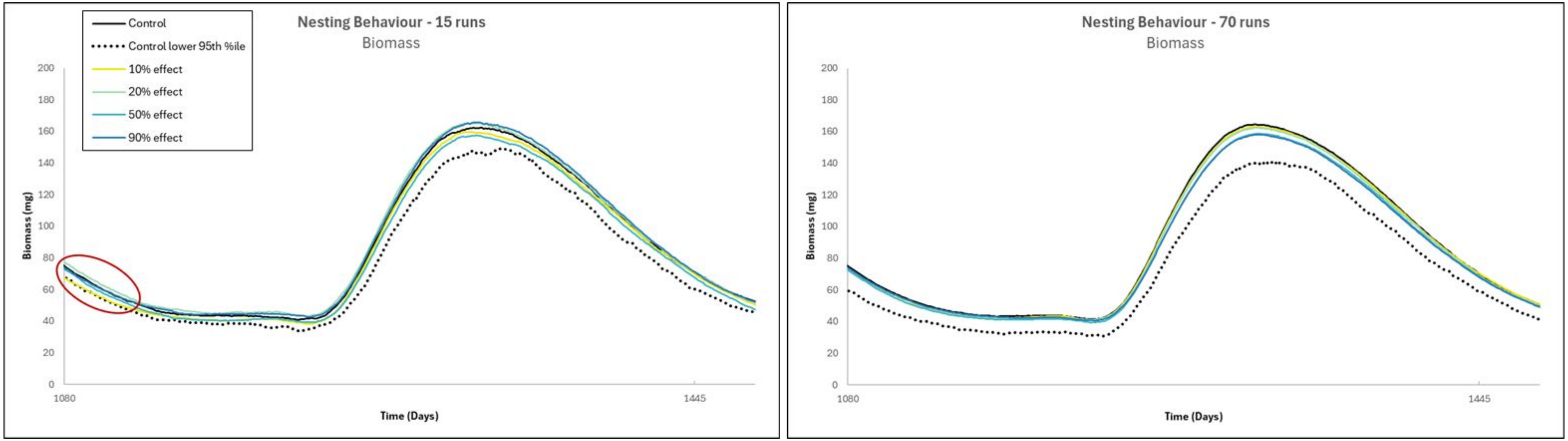

Figure S2: Population results for biomass for the stickleback 10-year simulations, nesting behaviour endpoint, 15 and 70 replicates. The simulations with 15 replicates show a failure of the assessment criterion just after 1000 days (ringed in red). When increasing replication this failure is no longer present. Note that this is not simply a result of increasing replication to decrease variability as used in traditional statistics, but rather a result of higher replication stabilising the lower 95th percentile giving a truer reflection of the average and normal operating range of the population.

Table S8: Population results for abundance for the trout 10-year simulations, sex ratio-male skew endpoint, 15 and 70 replicates, indicating the passing and failing (number of days (d) failing the criterion) considering EFSA (2023) criterion 1 (i.e. the exposed population mean should not fall below the lower 95th percentile of the control).

Endpoint	Magnitude of effect	15 runs Abundance	20 runs Abundance
**Sex Ratio_Male skew**	**10%**	Fail (20 d)	Pass
	**20%**	Pass	Pass
	**50%**	Fail (868.5 d)	Fail (420.75 d)
	**90%**	Fail (1812 d)	Fail (1753 d)

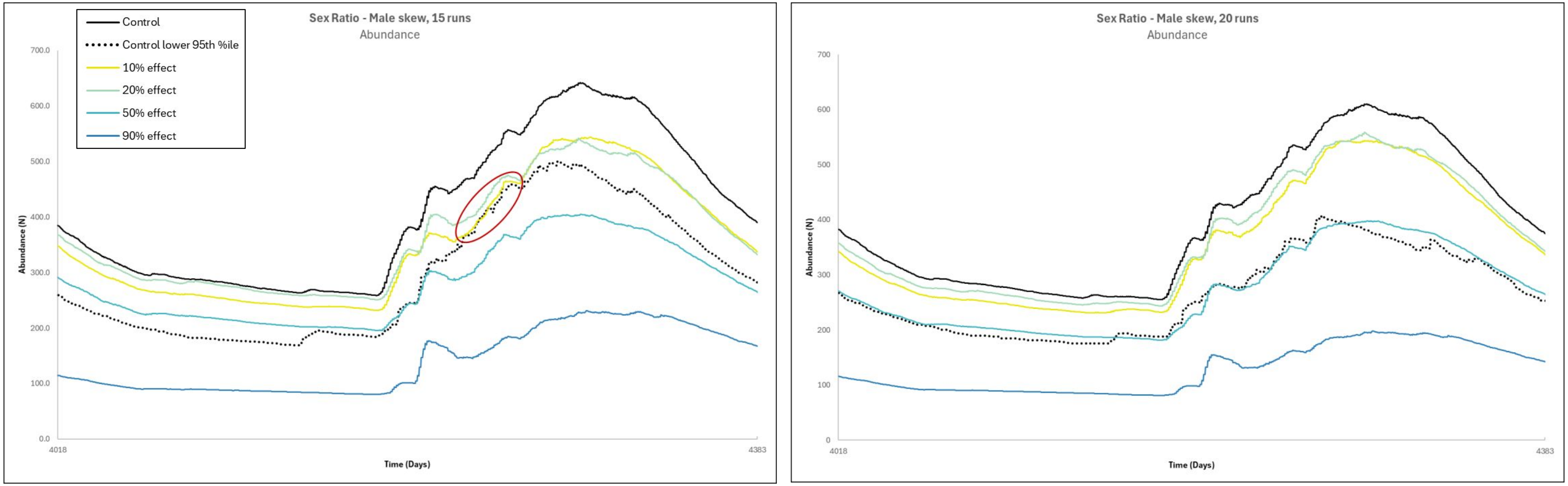

Figure S3: Population results for abundance for the trout 10-year simulations, sex ratio-male skew endpoint, 15 and 20 replicates. The simulations with 15 replicates show a failure of the assessment criterion just after 1000 days (ringed in red). When increasing replication this failure is no longer present. Note that this is not simply a result of increasing replication to decrease variability as used in traditional statistics, but rather a result of higher replication stabilising the lower 95th percentile giving a truer reflection of the average and normal operating range of the population.

Graphs for the 10-year of Continuous Effects

Legend: Control; Control lower 95th %ile; 10% effects; 20% effects; 50% effects; 90% effects

Zebrafish

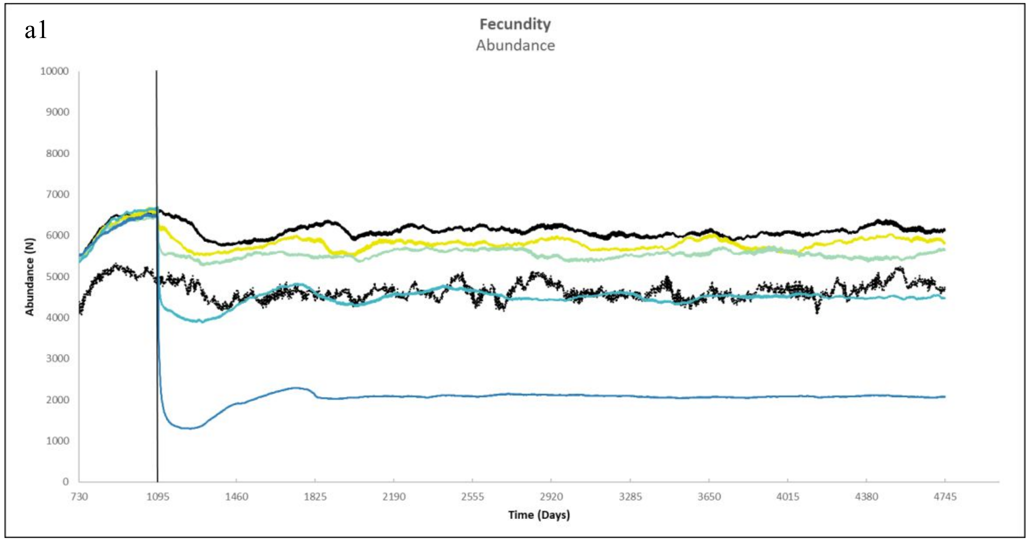

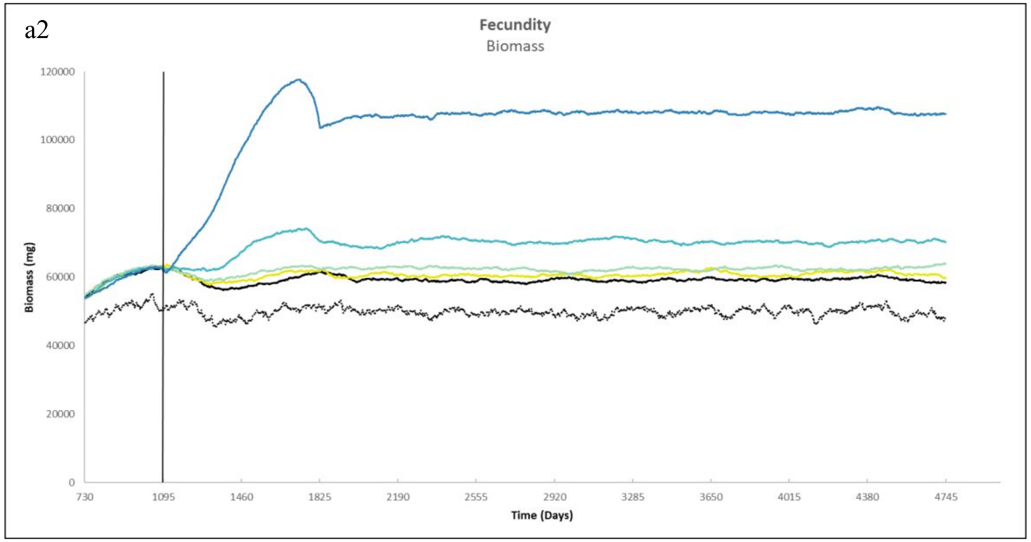

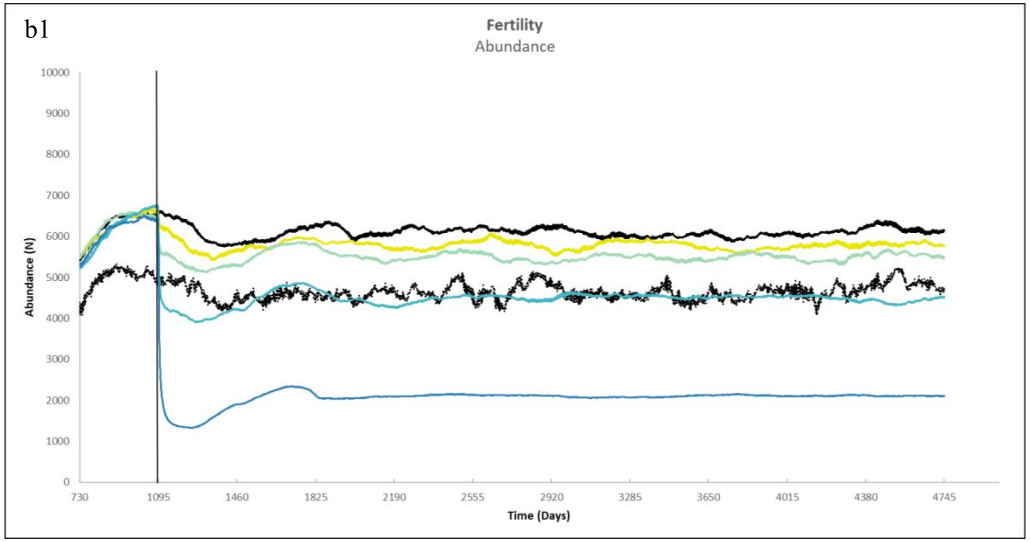
b1
Fertility
Abundance
Abundance (N)
10000
9000
8000
7000
6000
5000
4000
3000
2000
1000
0
730 1095 1460 1825 2190 2555 2920 3285 3650 4015 4380 4745
Time (Days)

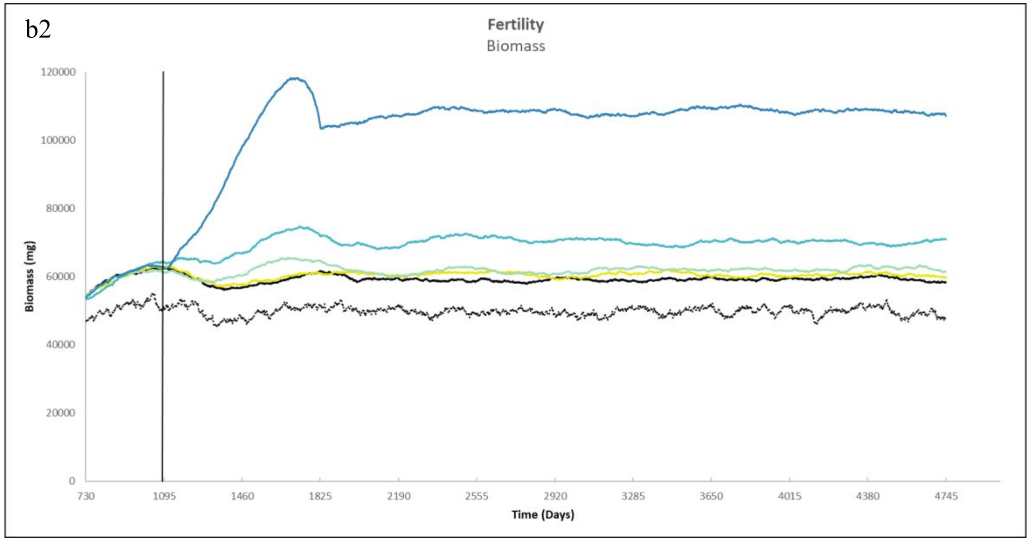
b2
Fertility
Biomass
Biomass (mg)
120000
100000
80000
60000
40000
20000
0
730 1095 1460 1825 2190 2555 2920 3285 3650 4015 4380 4745
Time (Days)

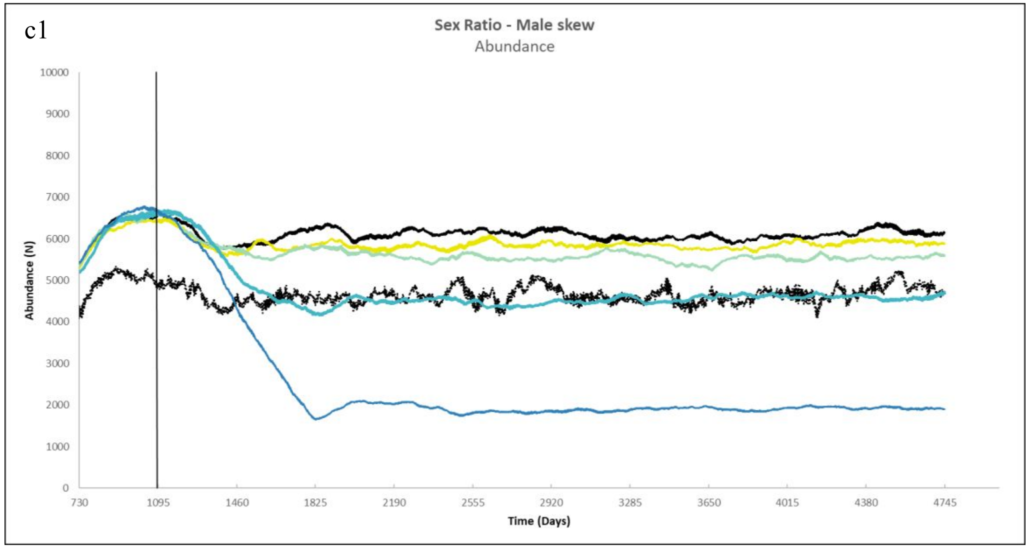
c1
Sex Ratio - Male skew
Abundance
Abundance (N)
10000
9000
8000
7000
6000
5000
4000
3000
2000
1000
0
730 1095 1460 1825 2190 2555 2920 3285 3650 4015 4380 4745
Time (Days)

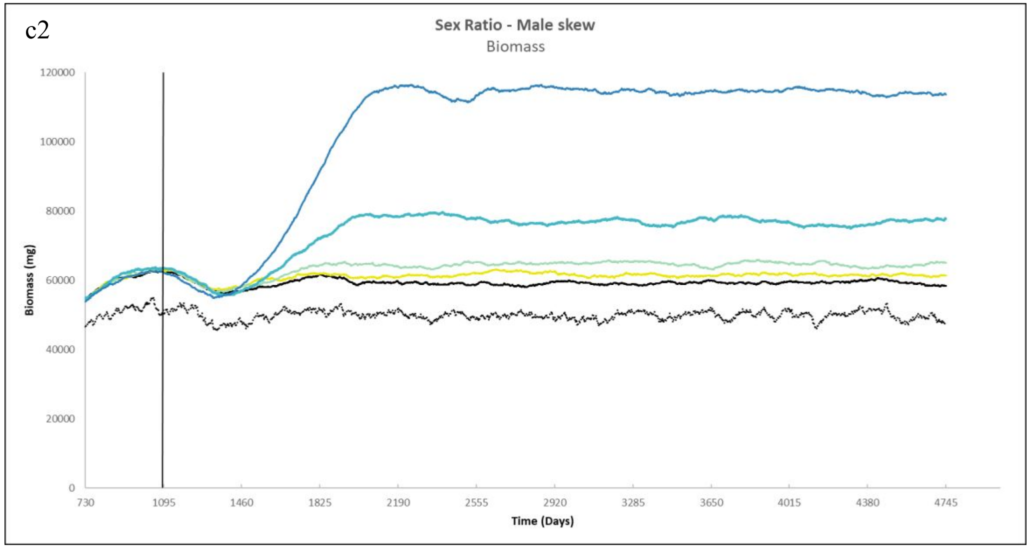
c2
Sex Ratio - Male skew
Biomass
Biomass (mg)
120000
100000
80000
60000
40000
20000
0
730 1095 1460 1825 2190 2555 2920 3285 3650 4015 4380 4745
Time (Days)

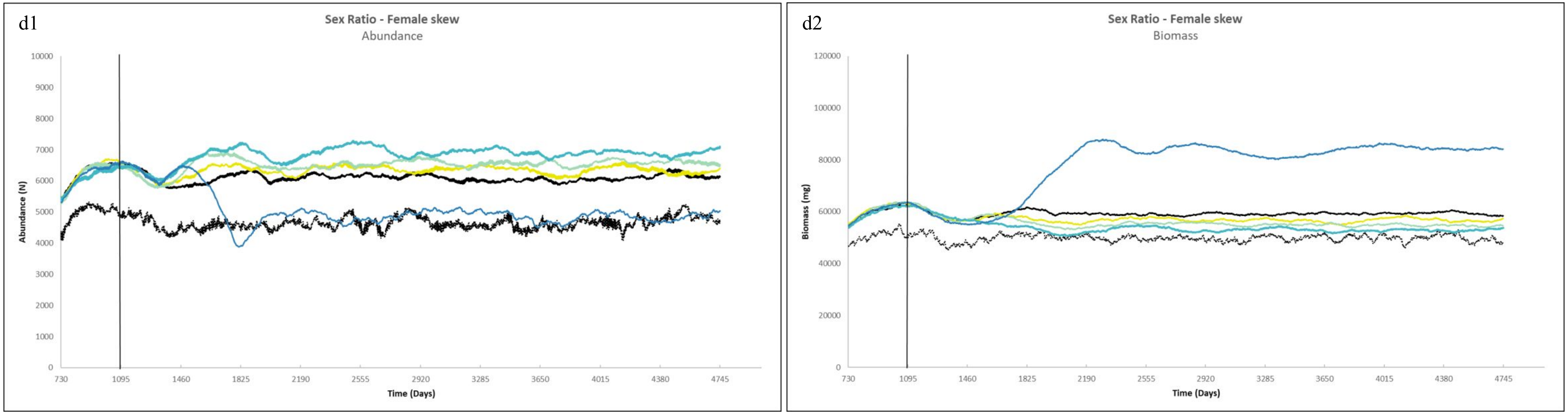

Figure S4: Population results plots for the zebrafish 10-year simulations of continuous effects: fecundity, fertility, sex ratio male and female skew, for abundance (a1, b1, c1, d1) and biomass (a2, b2, c2, d2). The vertical line at 1095 days indicates the starting of the exposure period.

Stickleback

a1

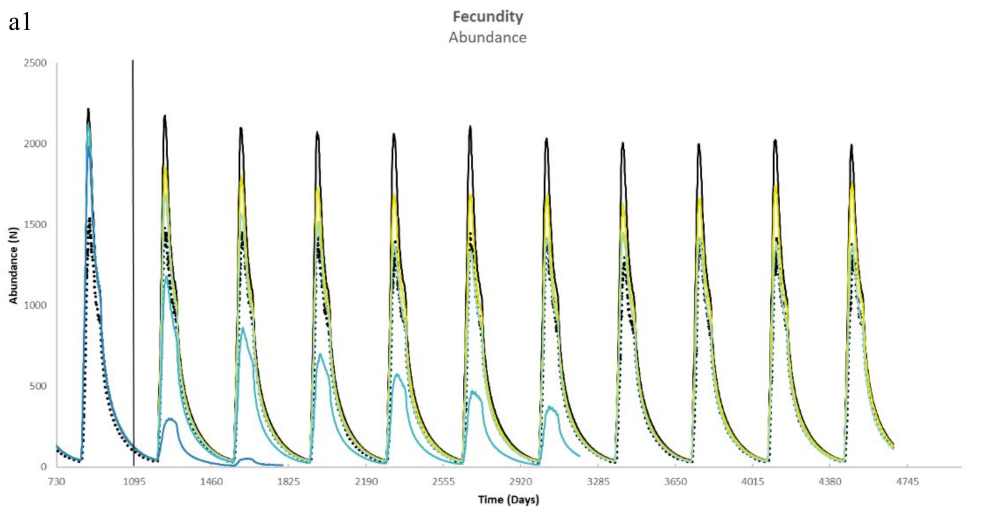

a2

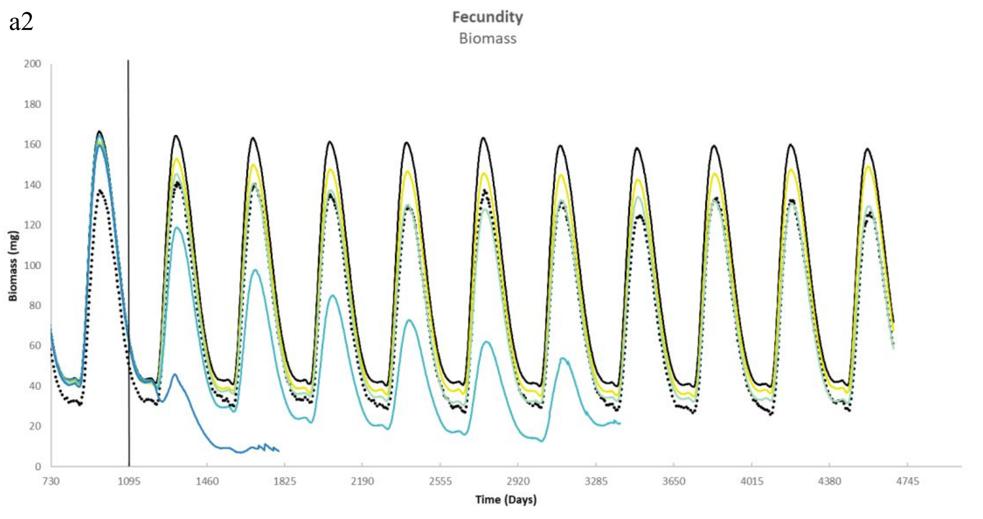

b1

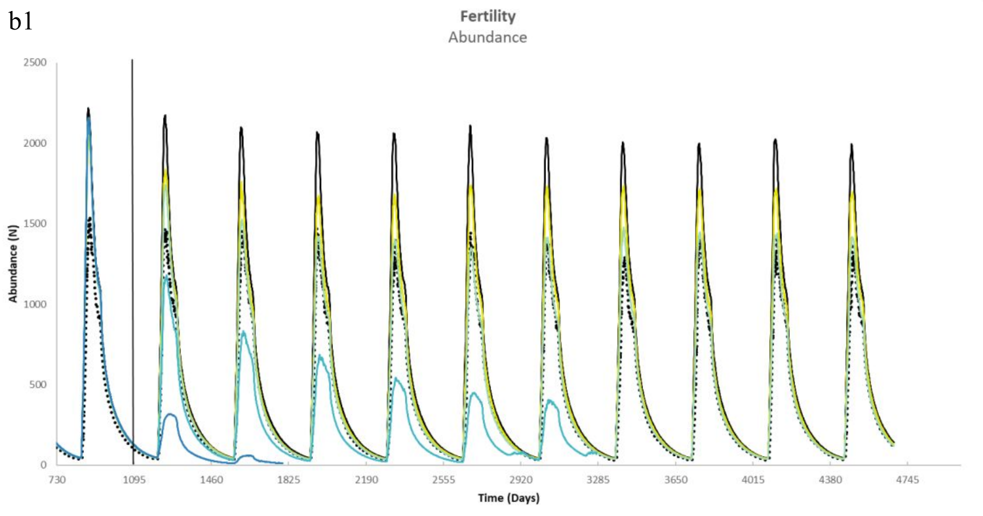

b2

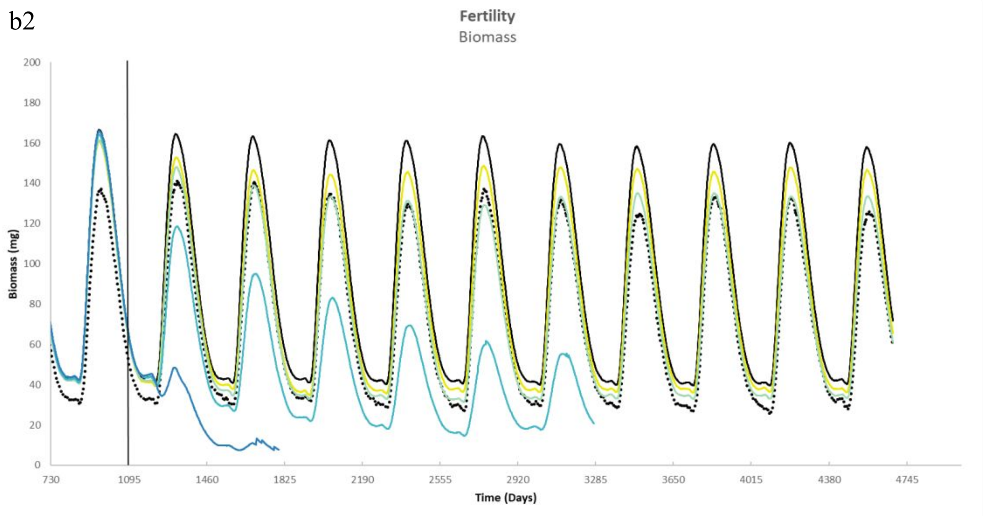

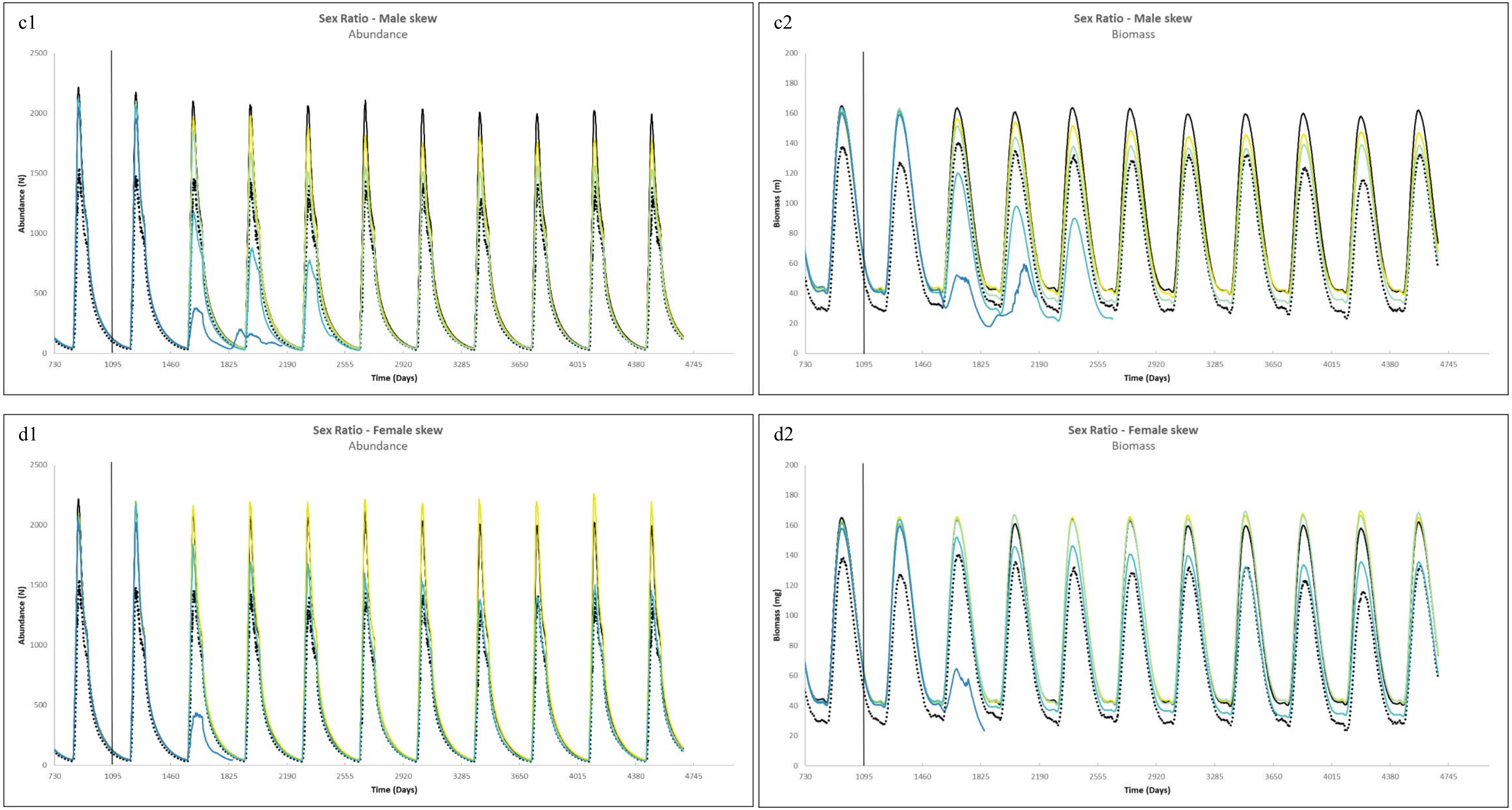
c1
Sex Ratio - Male skew
Abundance
Abundance (N)
Time (Days)
c2
Sex Ratio - Male skew
Biomass
Biomass (m)
Time (Days)
d1
Sex Ratio - Female skew
Abundance
Abundance (N)
Time (Days)
d2
Sex Ratio - Female skew
Biomass
Biomass (mg)
Time (Days)

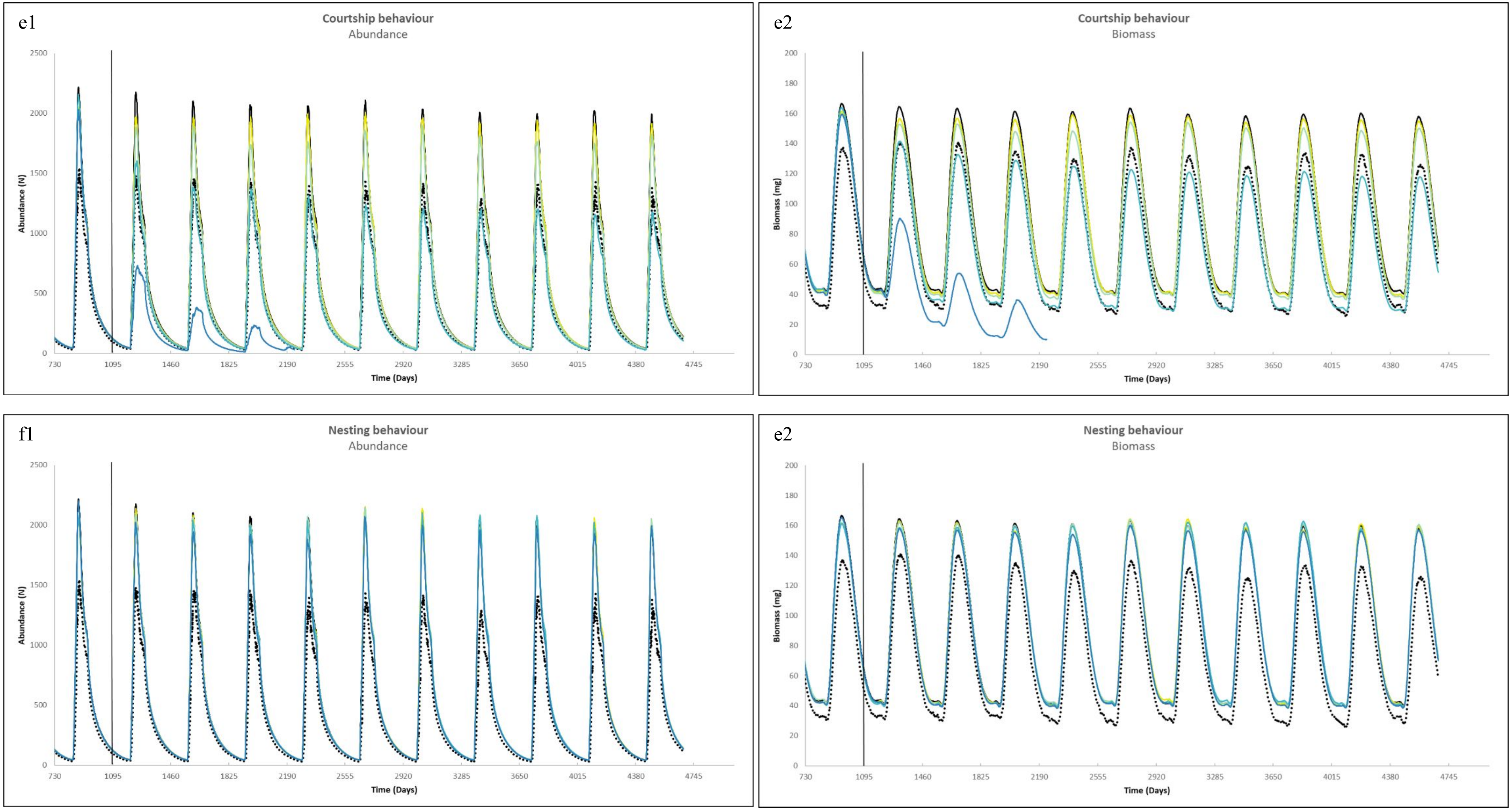

Figure S5: Population results plots for the stickleback 10-year simulations of continuous effects: fecundity, fertility, sex ratio male and female skew, courtship and nesting behaviour, for abundance (a1, b1, c1, d1, e1, f1) and biomass (a2, b2, c2, d2, e2, f2). The vertical line at 1095 days indicates the starting of the exposure period.

Trout

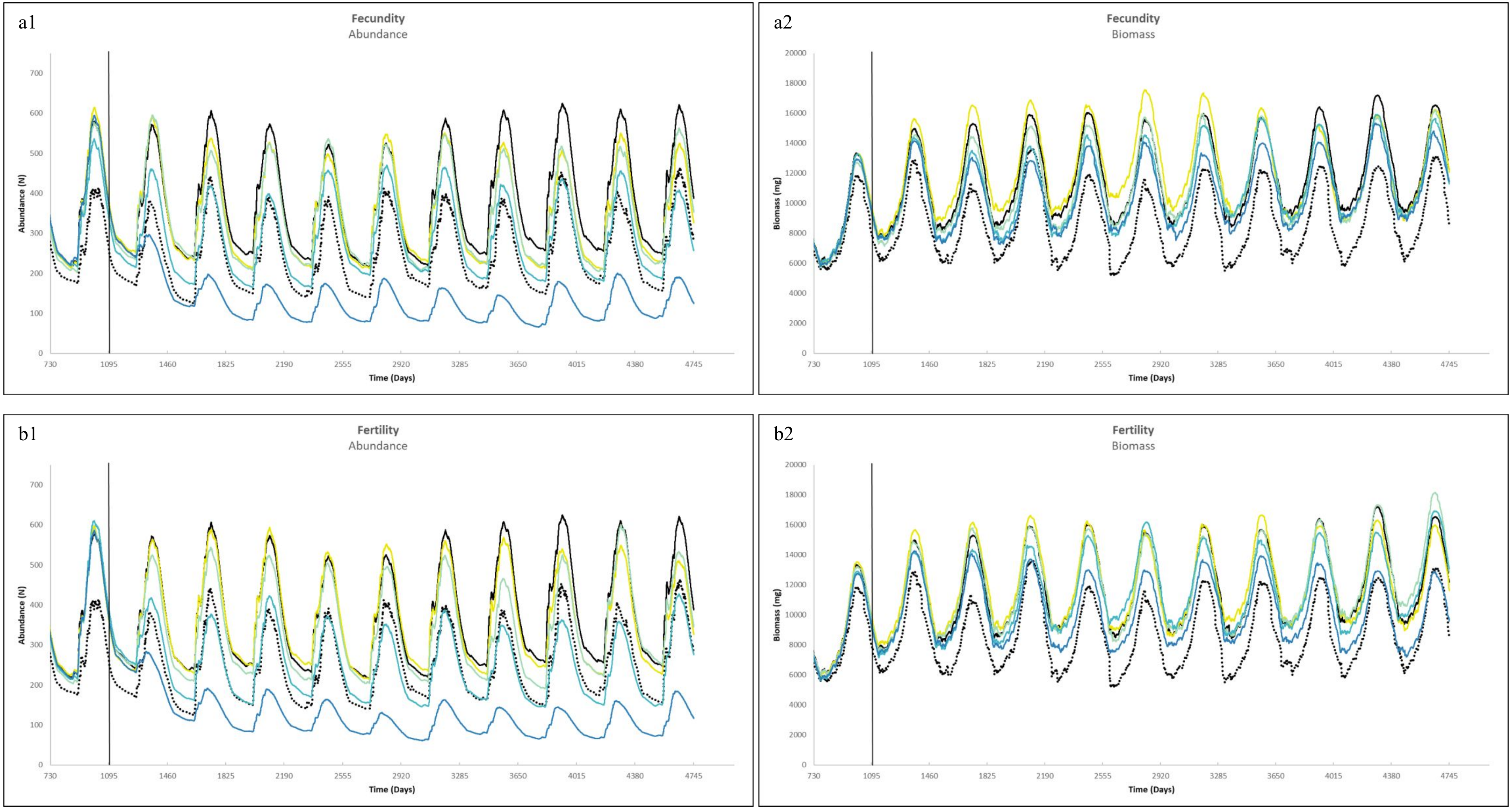
a1
Fecundity
Abundance
Abundance (N)
Time (Days)
a2
Fecundity
Biomass
Biomass (mg)
Time (Days)
b1
Fertility
Abundance
Abundance (N)
Time (Days)
b2
Fertility
Biomass
Biomass (mg)
Time (Days)

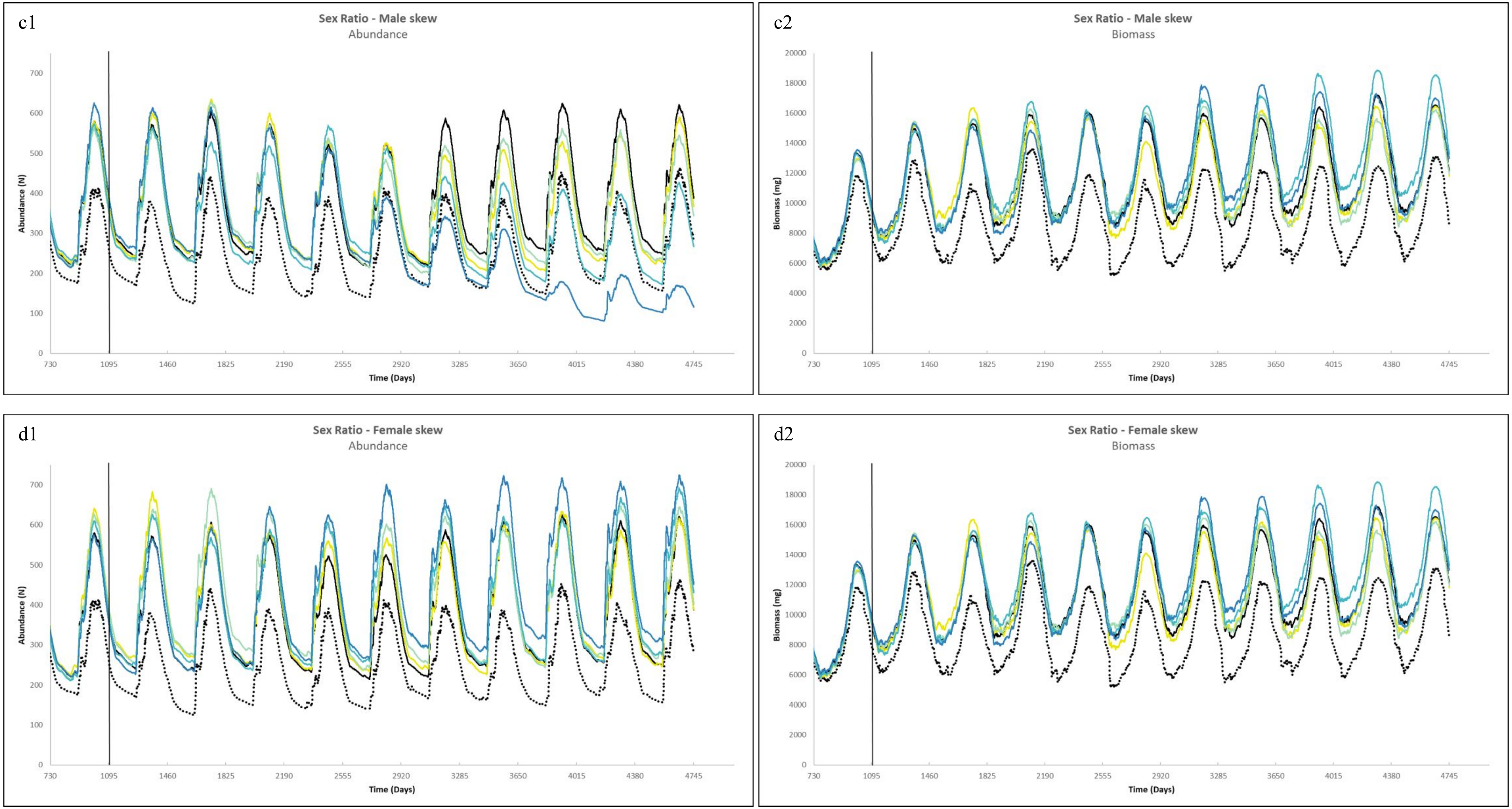

Figure S6: Population results plots for the trout 10-year simulations of continuous effects: fecundity, fertility, sex ratio male and female skew, for abundance (a1, b1, c1, d1) and biomass (a2, b2, c2, d2). The vertical line at 1095 days indicates the starting of the exposure period.

Graphs for the 10-years of Seasonal Effects

Legend: Control; Control lower 95th %ile; 10% effects; 20% effects; 50% effects; 90% effects

Stickleback Summer

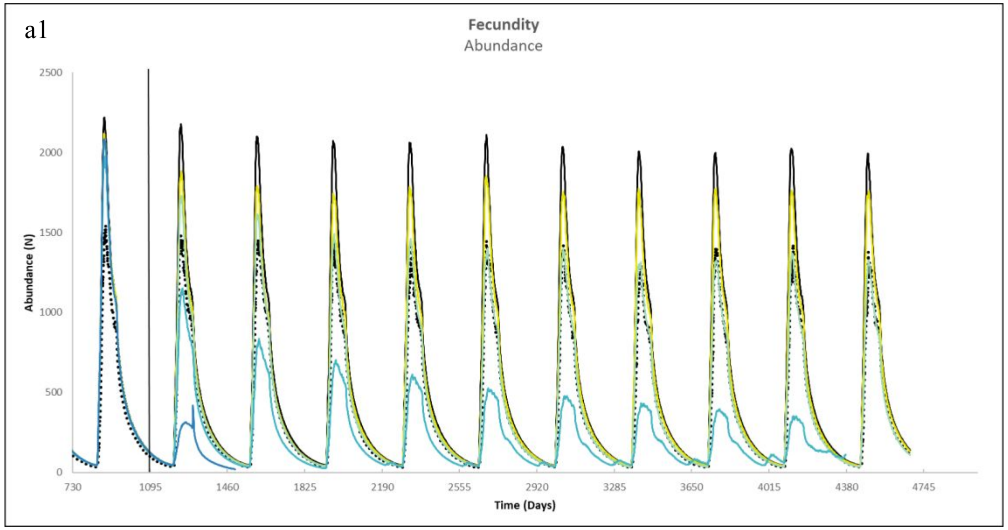

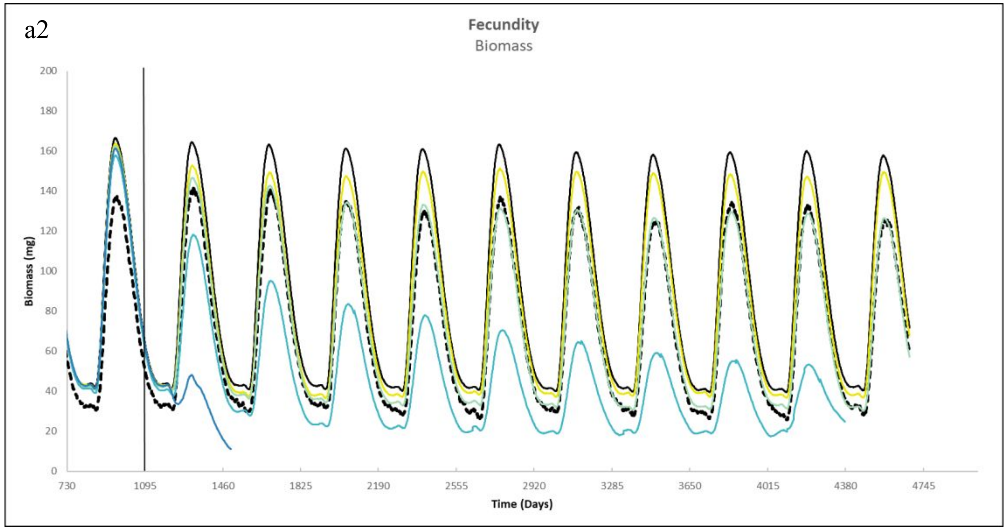

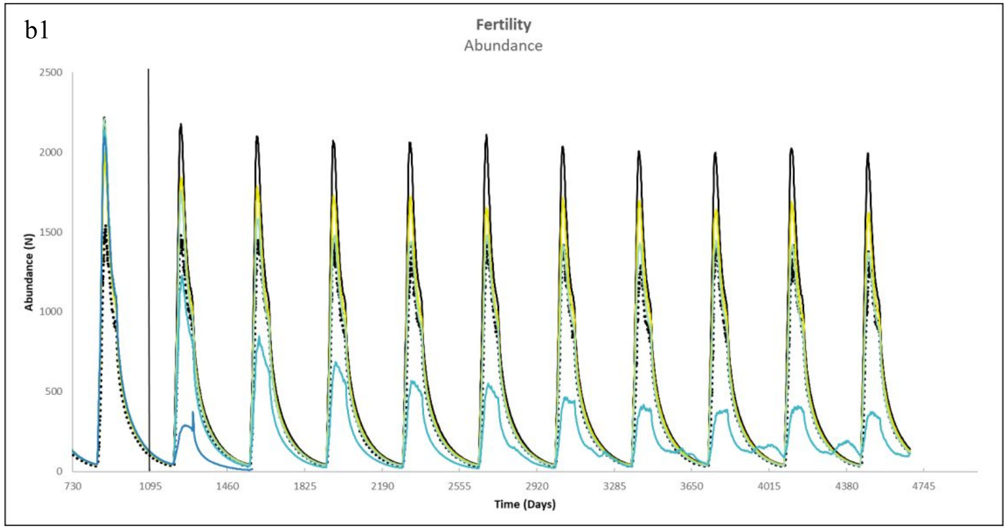

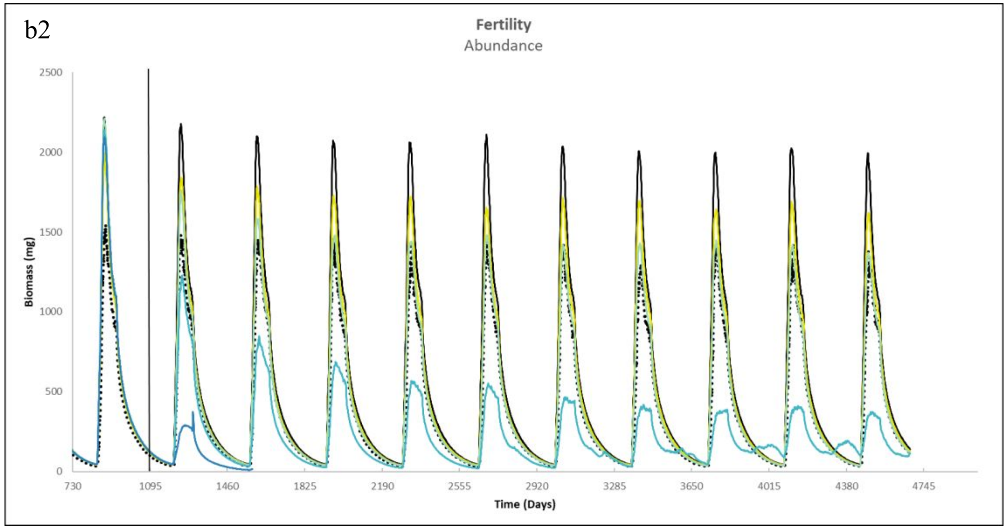

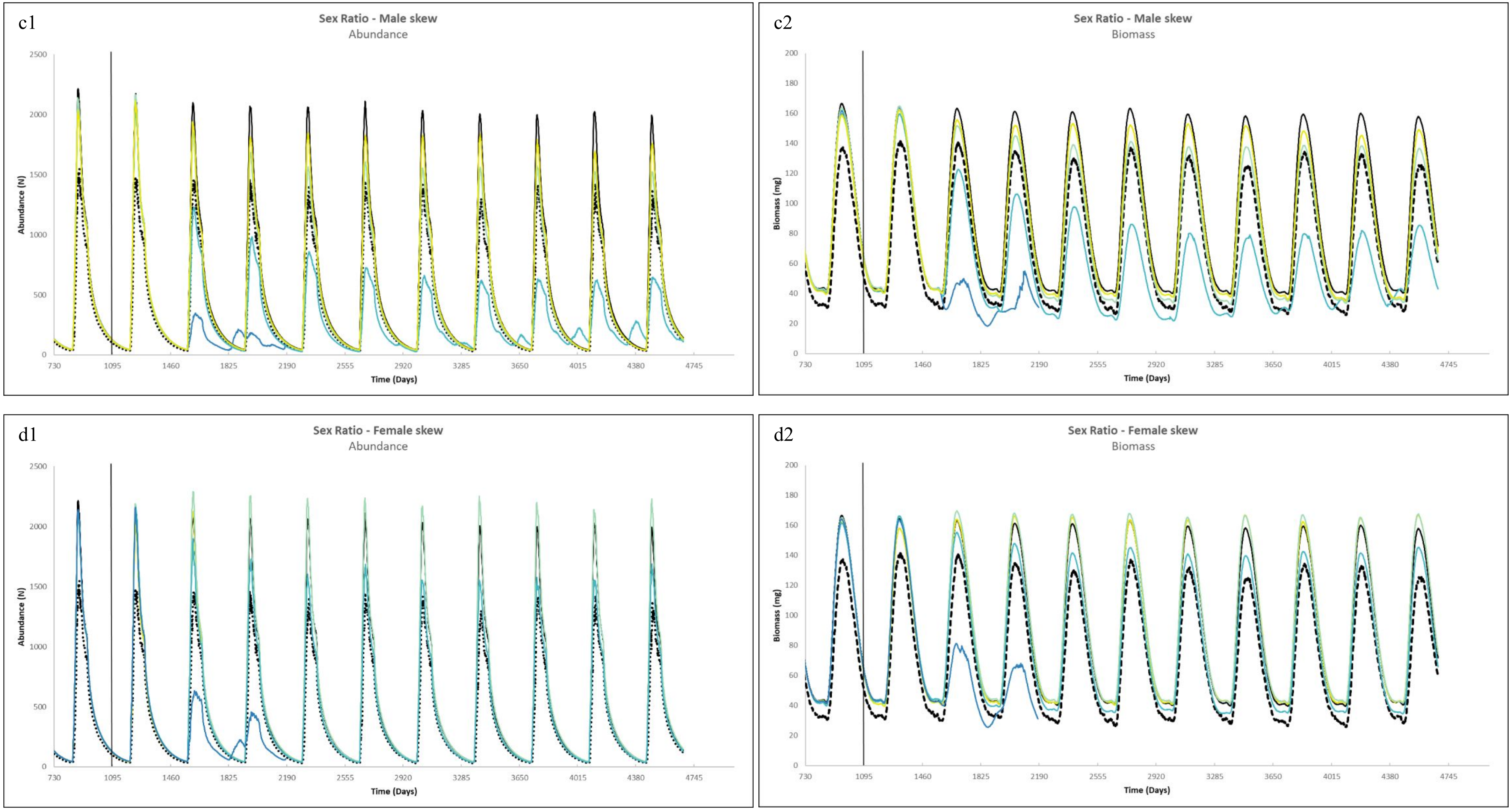
c1
Sex Ratio - Male skew
Abundance
Abundance (N)
2500
2000
1500
1000
500
0
730
1095
1460
1825
2190
2555
2920
3285
3650
4015
4380
4745
Time (Days)
c2
Sex Ratio - Male skew
Biomass
Biomass (mg)
200
180
160
140
120
100
80
60
40
20
0
730
1095
1460
1825
2190
2555
2920
3285
3650
4015
4380
4745
Time (Days)
d1
Sex Ratio - Female skew
Abundance
Abundance (N)
2500
2000
1500
1000
500
0
730
1095
1460
1825
2190
2555
2920
3285
3650
4015
4380
4745
Time (Days)
d2
Sex Ratio - Female skew
Biomass
Biomass (mg)
200
180
160
140
120
100
80
60
40
20
0
730
1095
1460
1825
2190
2555
2920
3285
3650
4015
4380
4745
Time (Days)

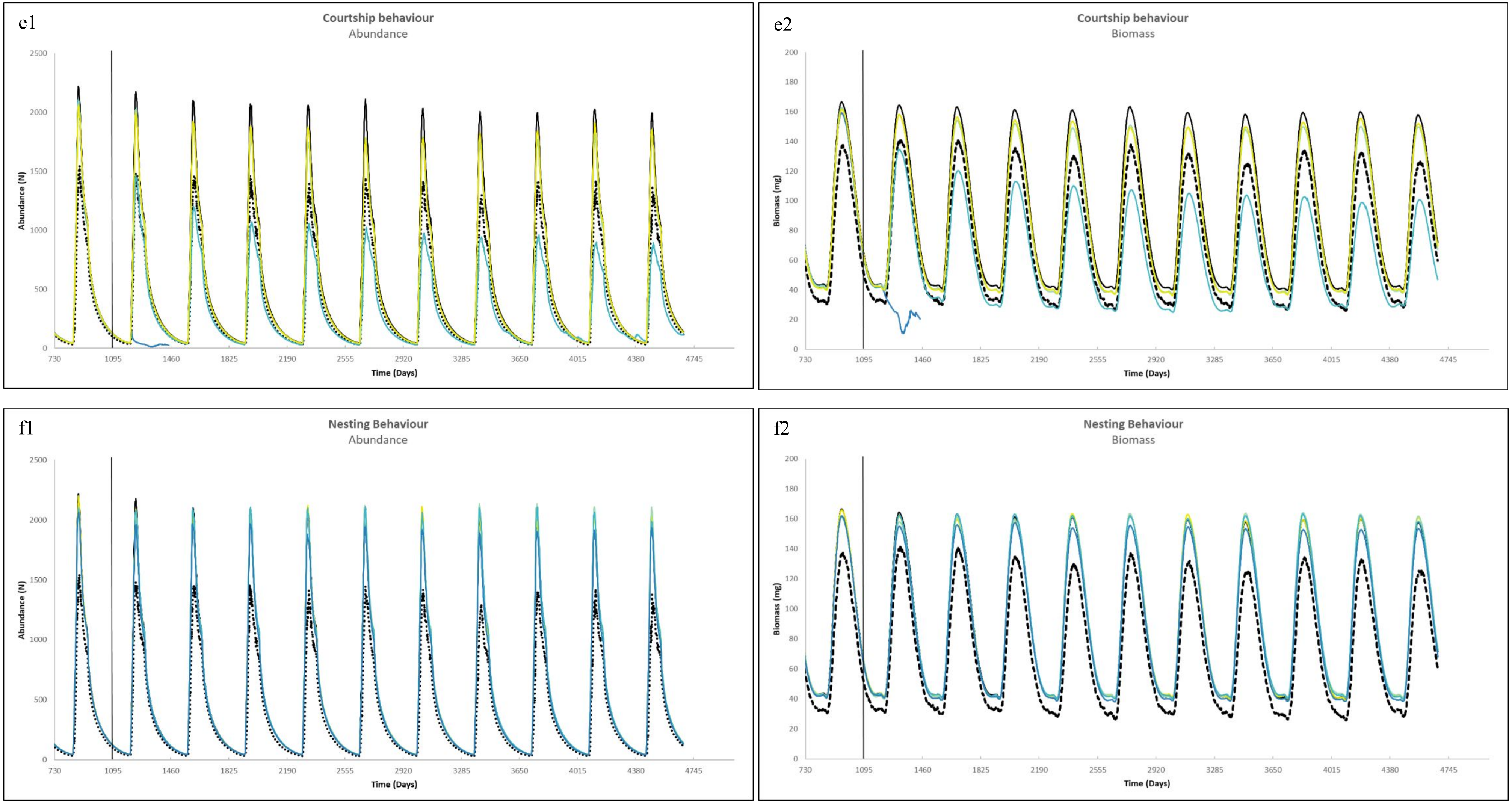

Figure S7: Population results plots for the stickleback 10-year simulations of seasonal effects in summer: fecundity, fertility, sex ratio male and female skew, courtship and nesting behaviour, for abundance (a1, b1, c1, d1, e1, f1) and biomass (a2, b2, c2, d2, e2, f2). The vertical line at 1095 days indicates the starting of the exposure period.

Stickleback Winter

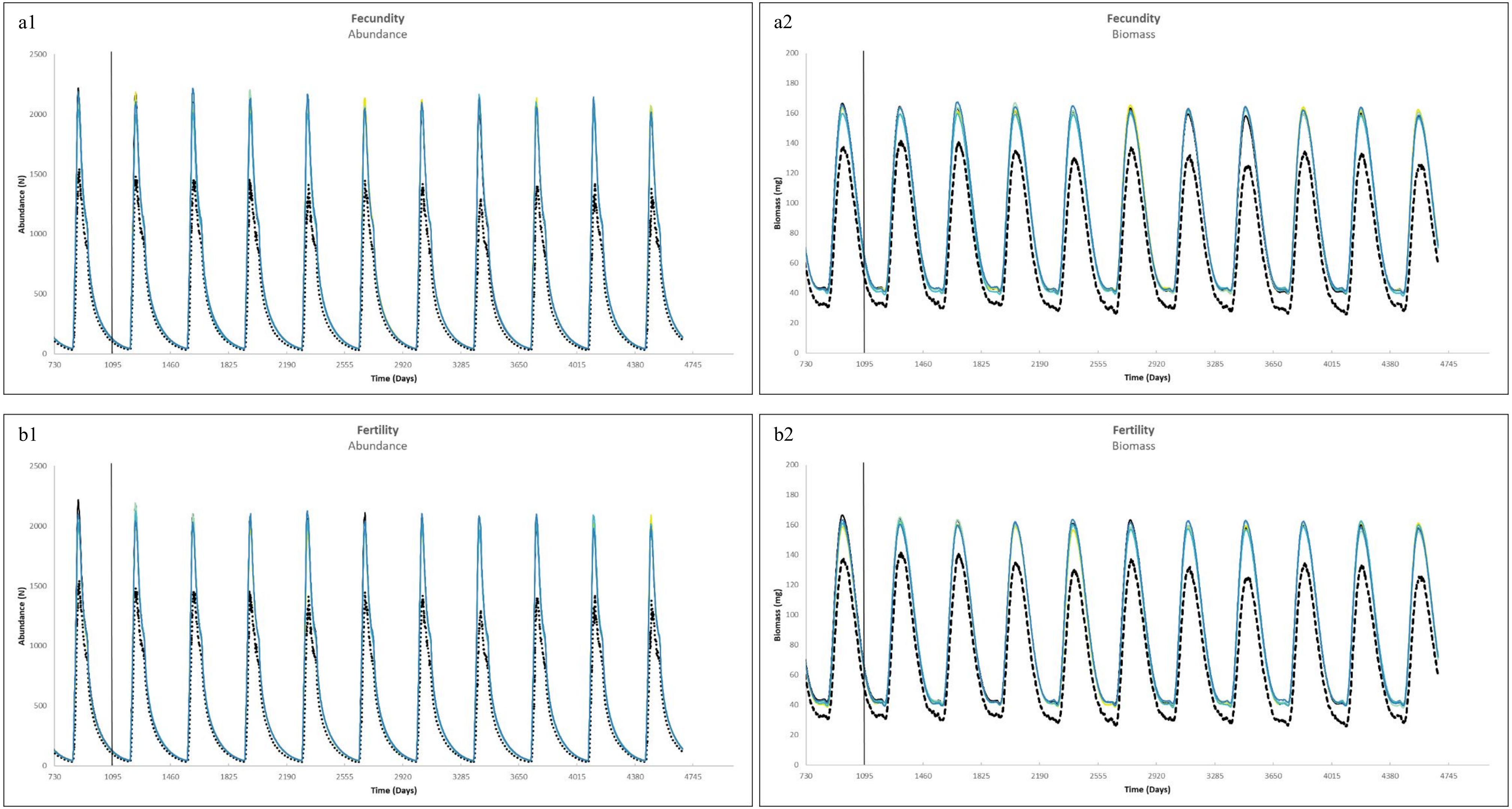

c1

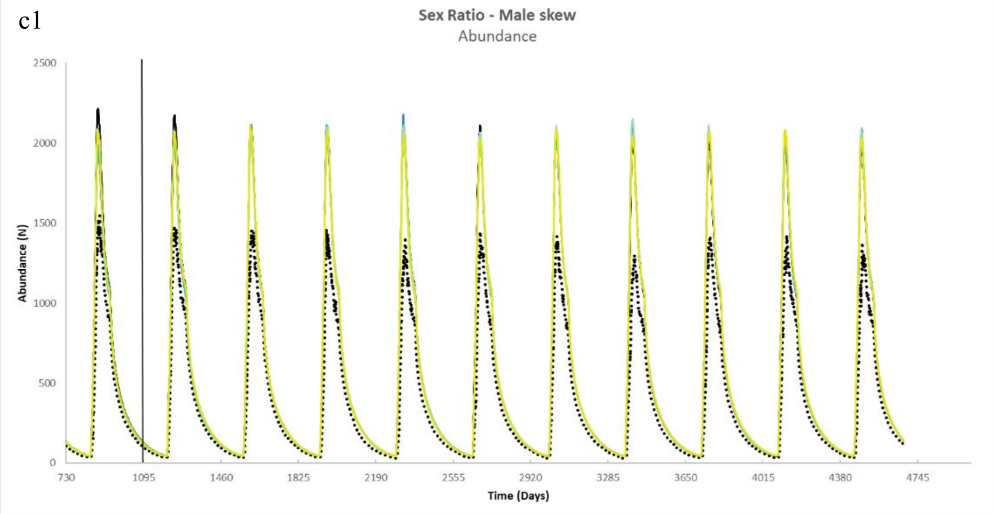
Sex Ratio - Male skew
Abundance
Abundance (N)
Time (Days)
0
500
1000
1500
2000
2500
730
1095
1460
1825
2190
2555
2920
3285
3650
4015
4380
4745

c2

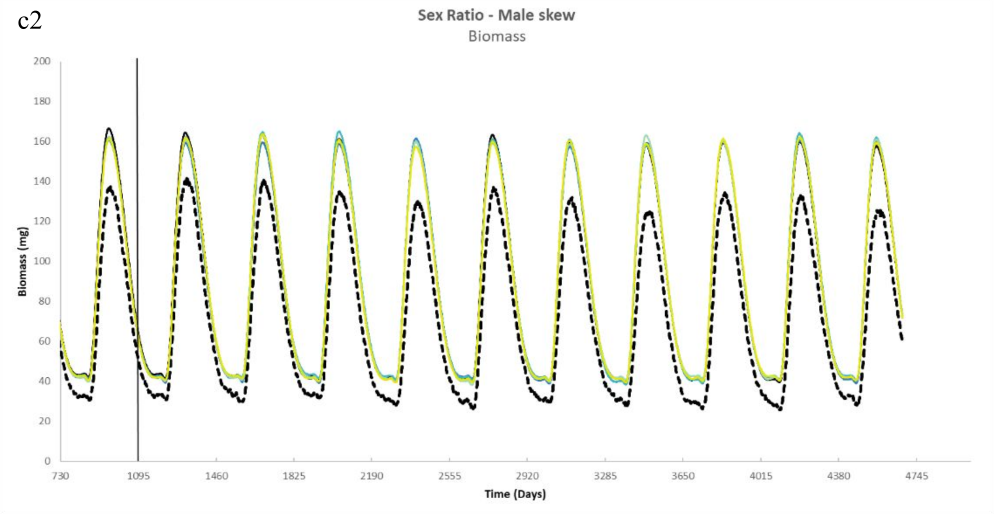
Sex Ratio - Male skew
Biomass
Biomass (mg)
Time (Days)
0
20
40
60
80
100
120
140
160
180
200
730
1095
1460
1825
2190
2555
2920
3285
3650
4015
4380
4745

d1

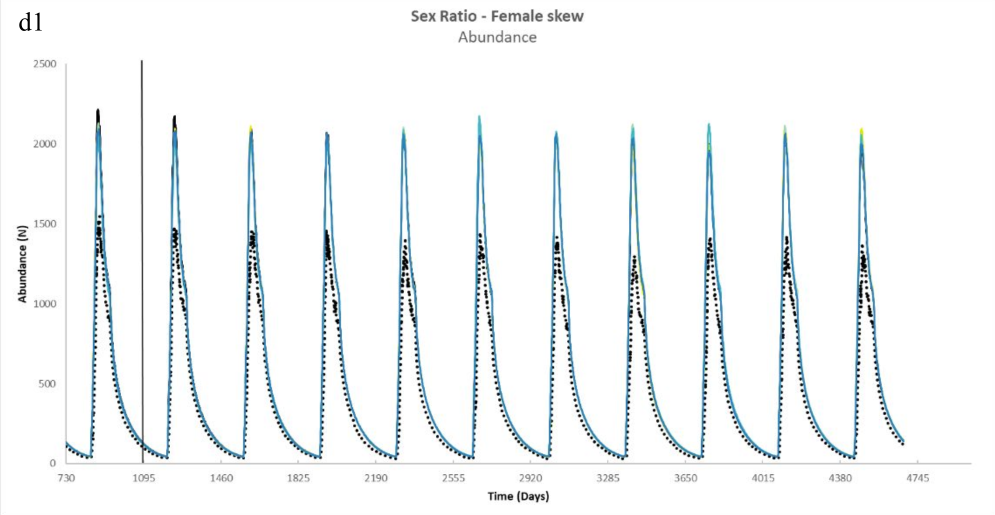
Sex Ratio - Female skew
Abundance
Abundance (N)
Time (Days)
0
500
1000
1500
2000
2500
730
1095
1460
1825
2190
2555
2920
3285
3650
4015
4380
4745

d2

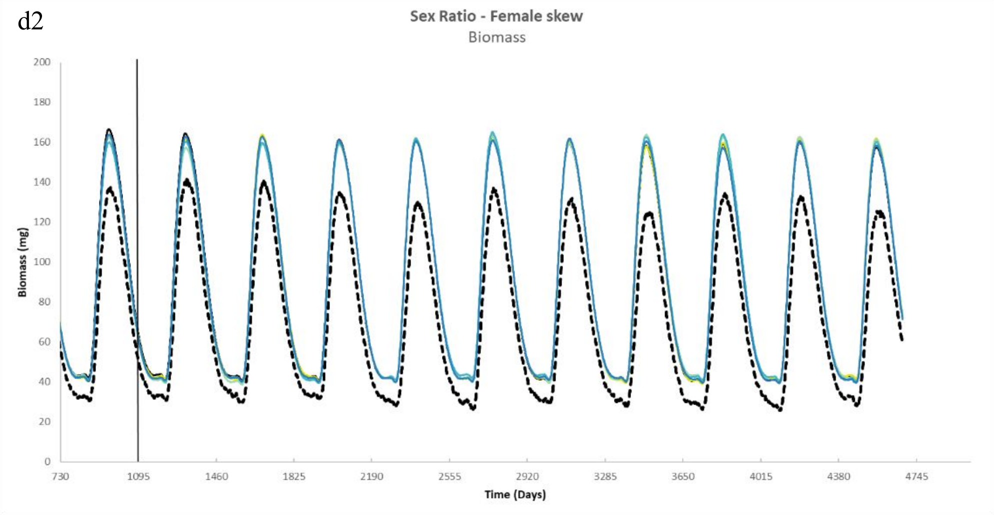
Sex Ratio - Female skew
Biomass
Biomass (mg)
Time (Days)
0
20
40
60
80
100
120
140
160
180
200
730
1095
1460
1825
2190
2555
2920
3285
3650
4015
4380
4745

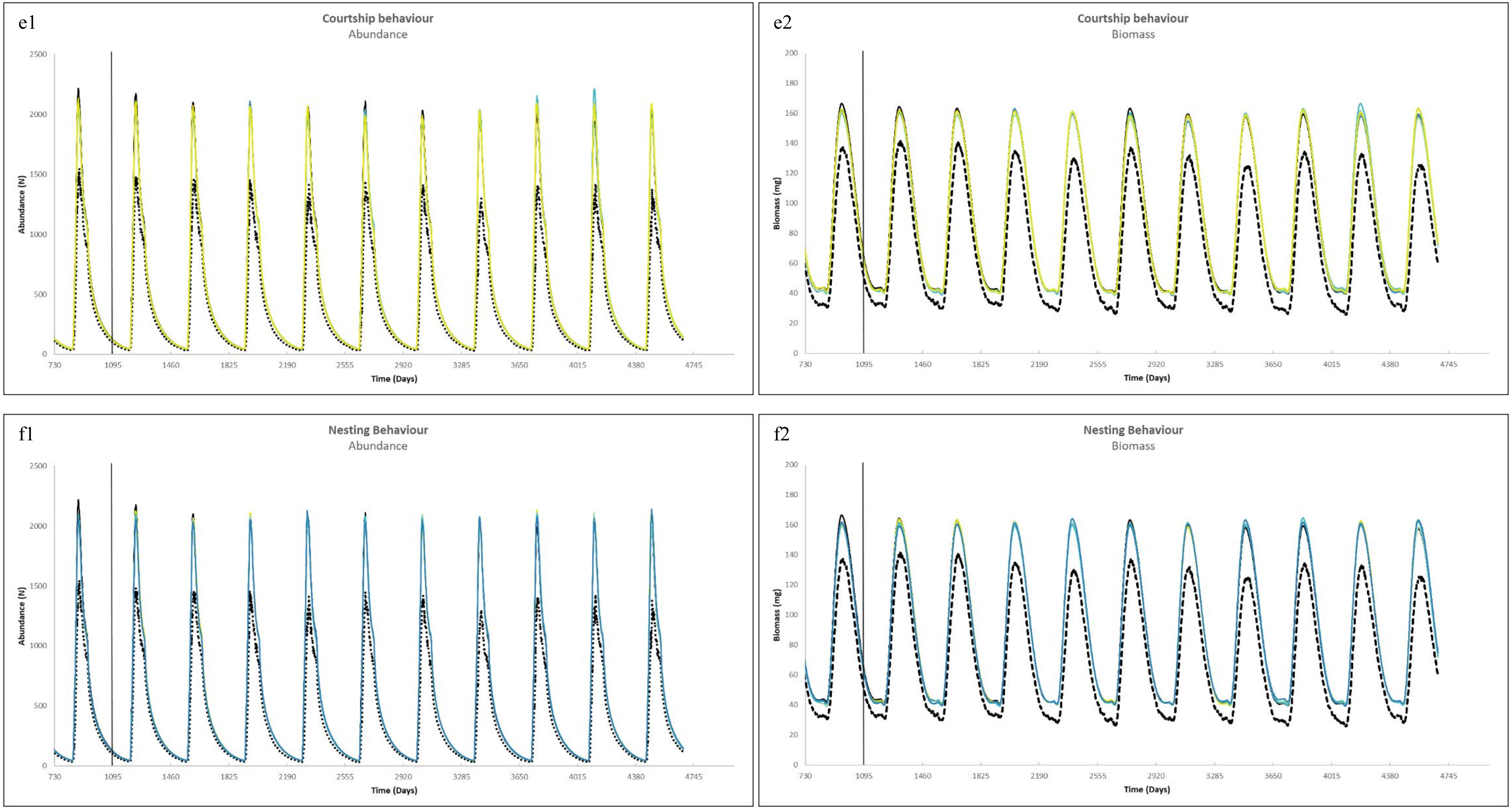

Figure S8: Population results plots for the stickleback 10-year simulations of seasonal effects in winter: fecundity, fertility, sex ratio male and female skew, courtship and nesting behaviour, for abundance (a1, b1, c1, d1, e1, f1) and biomass (a2, b2, c2, d2, e2, f2). The vertical line at 1095 days indicates the starting of the exposure period.

Trout Summer

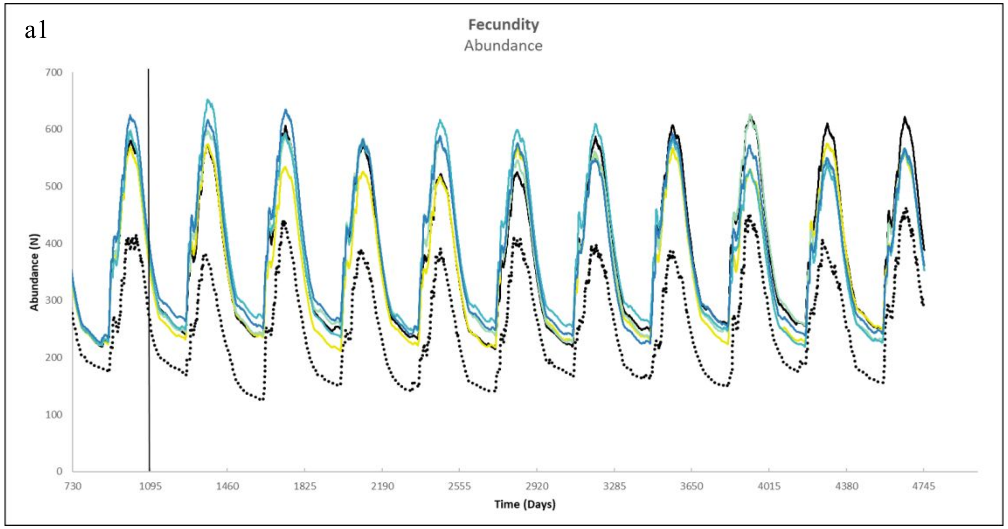

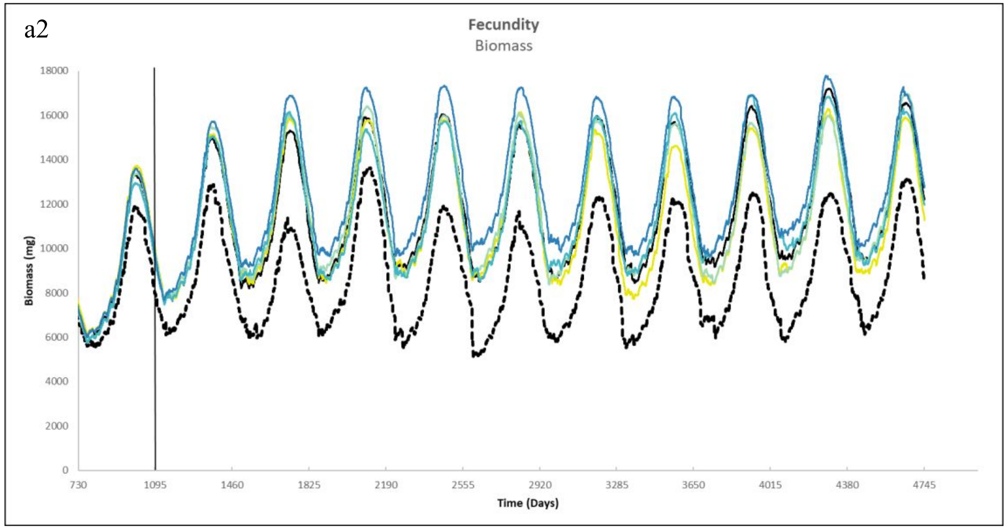

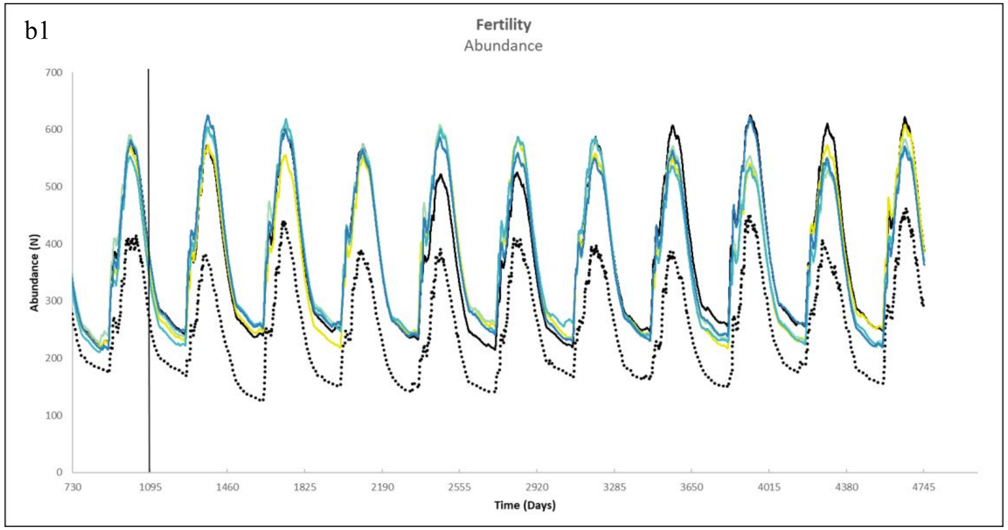

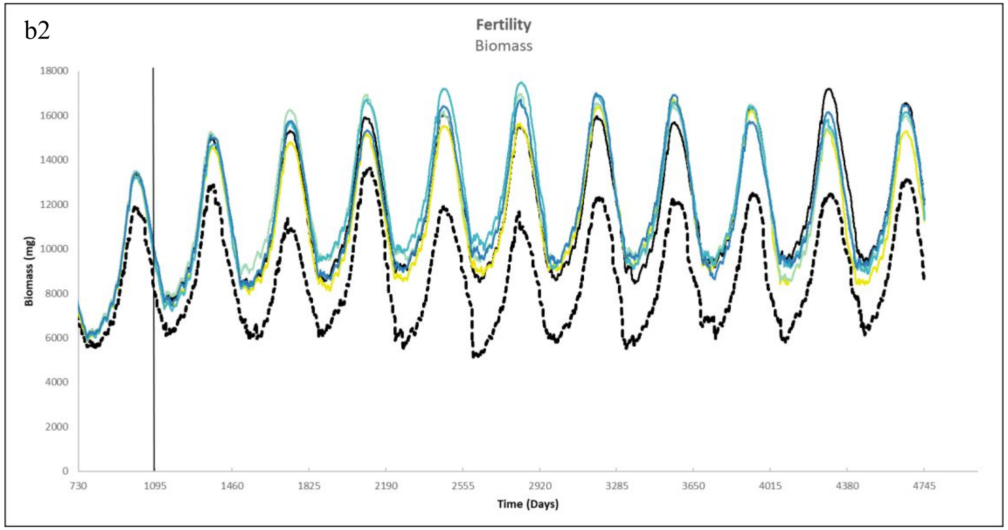

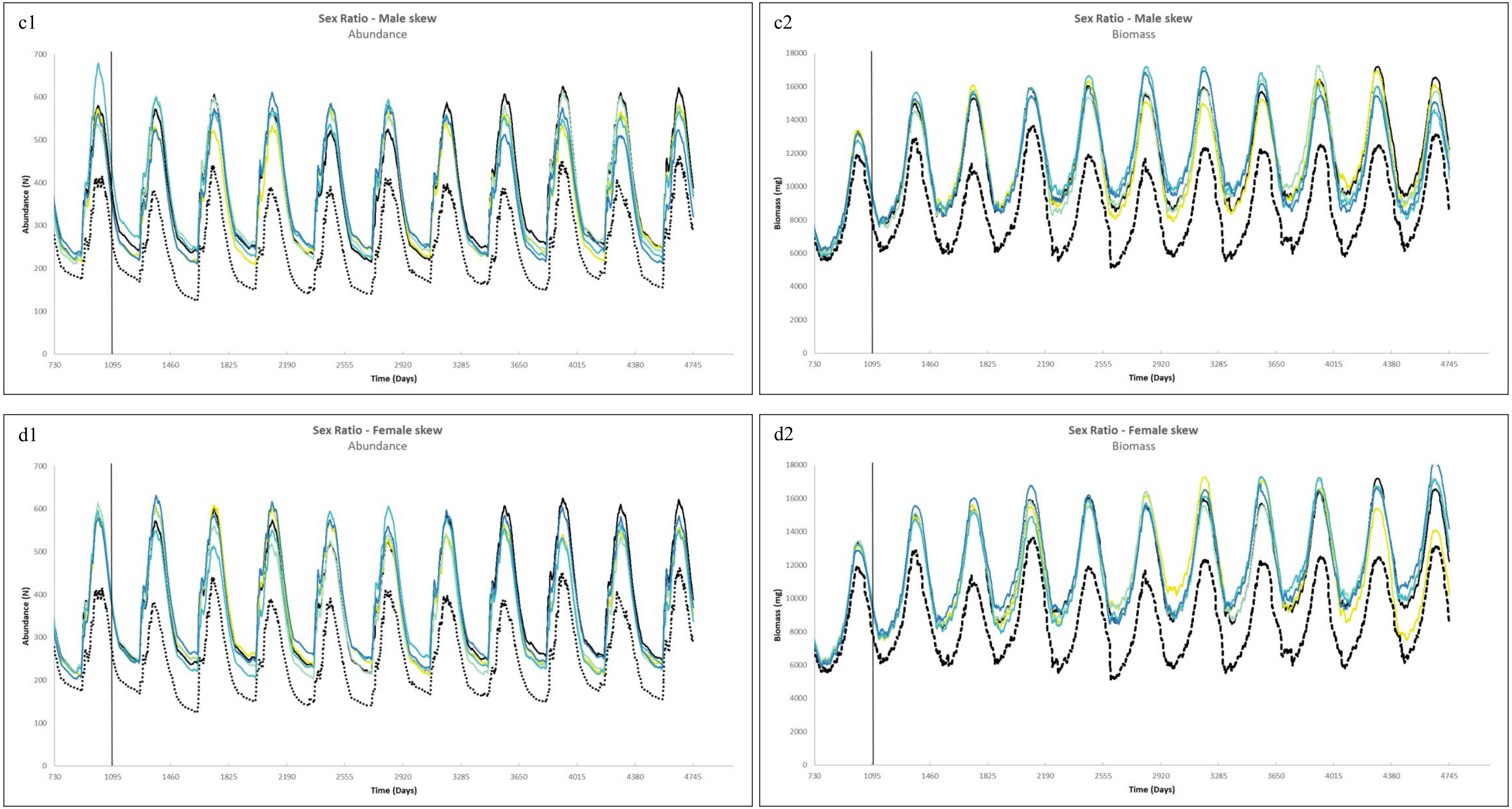

Figure S9: Population results plots for the trout 10-year simulations of seasonal effects in summer: fecundity, fertility, sex ratio male and female skew, for abundance (a1, b1, c1, d1) and biomass (a2, b2, c2, d2). The vertical line at 1095 days indicates the starting of the exposure period.

Trout Winter

a1

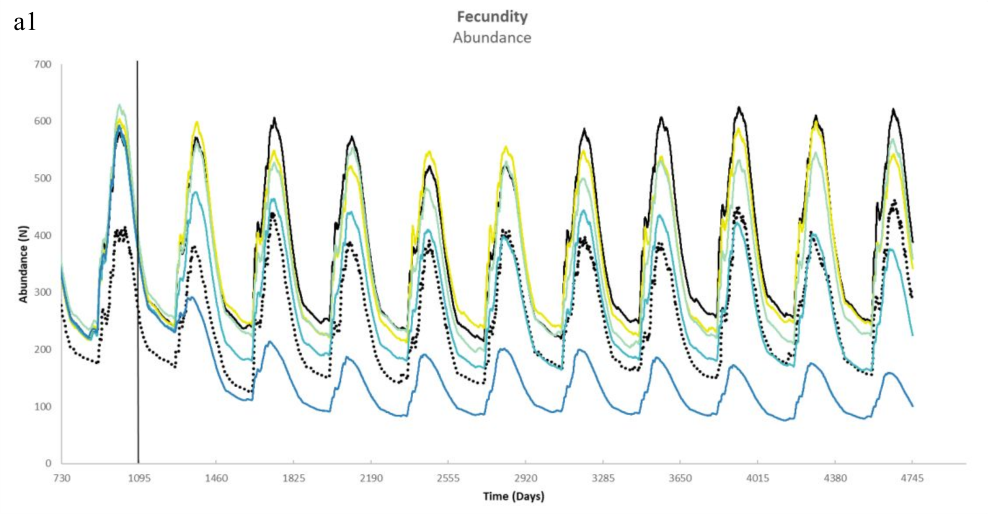

a2

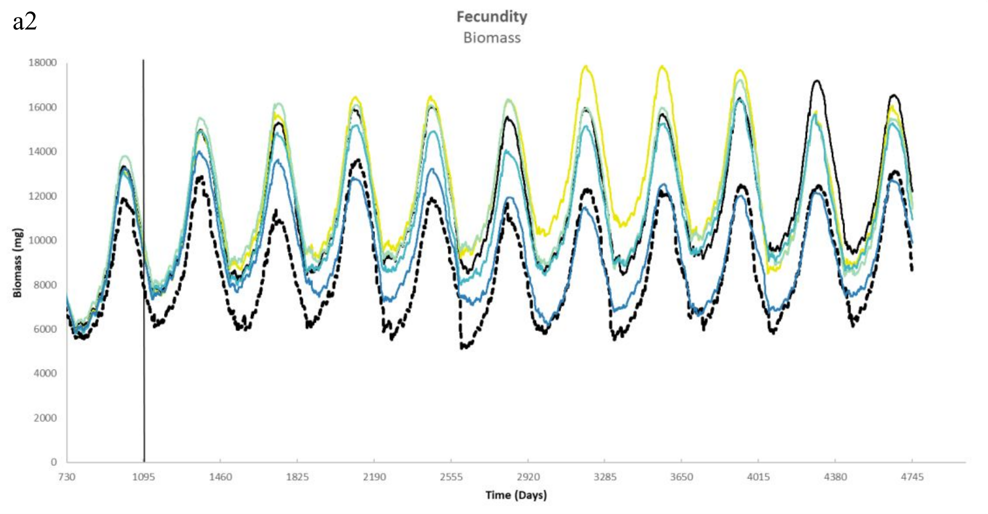

b1

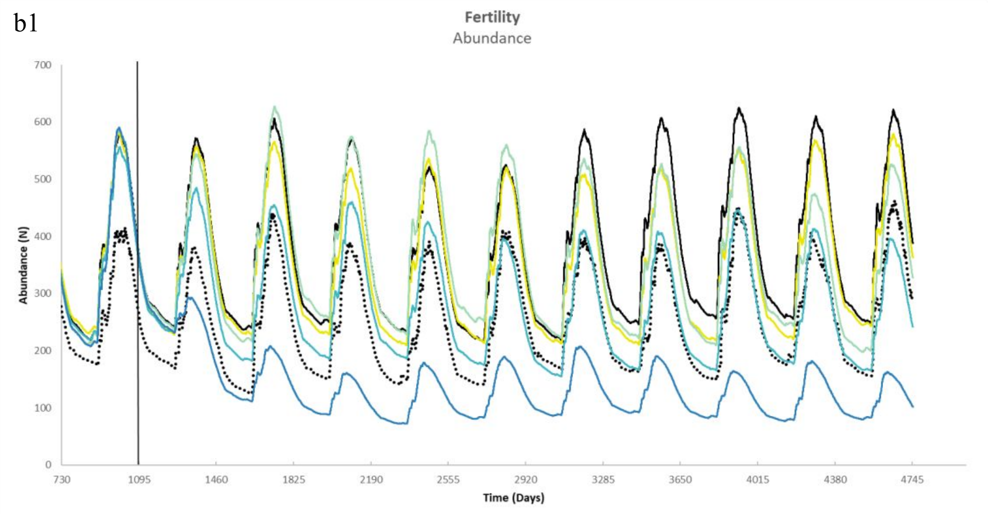

b2

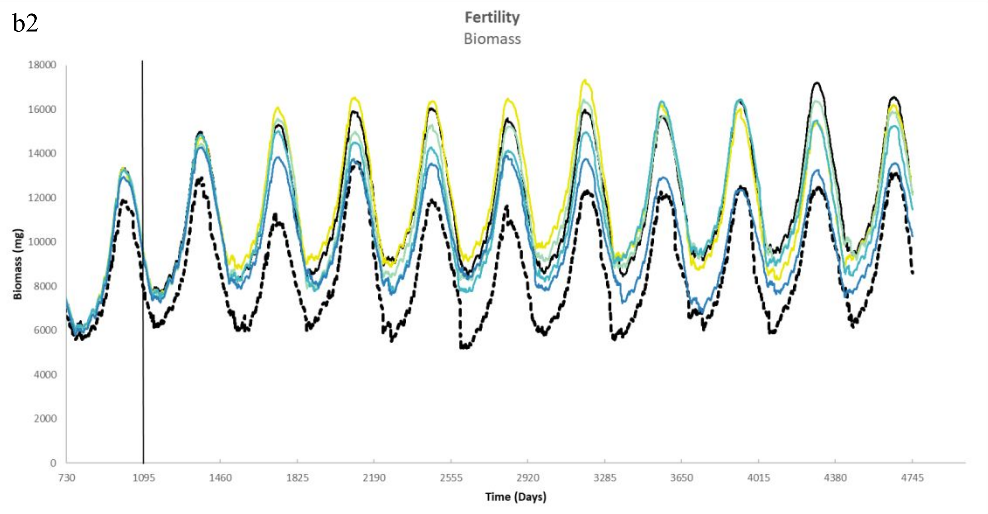

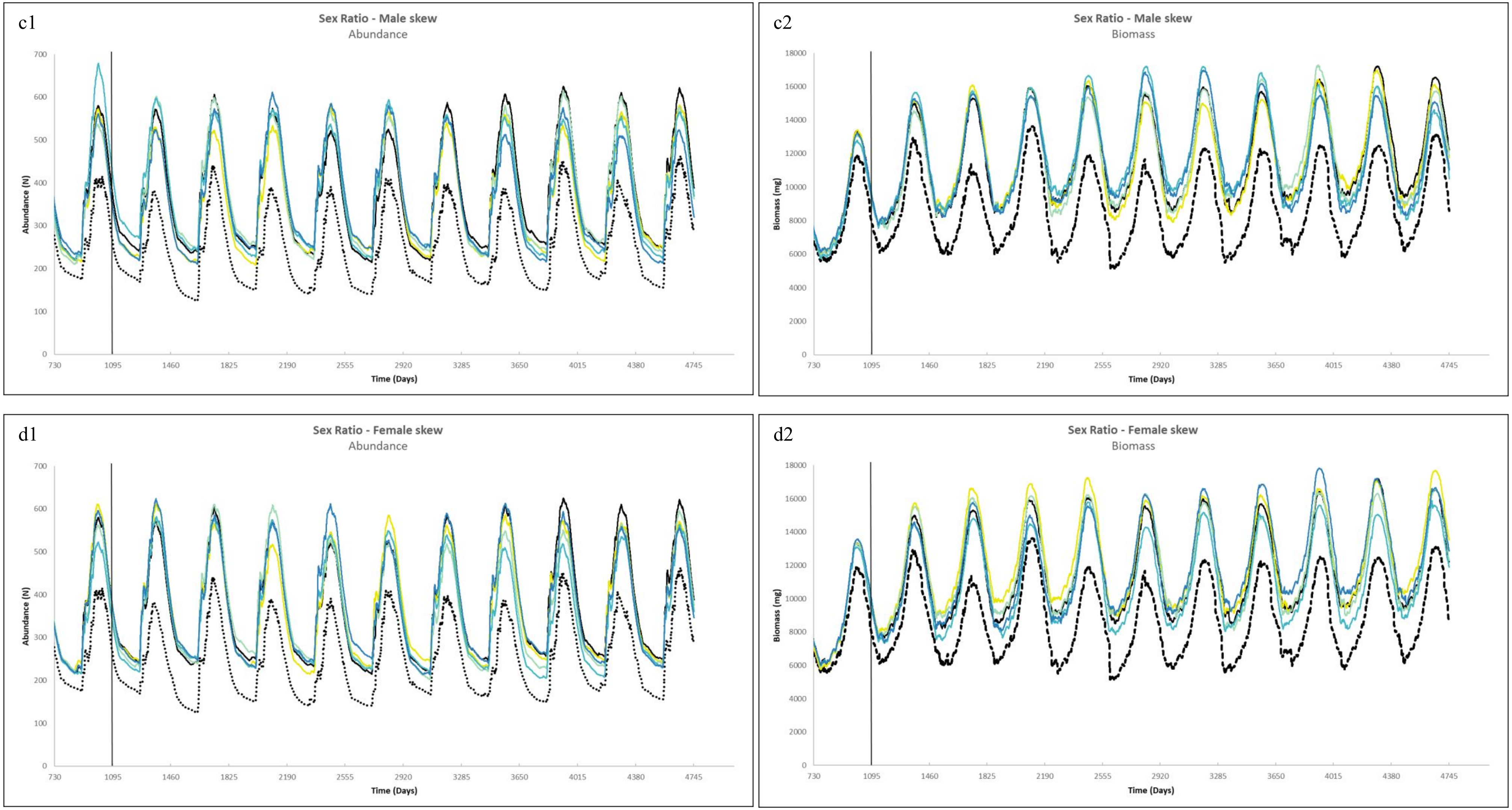

Figure S10: Population results plots for the trout 10-year simulations of seasonal effects in winter: fecundity, fertility, sex ratio male and female skew, for abundance (a1, b1, c1, d1) and biomass (a2, b2, c2, d2). The vertical line at 1095 days indicates the starting of the exposure period.

Graphs for the 10% combination of effects (fecundity, fertilisation rate, sex skew)

Legend: —— Control ········ Control Lower 95th %ile —— 10% effects (fecundity + fertility + sex ratio)

Zebrafish

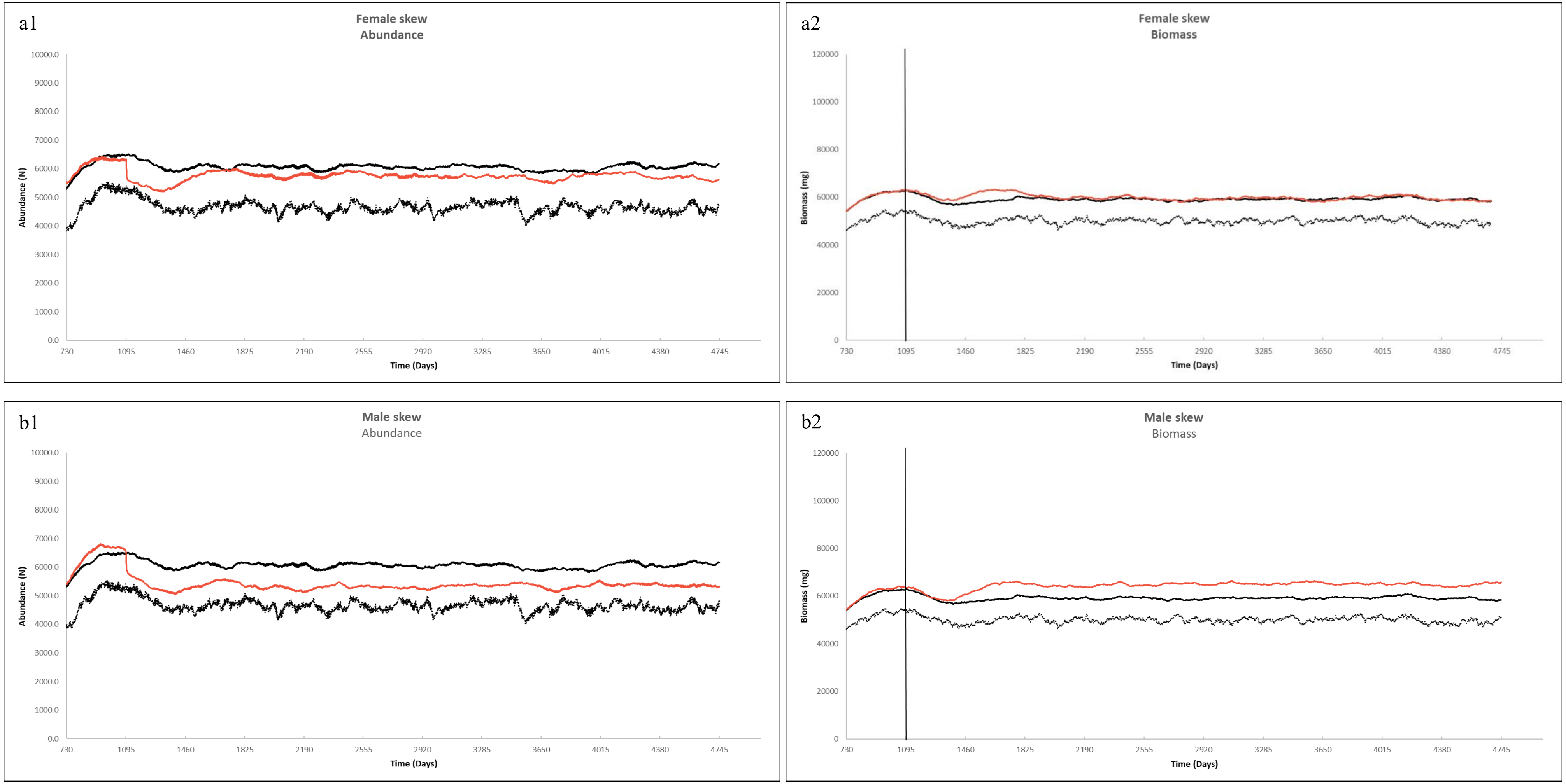

Figure S11: Population results plots for the zebrafish considering a 10% combination of fecundity, fertility and sex ratio male and female skew effects, for abundance (a1, b1) and biomass (a2, b2). The vertical line at 1095 days indicates the starting of the exposure period.

Stickleback

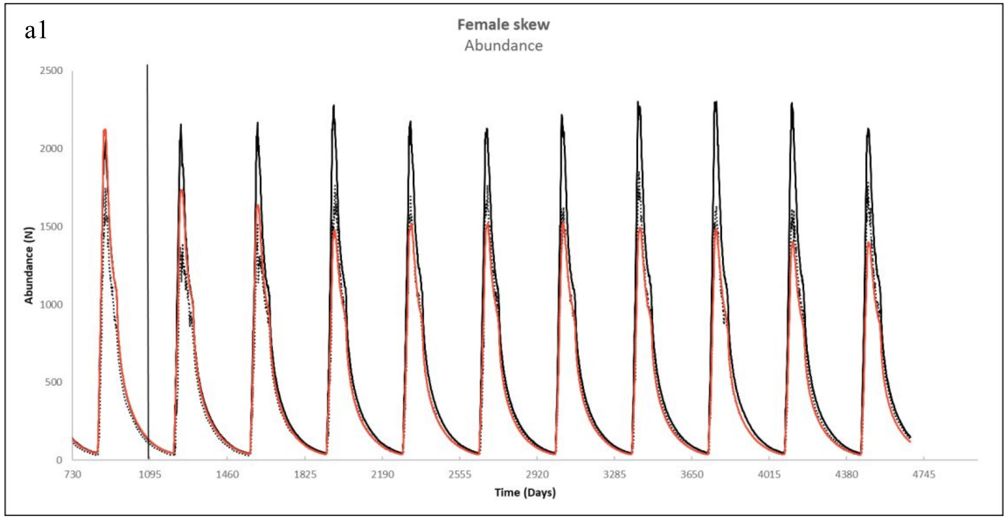

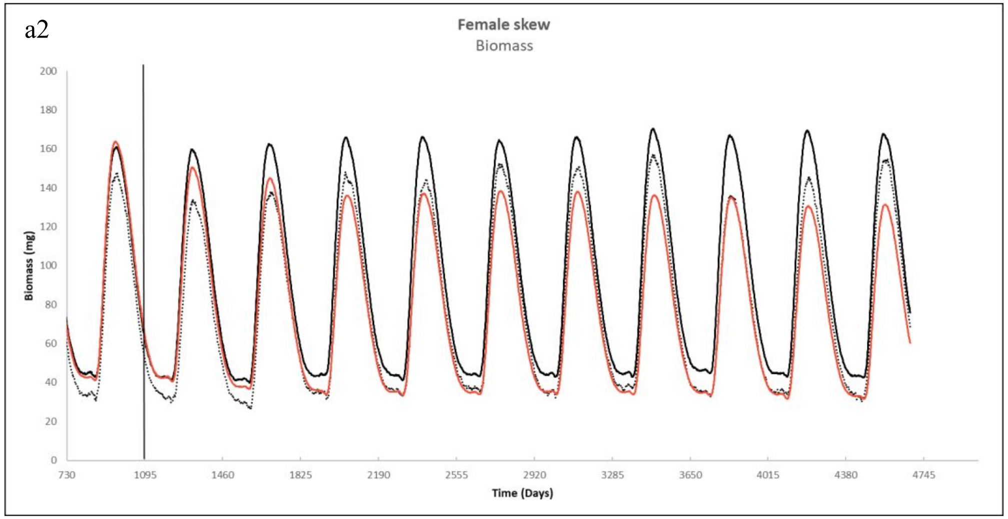

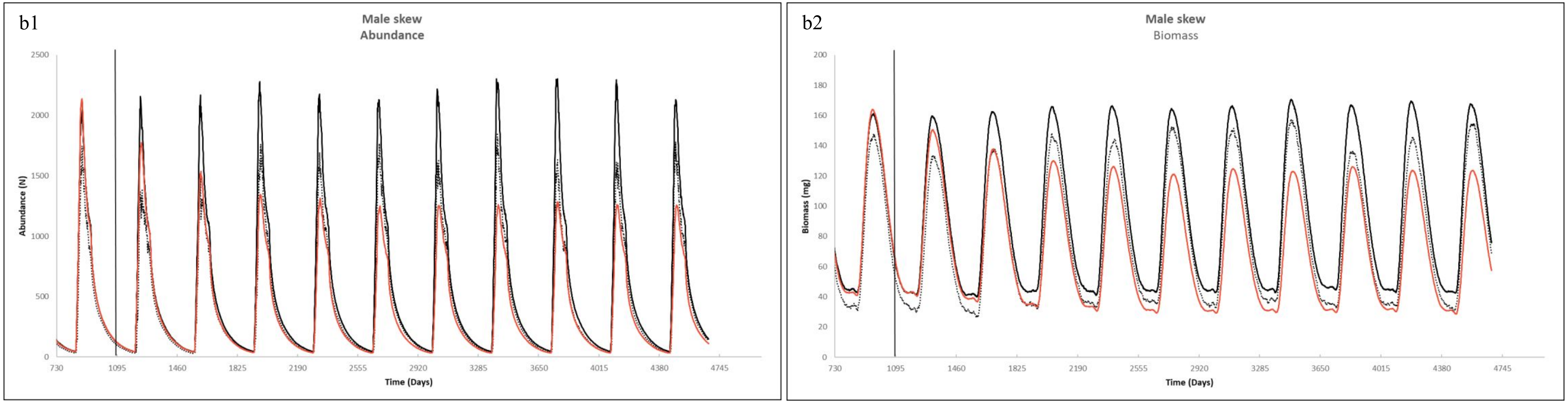

Figure S12: Population results plots for the stickleback considering a 10% combination of fecundity, fertility and sex ratio male and female skew effects, for abundance (a1, b1) and biomass (a2, b2). The vertical line at 1095 days indicates the starting of the exposure period.

Trout

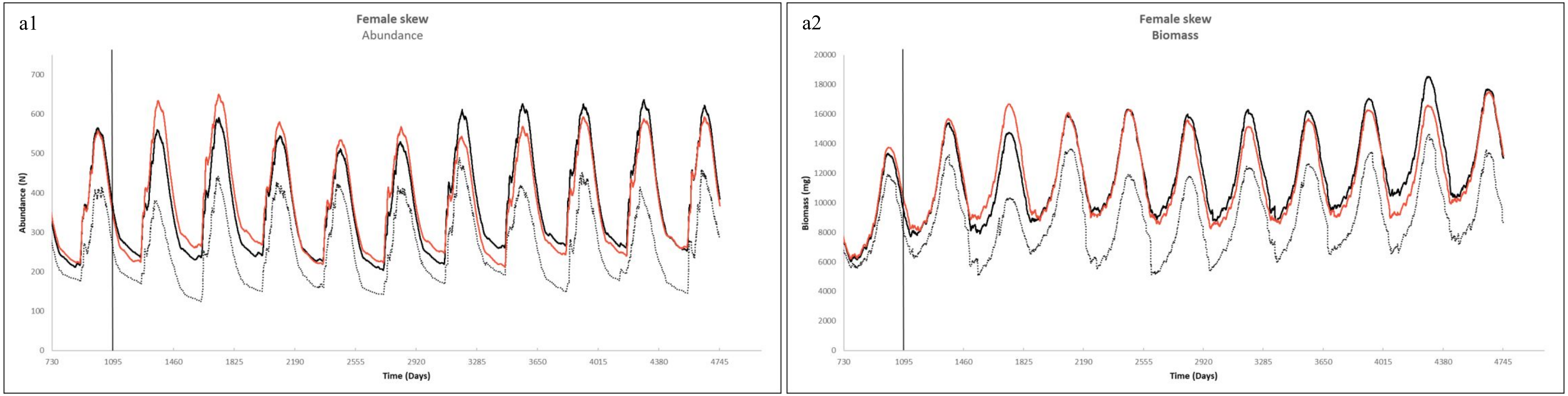

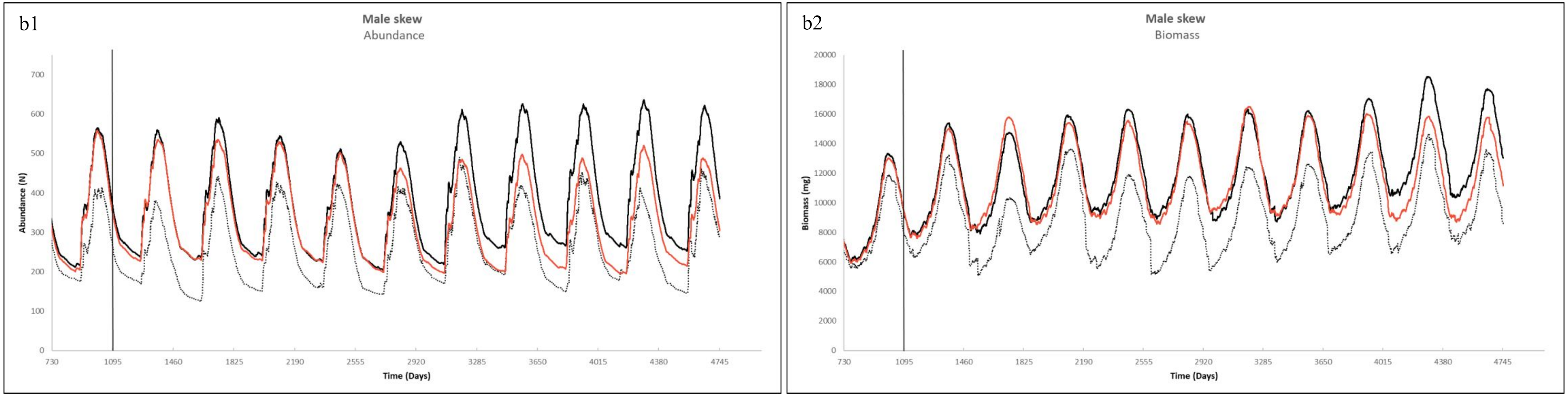

Figure S13: Population results plots for the trout considering a 10% combination of fecundity, fertility and sex ratio male and female skew effects, for abundance (a1, b1) and biomass (a2, b2). The vertical line at 1095 days indicates the starting of the exposure period.
